# Revision of the species of *Lytopylus* from Area de Conservación Guanacaste, northwestern Costa Rica (Hymenoptera, Braconidae, Agathidinae)

**DOI:** 10.3897/zookeys.721.20287

**Published:** 2017-12-12

**Authors:** Ilgoo Kang, Eric G. Chapman, Daniel H. Janzen, Winnie Hallwachs, Tanya Dapkey, M. Alex Smith, Michael J. Sharkey

**Affiliations:** 1 Department of Entomology, University of Kentucky, Lexington, KY 40546-0091, United States of America; 2 Department of Biology, University of Pennsylvania, Philadelphia, PA 19104-6018, United States of America; 3 Department of Integrative Biology, University of Guelph and Biodiversity Institute of Ontario, Guelph, Canada

**Keywords:** Costa Rica, DNA barcoding, host use, parasitoid wasps, species limits, taxonomy

## Abstract

Thirty two new species of *Lytopylus* (Agathidinae) are described with image plates for each species: *Lytopylus alejandromasisi* **sp. n.**, *Lytopylus alfredomainieri* **sp. n.**, *Lytopylus anamariamongeae* **sp. n.**, *Lytopylus angelagonzalezae* **sp. n.**, *Lytopylus cesarmorai* **sp. n.**, *Lytopylus eddysanchezi* **sp. n.**, *Lytopylus eliethcantillanoae* **sp. n.**, *Lytopylus ericchapmani* **sp. n.**, *Lytopylus gahyunae* **sp. n.**, *Lytopylus gisukae* **sp. n.**, *Lytopylus guillermopereirai* **sp. n.**, *Lytopylus gustavoindunii* **sp. n.**, *Lytopylus hartmanguidoi* **sp. n.**, *Lytopylus hernanbravoi* **sp. n.**, *Lytopylus hokwoni* **sp. n.**, *Lytopylus ivanniasandovalae* **sp. n.**, *Lytopylus johanvalerioi* **sp. n.**, *Lytopylus josecortesi* **sp. n.**, *Lytopylus luisgaritai* **sp. n.**, *Lytopylus mariamartachavarriae* **sp. n.**, *Lytopylus miguelviquezi* **sp. n.**, *Lytopylus motohasegawai* **sp. n.**, *Lytopylus okchunae* **sp. n.**, *Lytopylus pablocobbi* **sp. n.**, *Lytopylus robertofernandezi* **sp. n.**, *Lytopylus rogerblancoi* **sp. n.**, *Lytopylus salvadorlopezi* **sp. n.**, *Lytopylus sangyeoni* **sp. n.**, *Lytopylus sarahmeierottoae* **sp. n.**, *Lytopylus sergiobermudezi* **sp. n.**, *Lytopylus sigifredomarini* **sp. n.**, and *Lytopylus youngcheae* **sp. n.** A dichotomous key and a link to an electronic, interactive key are included. All specimens were reared from Lepidoptera larvae collected in Area de Conservación Guanacaste (ACG) and all are associated with ecological information including host caterpillar, collection date, eclosion date, caterpillar food plant, and locality. Neighbor-joining and maximum likelihood analyses of the barcode region of the mitochondrial cytochrome *c* oxidase subunit I gene (COI DNA barcode) were conducted to aid in species delimitation.

## Introduction

Agathidinae contains approximately 1,200 described species (Yu et al. 2012), making it a moderately species-rich subfamily of Braconidae. All members of Agathidinae are koinobiont endoparasitoids of Lepidoptera larvae (Sharkey 2006) meaning that they are internal parasitoids that enter early instar host larvae and their hosts continue to develop before being consumed in the last instar or prepupal parasitoid stage. Over most of its history, *Lytopylus* was considered a junior synonym of *Bassus* Fabricius, *Microdus* Nees, or *Agathis* Latreille. Sharkey et al. (2016) removed the genus from synonymy and synonymized *Agathellina* Enderlein, 1920, *Ditropia* Enderlein, 1920, and *Austroearinus* Sharkey, 2006, under it, thereby including six species in the genus, i.e., *Lytopylus azygos* Viereck, 1905, *Agathellina columbiana* Enderlein, 1920, *Austroearinus chrysokeras* Sharkey, 2006, *Austroearinus melanopodes* Sharkey, 2006, *Bassus rufofemoratus* Muesebeck, 1927, *Ditropia strigata* Enderlein, 1920, *Lytopylus unicolor* (Schrottky, 1902). Only one species of *Lytopylus* has a published host association: *L. unicolor* (Shenefelt 1970) is a parasitoid of the potato tuber-worm *Phthorimaea operculella* (Zeller 1873) in the Gelechiidae.

Although this article appears to be the second taxonomic revision of *Lytopylus* Forster, 1862 from Area de Conservación Guanacaste (ACG), the revision by Sharkey et al. (2011) employed the name *Lytopylus* in error and later Sharkey et al. (2016) transferred all of species described under *Lytopylus* in that paper to *Aerophilus* Szépligeti, 1902.

This work focuses on specimens of *Lytopylus* reared from Lepidoptera larvae collected by Drs Janzen and Hallwachs and the team of ACG parataxonomists since 1978 in the ACG (Janzen et al. 2009, Janzen and Hallwachs 2011, 2016). All are associated with ecological information including host caterpillar, collection date, eclosion date, caterpillar food plant, and locality. COI mitochondrial DNA barcodes for most specimens are deposited in the Barcode of Life Datasystem (BOLD) (http://www.boldsystems.org) (Hebert et al. 2003) and are equally available at http://janzen.sas.upenn.edu. The sequence data are publicly available through the Public Data Portal of BOLD (http://www.boldsystems.org/index.php/Public_BINSearch?searchtype=records).

We include an image plate for each species, a traditional identification key and a digital web-based interactive key; both have illustrations of morphological characters (https://www.dropbox.com/s/j9xongce1qrav5j/Revised%20Lytopylus%20Interactive%20key.zip?dl=0). A diagnoses and descriptions are provided for thirty-two new species and one previously described species.

## Methods

### Species concepts

We use Mayr’s (1969) biological species concept, i.e., a species consists of a group of natural populations that are reproductively isolated from other groups. Because insect taxonomists usually work with dead specimens, delimitation of insect species is based on methods that indirectly infer reproductive isolation rather than direct observation, i.e., similarity in morphological, molecular (COI DNA sequences), geography, and host use data if recorded.

### Specimen information

Most specimens, and all holotypes, are deposited in the insect collection in the Biology Department of Utah State University (USU) Logan, Utah. Duplicates are in the Hymenoptera Institute Collection (HIC), Entomology Department, University of Kentucky and those from ACG will eventually be deposited in a major North American Museum. The detailed parasitoid specimen records are available by search of the individual specimen DHJPARxxxxxxx voucher codes on Janzen’s database (http://janzen.sas.upenn.edu/caterpillars/database.lasso). Host caterpillars are uniquely identified by their own voucher code system, which is recognizable by YY-SRNP-XXXXX where “YY” is the two-digit year and “XXXXX” is a unique number within that year. Some of the host caterpillars are incompletely identified, but they also have unique names such as *Dichomeris* Janzen512, which is an interim name for *Dichomeris* species 512 as determined by a biodiversity specialist of the ACG team or a professional taxonomist who provides the proper genus epithet. These names will be updated in the database when the species is blessed with a formal scientific name, but the interim name, in this case *Dichomeris* Janzen512, will remain searchable in that database.

### Morphological analysis

Morphological characters were recorded using the DELTA Editor (v. 1.02; Dallwitz et al. 1999). The DELTA Editor was used to enter the data for both interactive (web-based) and traditional printed keys (https://www.dropbox.com/s/j9xongce1qrav5j/Revised%20Lytopylus%20Interactive%20key.zip?dl=0). Images to illustrate the couplets were taken by a JVC digital camera fixed on microscopes and stacked with the program Automontage. Plates for each species were arranged using Adobe Photoshop Elements 12. The morphological terms mostly follow Sharkey and Wharton (1997) and are coordinated with the Hymenoptera Anatomy Ontology (HAO, Yoder et al. 2010). The minimum number of characters necessary to distinguish a species from all other species in this study is included in a diagnosis for each species. Descriptions, based on the holotype of each species, were automatically generated using DELTA.

### DNA extraction, PCR, and sequencing

271 COI DNA sequences were sourced from the BOLD database. DNA was extracted by the Centre for Biodiversity Genomics using a glass fibre protocol (Ivanova et al. 2006). Extracts were resuspended in 30 μL _d_H_2_O, and a 658-bp region near the 5’ terminus of the CO1 gene was ampliﬁed using standard insect primers LepF1 (5’-ATTCAACCAATCATAAAGATATTGG-3’) and LepR1 (5’-TAAACTTCTGGATGTCCAAAAAATC A-3’) following the established protocols (Smith et al. 2008). If initial amplification failed, other ampliﬁcations were conducted following the established protocols using internal primer pairs, LepF1-C113R (130 bp) or LepF1-C_ANTMR1D (300 bp) and MLepF1-LepR1 (400 bp) to generate shorter overlapping sequences (Smith et al. 2008).

For the specimens which DNA sequences were not available in BOLD, DNA was extracted from individual legs at University of Kentucky (UKY) with Qiagen DNeasy Blood and Tissue Kit following the manufacturer’s animal tissue protocol (Qiagen Inc., Chatsworth, California, USA).

COI was amplified from extracted DNA using the forward primer mlCOIintF (Leray et al. 2013) and reverse primer jgHCO2198 (Geller et al. 2013). Unique 9 bp tags, designed using Barcode Generator (available from http://comailab.genomecenter.ucdavis.edu/index.php/Barcode_generator) were attached to the primers so that each sequence could be traced to its parent specimen by the unique combination of tags. PCR was performed using Takara reagents consisted of 10X buffer, 2.5 μM nucleotides, 1 μM of each primer, 0.125 U Takara Ex Taq, 2 μL template DNA and enough _dd_H_2_O for a total reaction volume of 25 μL. We followed the “touchdown” thermal cycling protocol for these primers as outlined in Leray et al. (2013).

COI PCR DNA products, in addition to those from BOLD, were sequenced on an Illumina MiSeq system at the UKY Genomics Core Laboratory.

### DNA assembly and phylogenetic analysis

Individual directional reads were downloaded from BOLD (produced by Sanger sequencing) and were edited and assembled using Geneious Pro (v. 6.1.6; Drummond et al. 2010) with the default settings. Edited sequences were stored in the NEXUS file format. The three sequences produced by NGS at UKY were included in the file of edited sequences. NGS sequencing data was assembled using PEAR (Zhang et al. 2013) and demultiplexed using custom Phython scripts. Among all bidirectional reads from each specimen, the 1^st^ and 2^nd^ most numerous reads were manually retrieved from the output file. The sequences were then queried against the GenBank nucleotide library using NCBI BLAST (https://blast.ncbi.nlm.nih.gov/Blast.cgi) and those that were highly similar to *Lytopylus* specimens were retained. Finally, three COI sequences were exported from the FASTQ file and added to the file of edited sequences. The multiple sequence alignment was assembled on the MAFFT server (http://www.ebi.ac.uk/Tools/msa/mafft/; v. 7; Katoh et al. 2013) using the default settings.

A NJ tree (Saitou and Nei 1987) was constructed by using PAUP* (v. 4.0β10; Swofford 2003) using the p-distance setting. ML analyses were performed using Garli (v. 2.01; Zwickl 2006). For ML, the data were partitioned by codon position for COI (three partitions). We applied the most complex model available (GTR+I+G; Rodriguez et al. 1990) to each partition as per recommendations of Huelsenbeck and Rannala (2004) for likelihood-based analyses. Garli applies separate parameter estimates to each partition. A 20-replicate ML analysis was performed using default settings. Additionally, a ML bootstrap analysis (minimum 500 replicates) was conducted to assess nodal support (Garli, default settings). The COI data set analyzed herein is available from the senior author upon request.

### Host use

Besides the notes included here additional data can be accessed at http://janzen.sas.upenn.edu (Janzen et al. 2009).

### Species delimitation

**Figure 1.**
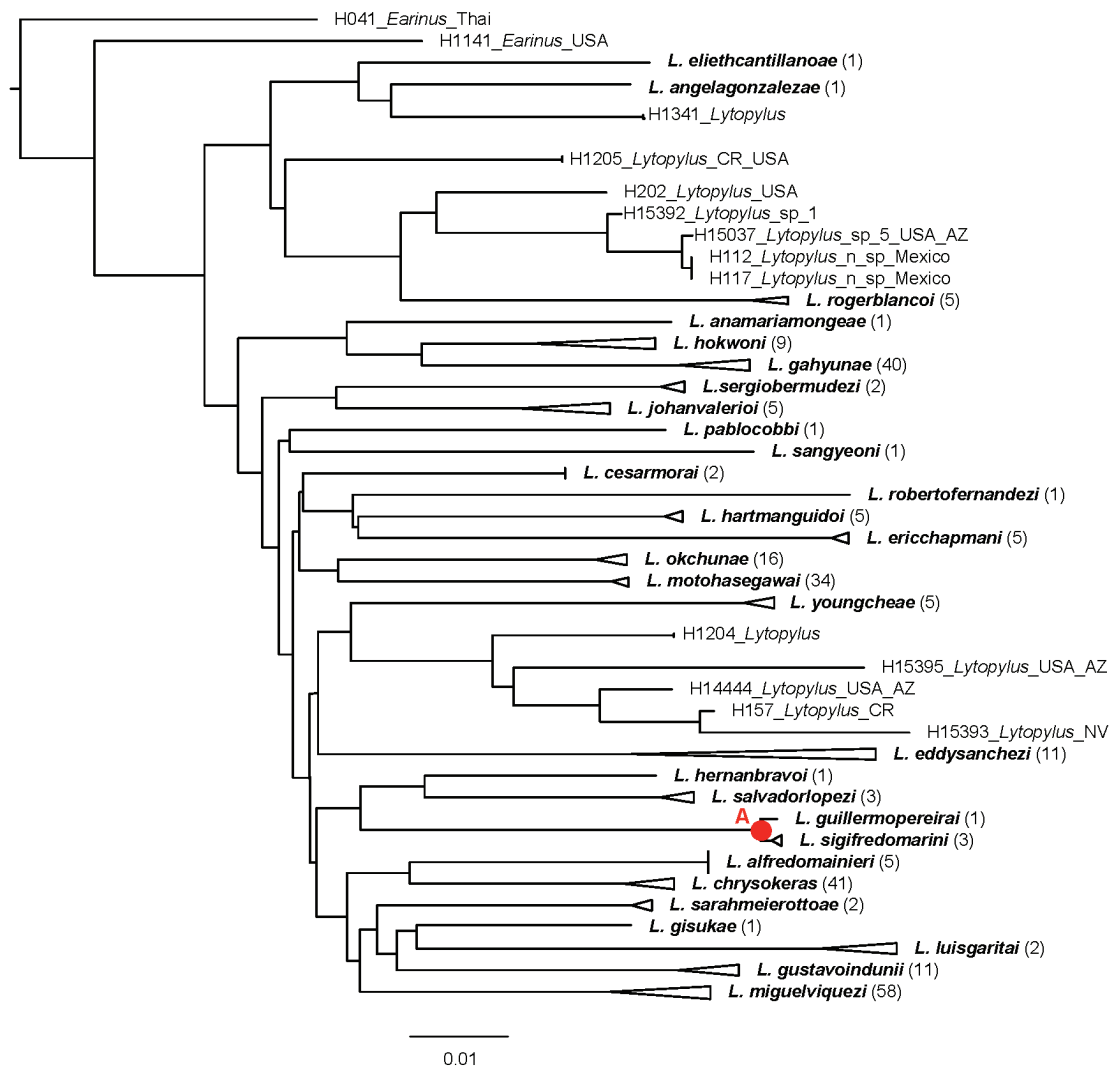
The NJ tree of the COI DNA barcode region for twenty-nine of the thirty-three *Lytopylus* species treated here. Triangles represent collapsed clades; their lengths (measured horizontally) represent the distance from the most basal node to the apex of the longest branch. The number of specimens in each triangle is given in parentheses following the species name. The node labeled with a red “**A**” is discussed in the text.

**Figure 2.**
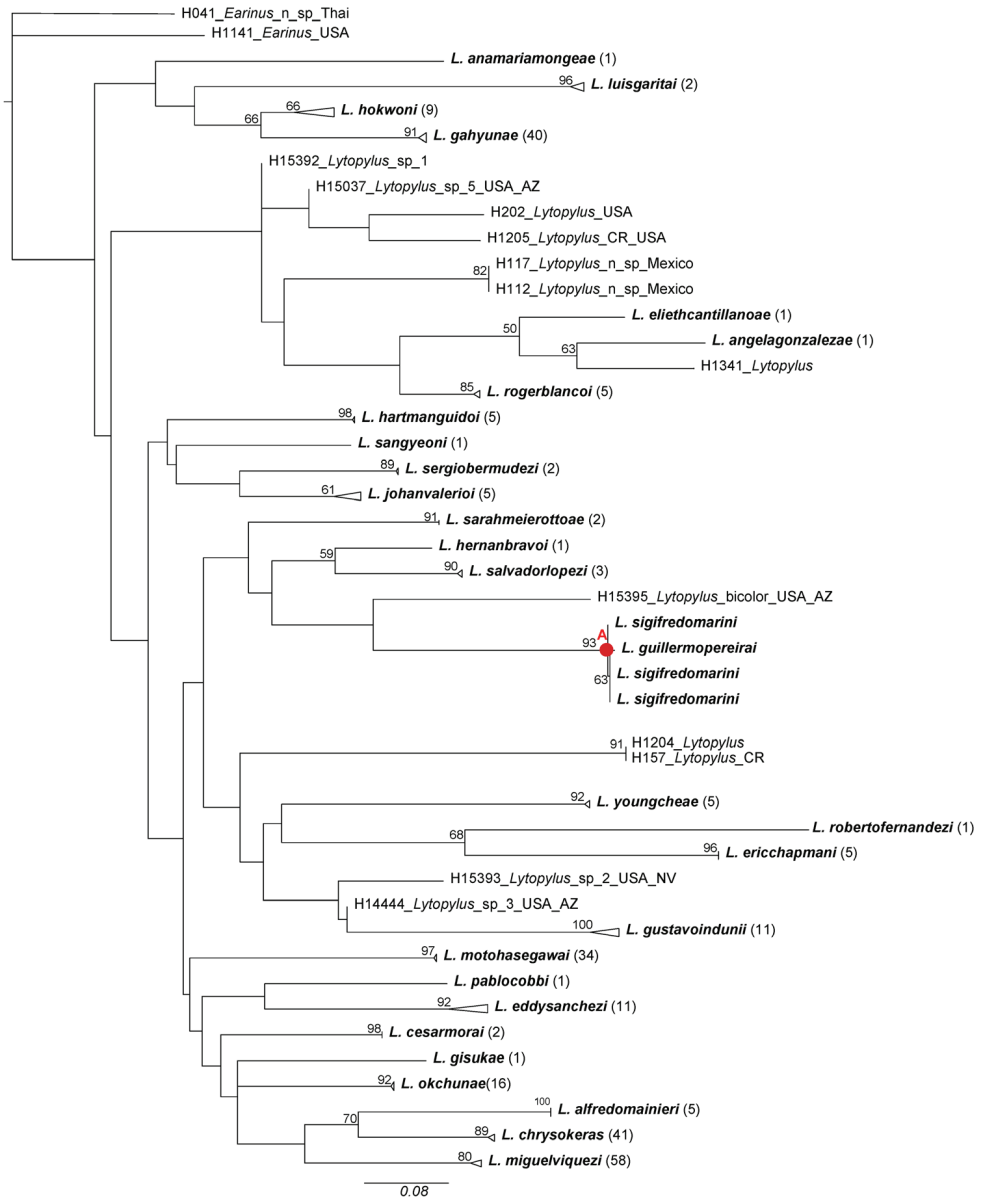
Tree of highest log-likelihood from 20 ML search reps of the COI data set. Terminals with bold-faced type indicate species described herein. ML bootstrap values appear above the branches. Triangles represent collapsed clades; their lengths (measured horizontally) represent the distance from the most basal node to the apex of the longest branch. The number of specimens in each triangle is given in parentheses following the species name. The node labeled with a red “A” is discussed in the text.

The NJ tree and the tree of highest log-likelihood from 20 ML search reps in Fig. 1 and Fig. 2 were based solely on COI. These trees were constructed solely to assist in the delimitation of species. Molecular species concepts were initially based on the NJ tree and were compared to the best ML tree with COI data. The 2% genetic distance cut-off, which has been a conventional threshold for species delimitation using COI barcodes (Jones et al. 2011) and has been used in the Barcode Index Numbers (BINs) (http://www.barcodinglife.org/index.php/Public_BarcodeIndexNumber_Home), was used to cluster putative species (Smith et al. 2013). Morphological and host use data were then employed to make final decisions when genetic distances between putative molecular species were near the 2% threshold or below it, as is necessary for other groups of insects (e.g., Janzen et al. 2017).

## Results and discussion

### Species delimitation

The NJ tree and the highest log-likelihood ML tree with COI data both suggest twenty-eight molecular species, and twenty-eight putative species were clustered using the 2% genetic distance cut-off.

Before running the molecular analyses I.K. and M.S. independently sorted the specimens to morphospecies and had error rates of 62% and 54% respectively based on our final species delimitations. All possible types of errors were discovered, i.e., clumping, splitting, and both clumping and splitting (mixing the members of two or more species). In contrast the molecular species concepts matched with our final species delimitations at 96.6%. The implications for previous taxonomic treatments of braconids (and other speciose small tropical insects) based solely on morphology are dire.

The sole incongruity between molecular species concepts and final species concepts concerned *L. sigifredomarini* and *L. guillermopereirai*. The genetic variation between these two species was 0.4%, and the ML tree grouped them together (Fig. 2, node A). We, however, delimited them as separate species because they are morphologically distinct in their strikingly different color patterns, and the NJ tree recovered these two species as monophylic sister taxa. (Fig. 1, node A). In addition, they attack different species of host caterpillars with different feeding niches.

The four species (*L. alejandromasisi*, *L. ivanniasandovalae*, *L. josecortesi*, *L. mariamartachavarriae*) for which genetic data were not available were delimited using morphological and host data.

### Systematics

#### Lytopylus

Taxon classificationAnimaliaHymenopteraBraconidae

Föster, 1862

Agathellina Enderlein, 1920. Type species: Agathellina columbiana Enderlein, 1920.
Ditropia Enderlein, 1920. Type species: Ditropia strigata Enderlein, 1920.
Austroearinus Sharkey, 2006. Type species: Bassus rufofemoratus Muesebeck, 1927.

##### Type species.

*Lytopylus azygos* Viereck, 1905, by monotypy, first included species.

##### Diagnosis.

*Lytopylus* can be distinguished from all other agathidine genera with the following combination of characters: tarsal claws simple with a basal lobe; mesoscutum unsculptured and notauli absent; fore wing vein (RS+Ma) not complete; vein CUb of hind wing weak or absent and never tubular; hind coxal cavities open; median tergite 3 smooth.

##### Distribution.

Restricted to the New World, from the northeastern USA south to Argentina, primarily Neotropical.

##### Species diversity.

Including the thirty-two species described here, there are 39 described species of *Lytopylus*. Based on the diversity in the University of Kentucky Hymenoptera Institute Collection, there are hundreds more awaiting description.

**Key to the species of *Lytopylus* of Area de Conservación Guanacaste, Costa Rica**

**Table:** 

1	A. Fore wing mostly or entirely infuscated	**2**
–	B. Fore wing hyaline or with a slight yellow tinge	**28**
–	C. Fore wing with one apical black band	**31**
–	D. Fore wing with two black bands	**33**
	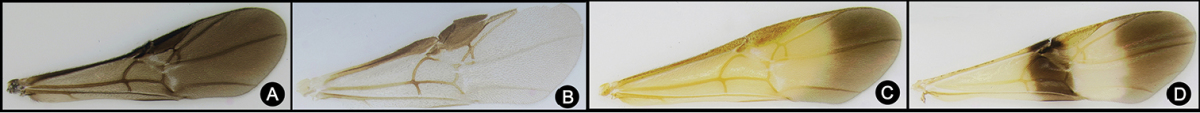	
2(1)	A. Median tergites mostly or entirely melanic (brown to black)	**3**
–	B. Median tergites entirely pale (yellow to orange) or mostly pale with posterior terga black	**10**
	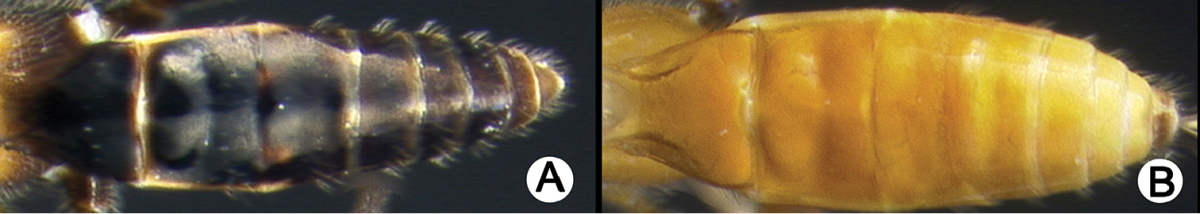	
3(2)	A. Scutellar sulcus with at least one longitudinal carina	**4**
–	B. Scutellar sulcus lacking longitudinal carinae	**6**
	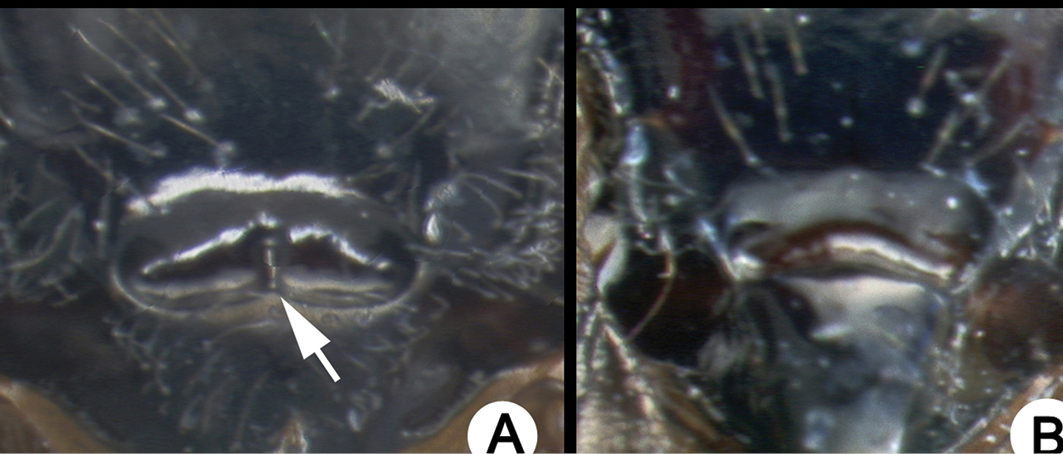	
4(3)	A. Lateral tergites one and two entirely white	***L. cesarmorai***
–	B. Lateral tergites one and two mostly or entirely yellow	**5**
	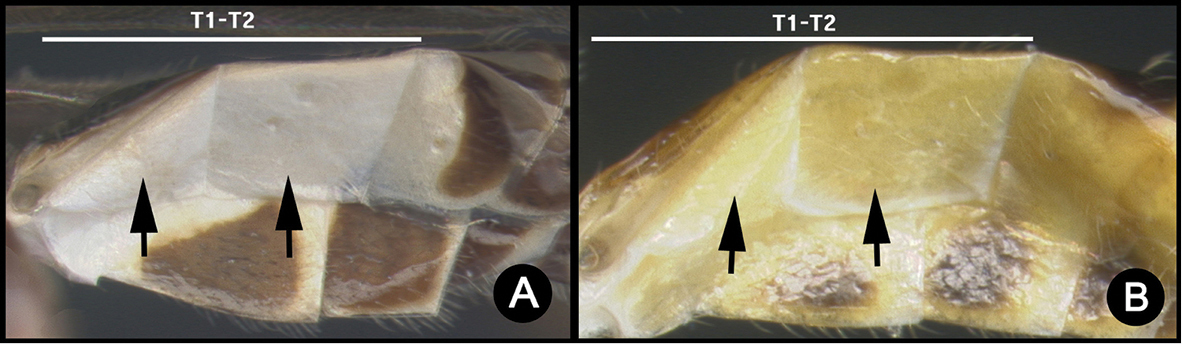	
5(4)	A. Hind femur mostly pale (yellow to orange) or black and pale with a similar percentage of each color	***L. motohasegawai*** ♂
–	B. Hind femur mostly black, pale apically	***L. miguelviquezi*** ♂
	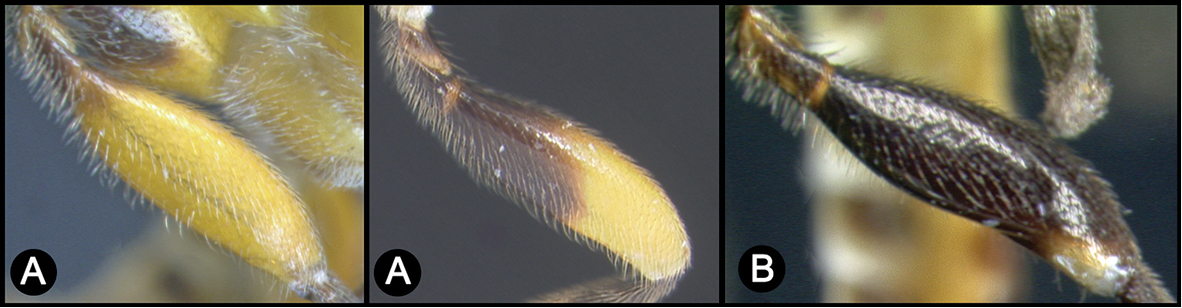	
6(3)	A. Mesoscutum mostly or entirely melanic (brown to black)	***L. guillermopereirai***
–	B. Mesoscutum mostly or entirely pale (yellow to orange)	**7**
	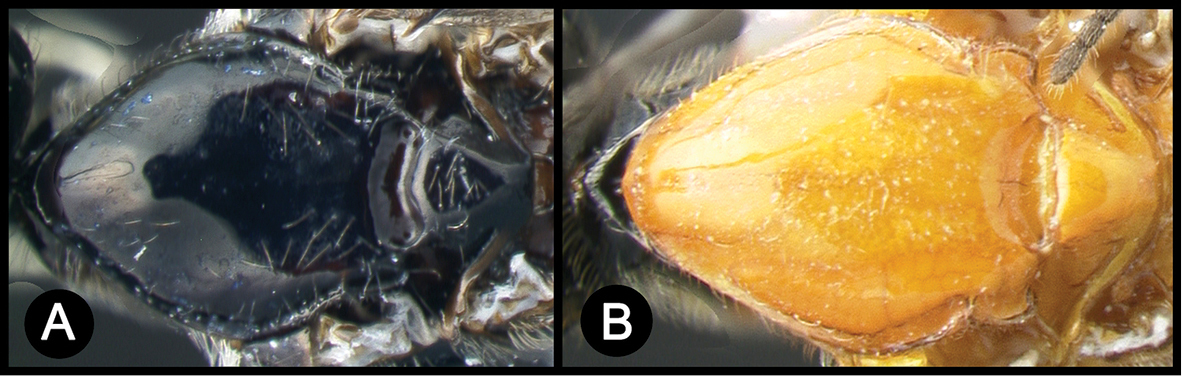	
7(6)	A. Pronotum entirely pale (yellow to orange)	**8**
–	B. Pronotum bicolored	**9**
	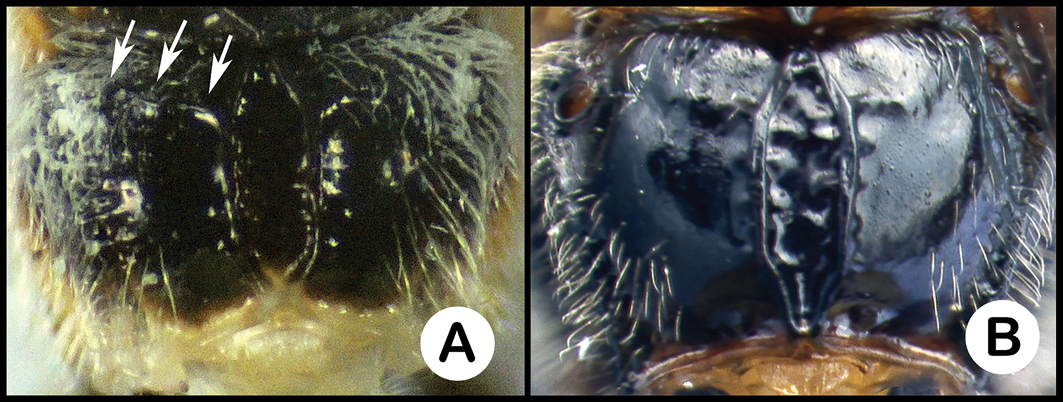	
8(7)	A. Anterior transverse carina of propodeum reaching the lateral margin	***L. sarahmeierottoae***
–	B. Anterior transverse carina of propodeum not reaching the lateral margin or absent	***L. salvadorlopezi***
	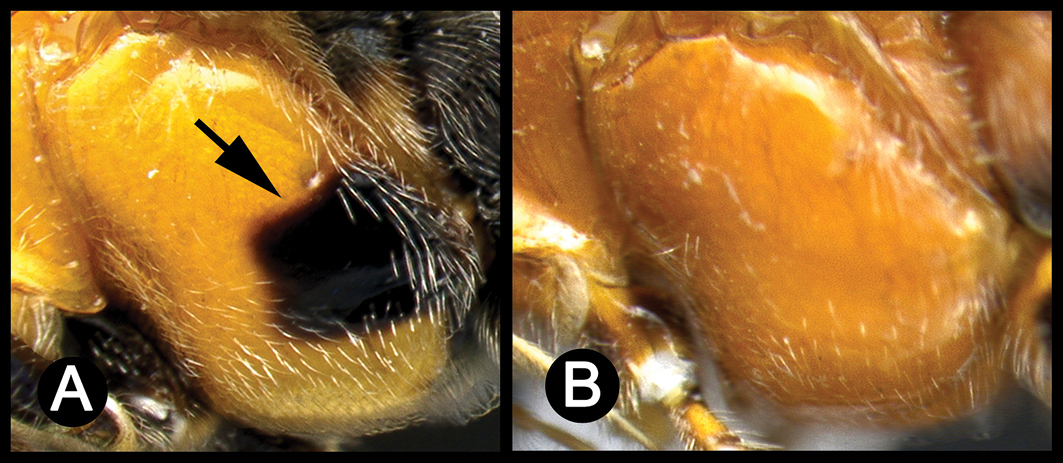	
9(7)	A. Mesopleuron bicolored	***L. anamariamongeae***
–	B. Mesopleuron entirely pale (yellow to orange)	***L. luisgaritai***
	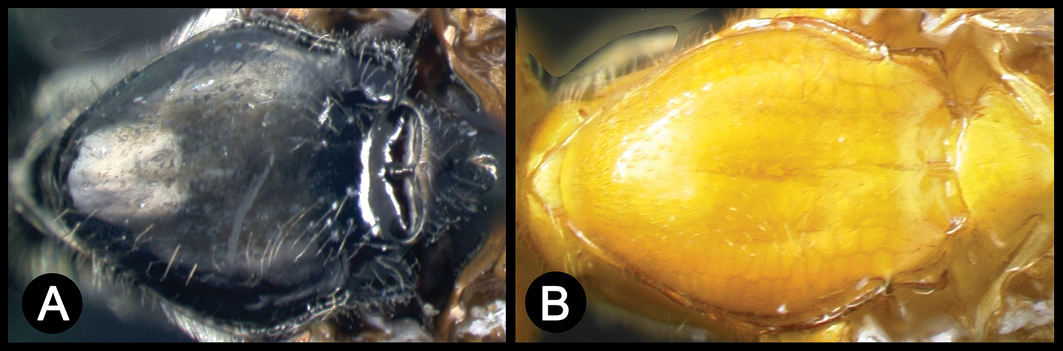	
10(2)	A. Mesoscutum mostly or entirely melanic	**11**
–	B. Mesoscutum mostly or entirely pale (yellow to orange)	**18**
	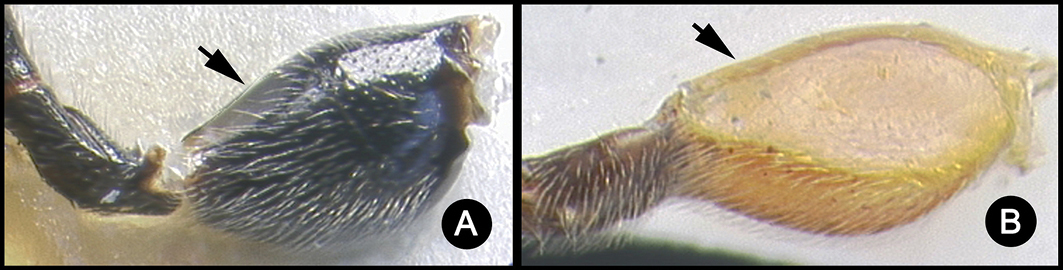	
11(10)	A. Anterior transverse carina of propodeum reaching the lateral margin	**12**
–	B. Anterior transverse carina of propodeum not reaching the lateral margin or absent	**15**
	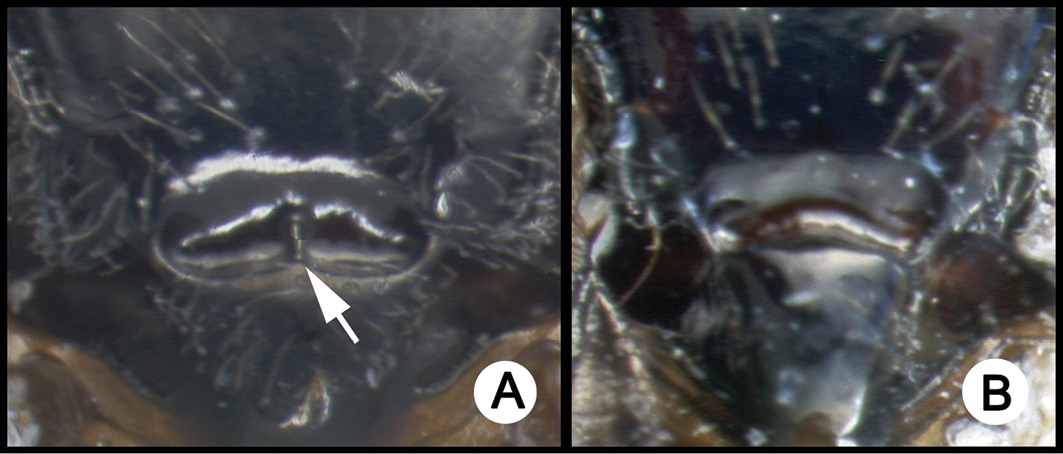	
12(11)	A. Hind coxa entirely black	***L. hernanbravoi***
–	B. Hind coxa mostly or entirely pale	**13**
	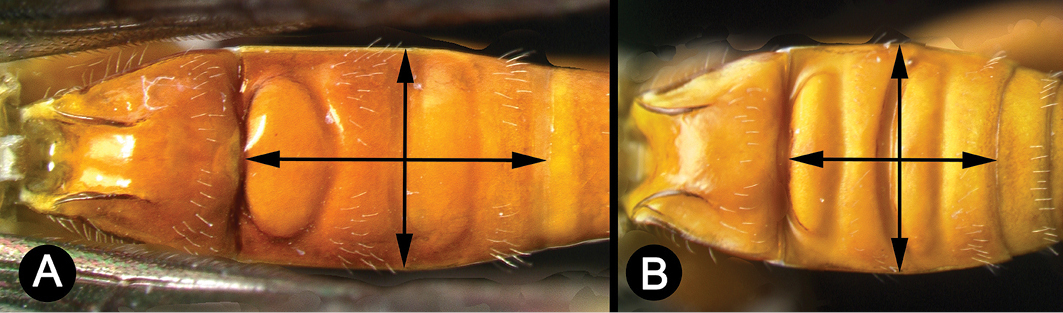	
13(12)	A. Scutellar sulcus with at least one longitudinal carina	**14**
–	B. Scutellar sulcus lacking longitudinal carinae	***L. sigifredomarini***
	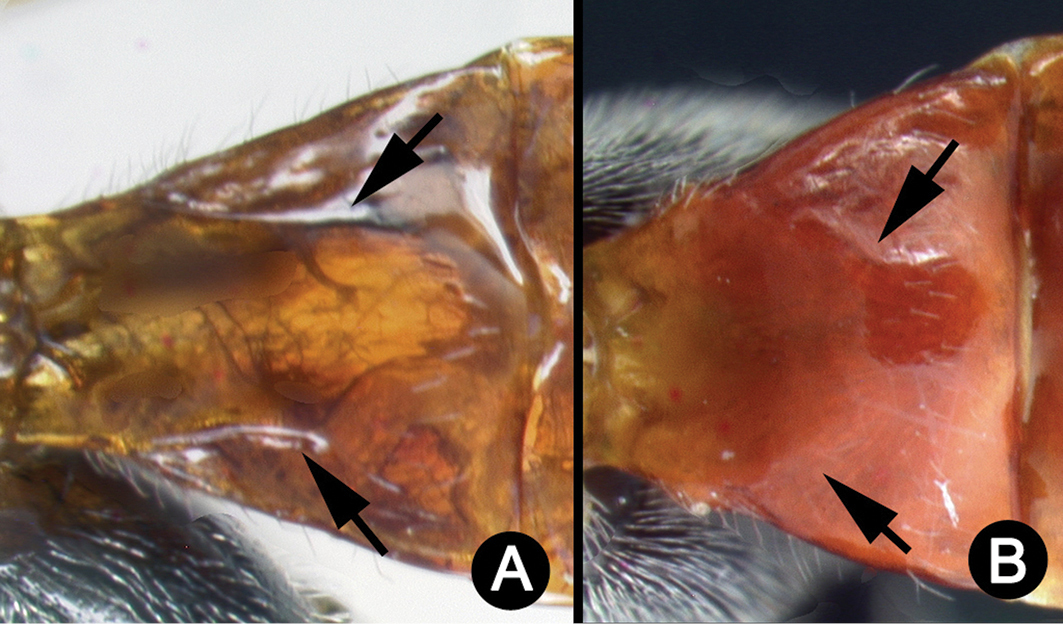	
14(13)	A. Median syntergite 2+3 1.4× longer than wide	***L. gahyunae***
–	B. Median syntergite 2+3 as long as wide	***L. sangyeoni***
	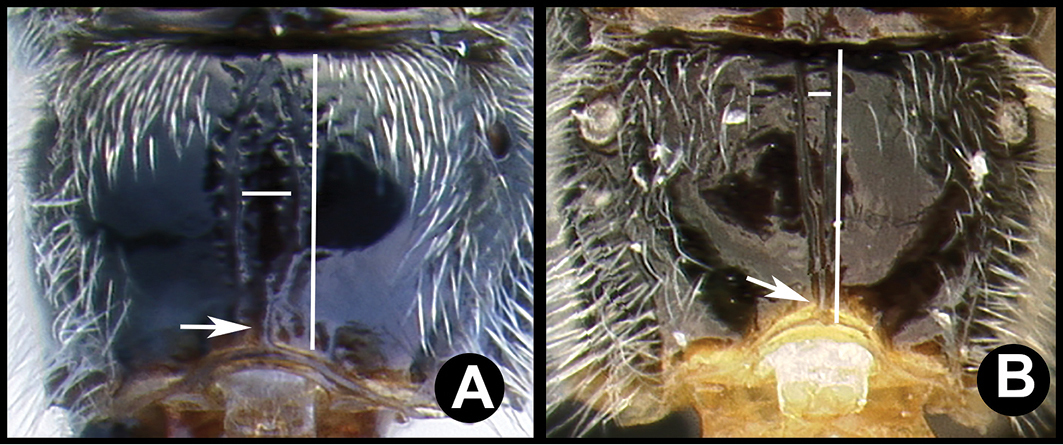	
15(11)	A. Lateral longitudinal carinae of median tergite 1 well-defined	**16**
–	B. Lateral longitudinal carinae of median tergite 1 blunt	**17**
	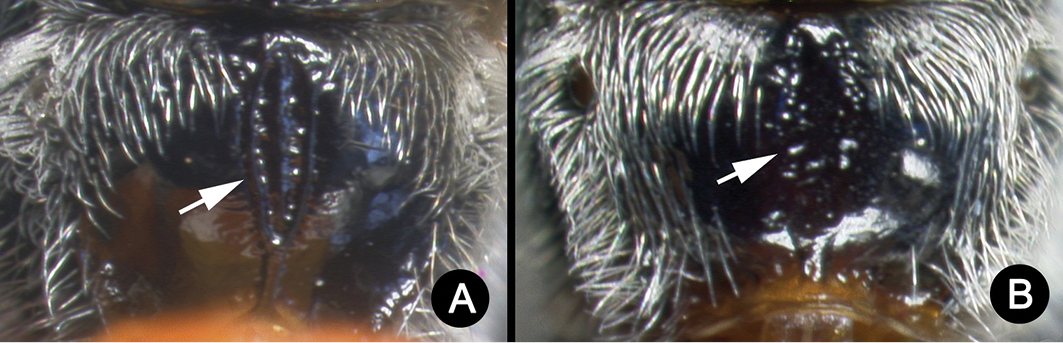	
16(15)	A. Median areola of propodeum spindle-shaped; median areola length 6× its width; median areola closed posteriorly	***L. josecortesi***
–	B. Median areola of propodeum wide anteriorly narrow and not closed posteriorly; median areola length 11× its width	***L. eliethcantillanoae***
	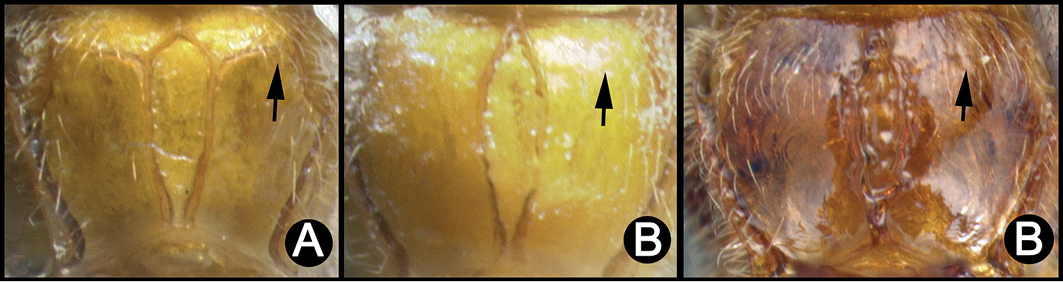	
17(15)	A. Median areola of propodeum with well-defined margins	***L. rogerblancoi***
–	B. Median areola of propodeum lacking well-defined margins	***L. angelagonzalezae***
	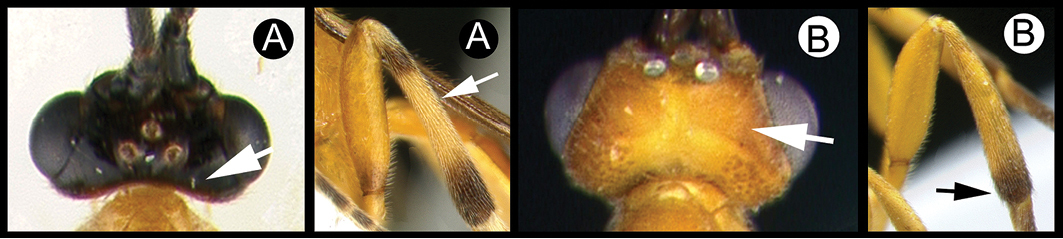	
18(10)	A. Anterior transverse carina of propodeum reaching the lateral margin	**19**
–	B. Anterior transverse carina of propodeum not reaching the lateral margin or absent	**21**
	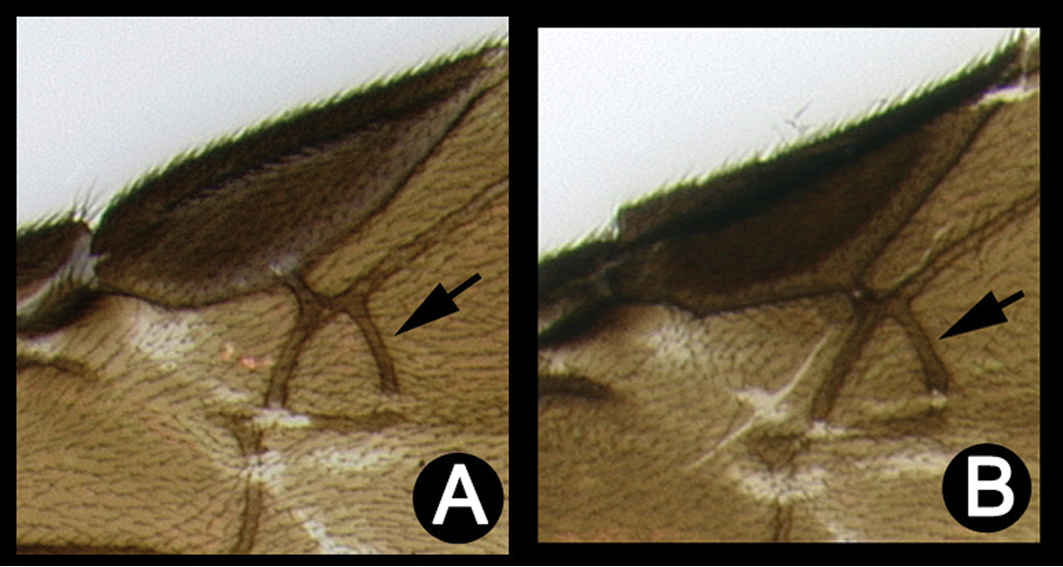	
19(18)	A. Pronotum entirely pale (yellow to orange)	**20**
–	B. Pronotum bicolored	***L. johanvalerioi***
	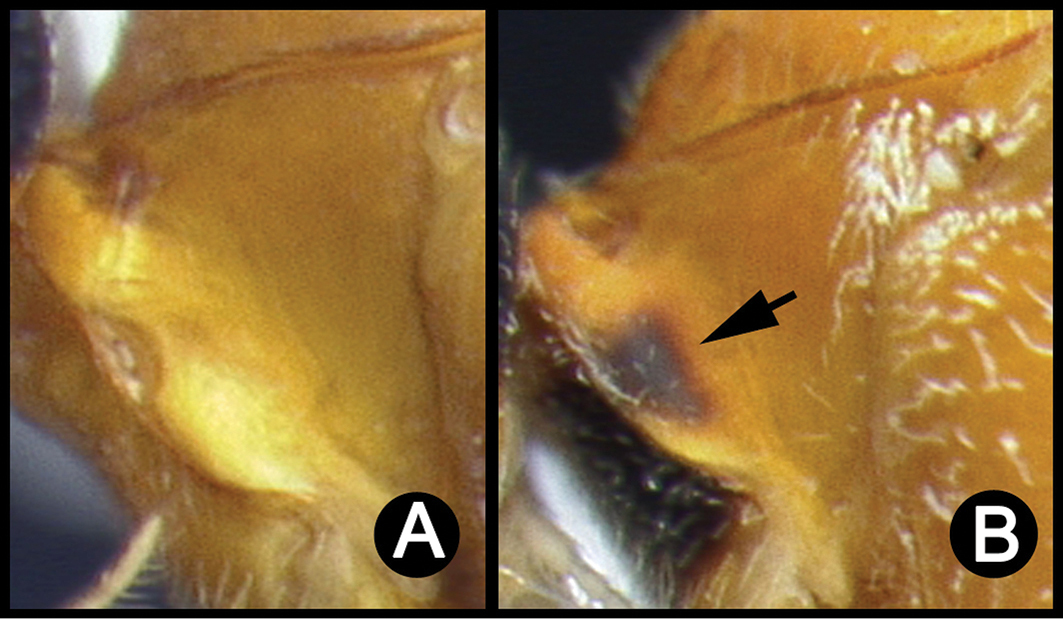	
20(19)	A. Vertex of head entirely melanic; hind tibia black basally and distally, yellow at mid-length	***L. alejandromasisi***
–	B. Vertex of head mostly or entirely yellow; hind tibia pale basally, black apically	***L. chrysokeras***
	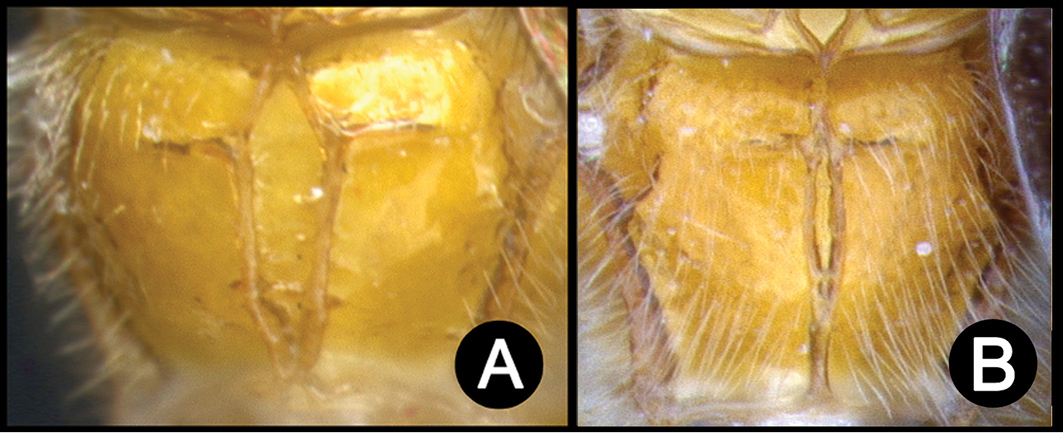	
21(18)	A. Fore wing second submarginal cell weakly quadrate	**22**
–	B. Fore wing second submarginal cell triangular	**24**
	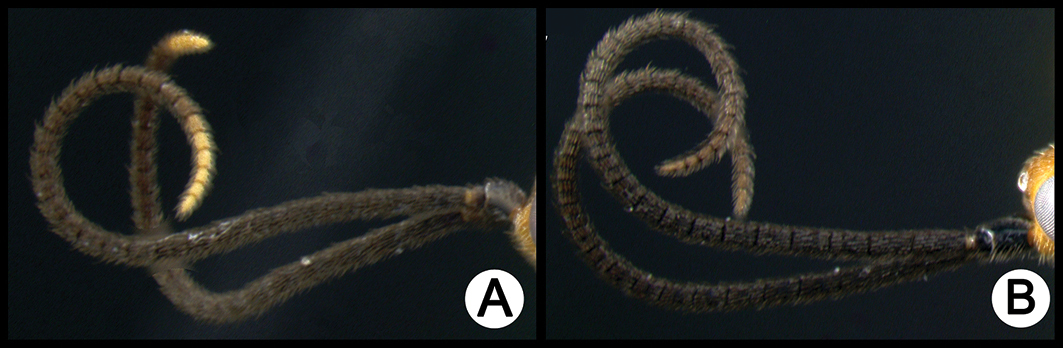	
22(21)	A. Pronotum entirely pale (yellow to orange)	**23**
–	B. Pronotum bicolored	***L. pablocobbi***
	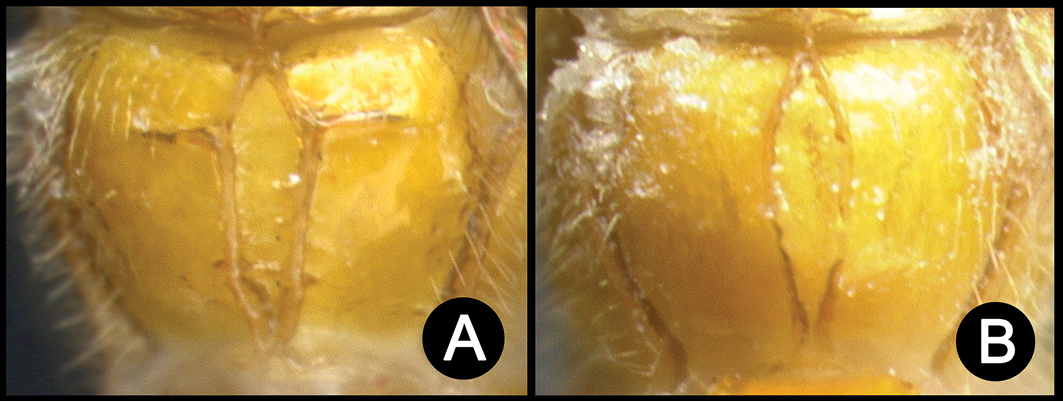	
23(22)	A. Median areola length 4× its width; median areola of propodeum kite-shaped	***L. motohasegawai*** ♀
–	B. Median areola length 15× its width; median areola of propodeum spindle-shaped	***L. gisukae***
	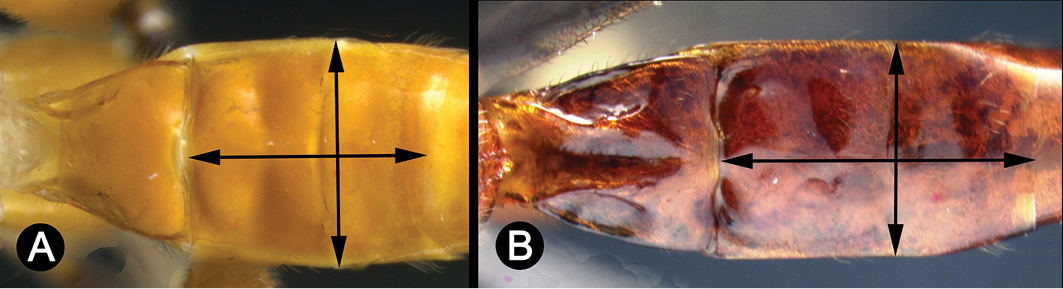	
24(21)	A. Apical flagellomeres usually bright yellow, always distinctly paler than subapical flagellomeres	***L. chrysokeras***
–	B. Apical flagellomeres brown not distinctly paler than subapical flagellomeres	**25**
	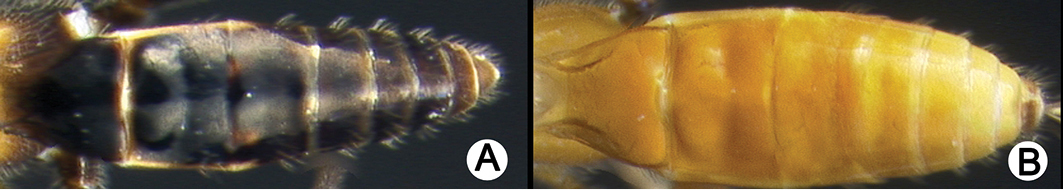	
25(24)	A. Median areola of propodeum kite-shaped	***L. miguelviquezi*** ♀
–	B. Median areola of propodeum spindle-shaped	**26**
	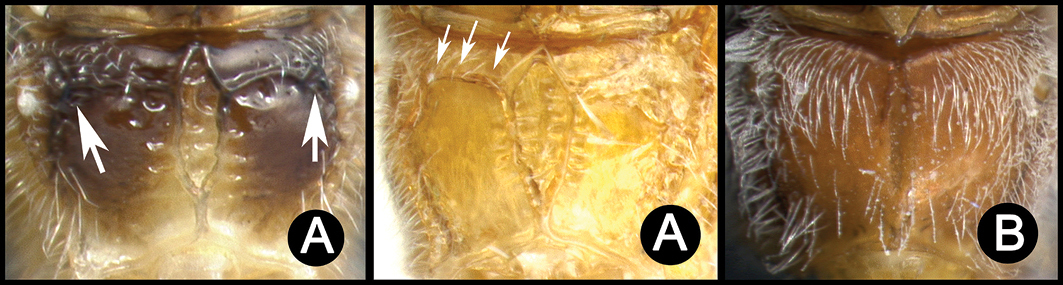	
26(25)	A. Vertex of head entirely melanic	**27**
–	B. Vertex of head mostly or entirely yellow	***L. gustavoindunii***
	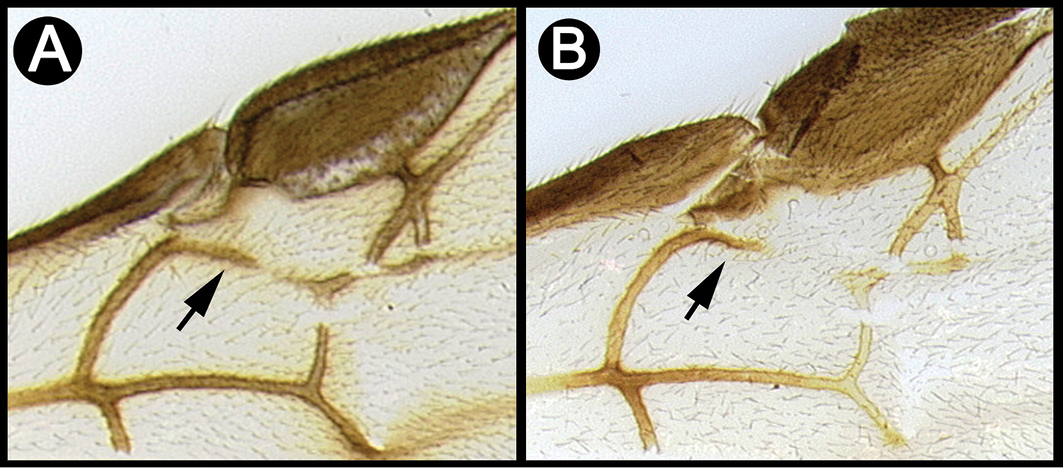	
27(26)	A. Median syntergite 2+3 1.1 times longer than wide	***L. alfredomainieri***
–	B. Median syntergite 2+3 1.5 times longer than wide	***L. okchunae***
	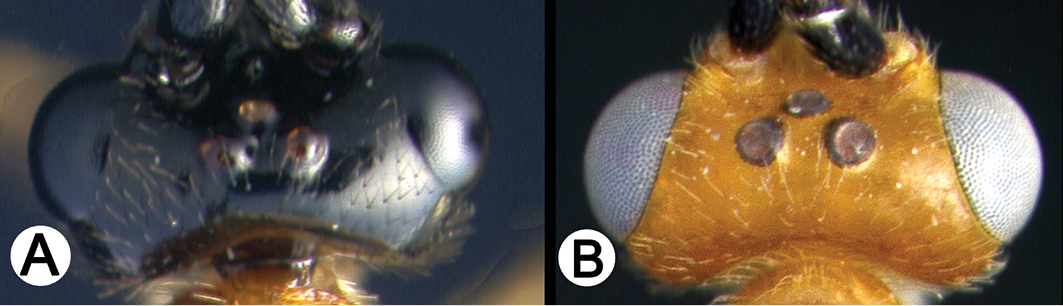	
28(1)	A. Median tergites mostly or entirely melanic (brown to black)	***L. cesarmorai***
–	B. Median tergites entirely pale (yellow to orange) or mostly pale with posterior terga black	**29**
	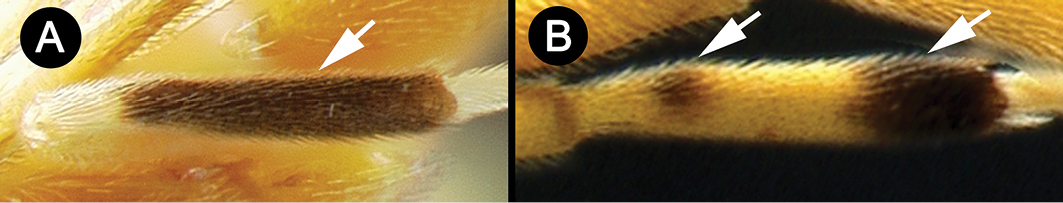	
29(28)	A. Anterior transverse carinae of propodeum reaching the lateral margin; median areola of propodeum kite-shaped	**30**
–	B. Anterior transverse carinae of propodeum not reaching the lateral margin or absent; median areola of propodeum spindle-shaped	***L. ivanniasandovalae***
	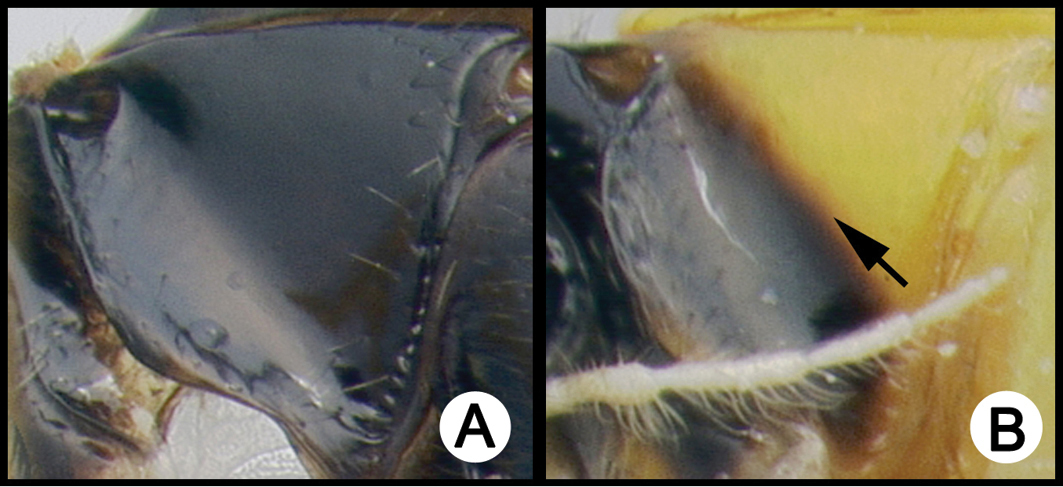	
30(29)	A. Fore wing RS+Ma tubular on more than half its length	***L. sergiobermudezi***
–	B. Fore wing RS+Ma tubular on less than one third its length	***L. mariamartachavarriae***
	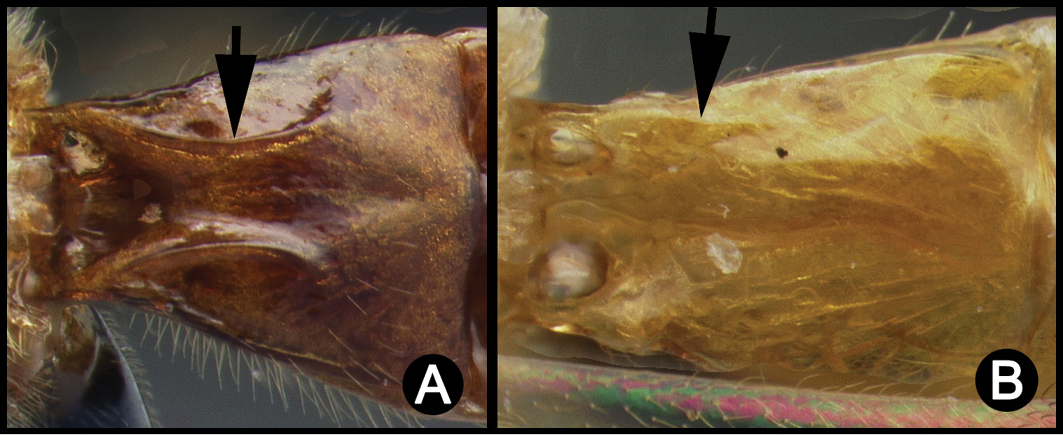	
31(1)	A. Vertex of head mostly or entirely melanic	***L. youngcheae***
–	B. Vertex of head mostly or entirely yellow	**32**
	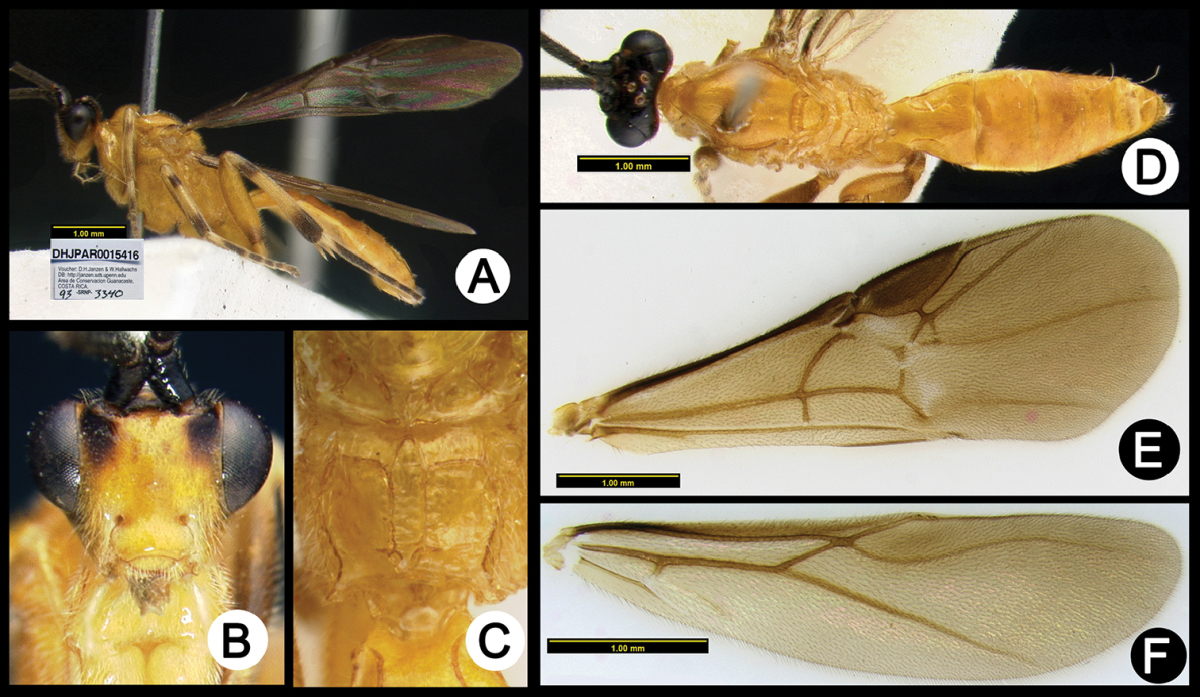	
32(31)	A. Mid tibia mostly black, yellow basally	***L. eddysanchezi***
–	B. Mid tibia with a sub-basal black patch and black apically, yellow at mid-length and basally	***L. hartmanguidoi***
	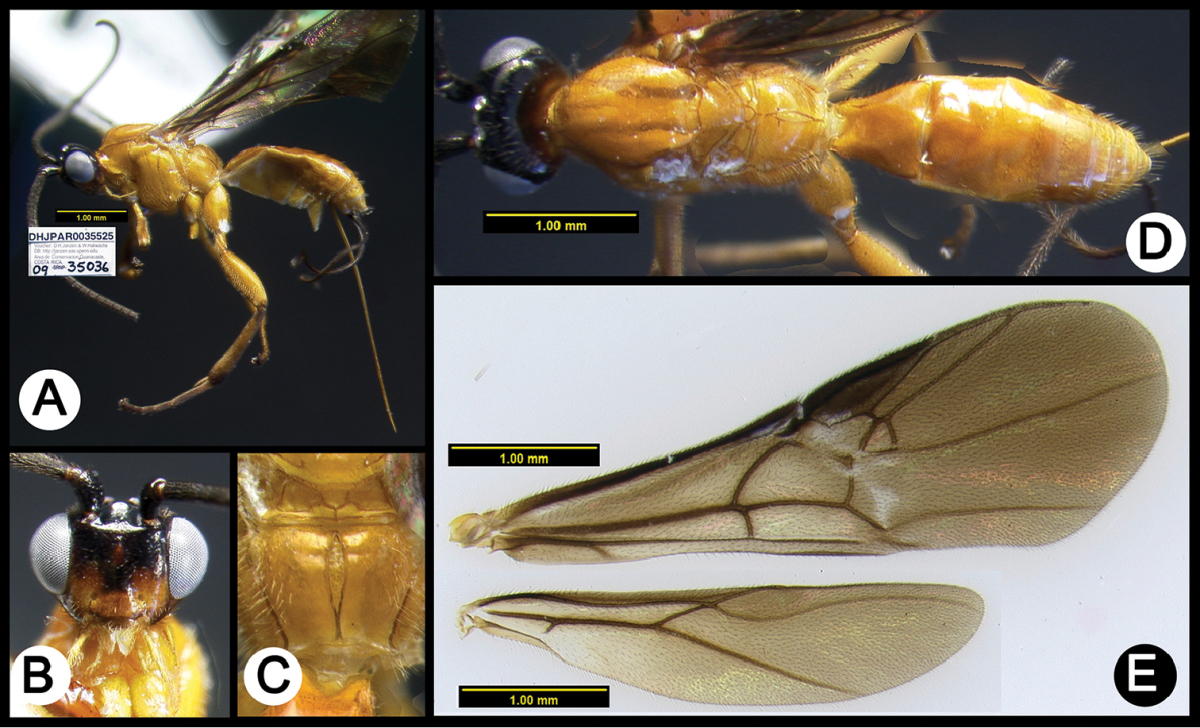	
33(1)	A. Pronotum entirely melanic	**34**
–	B. Pronotum bicolored	***L. ericchapmani***
	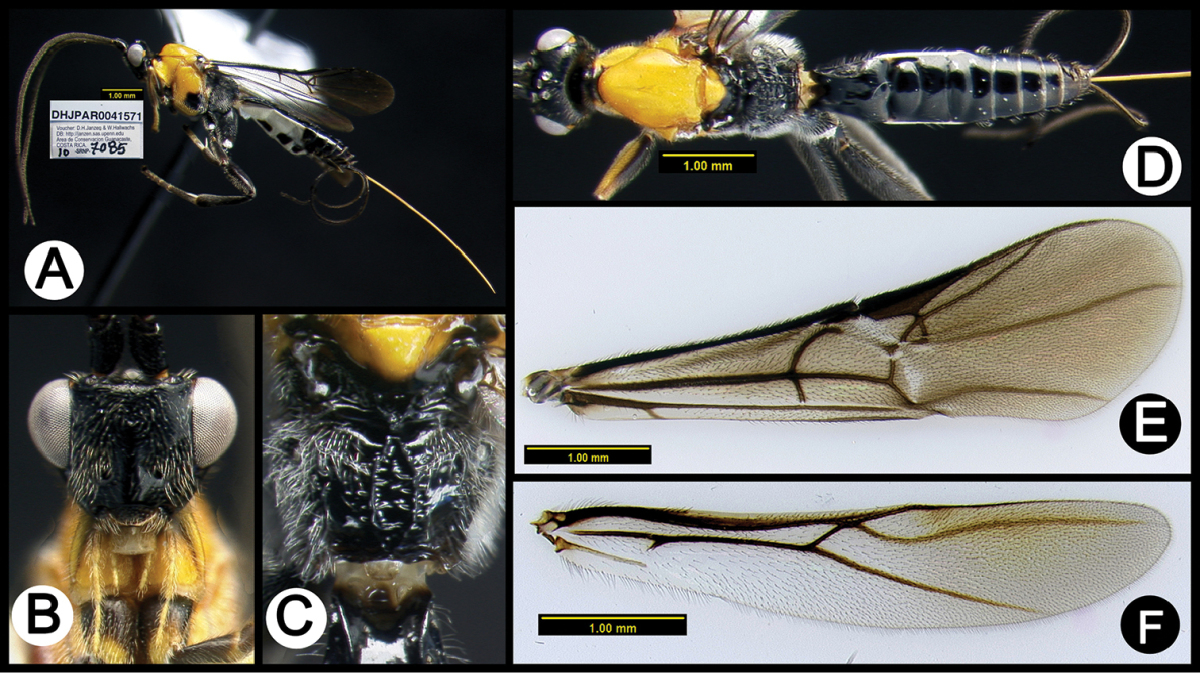	
34(33)	A. Lateral longitudinal carinae of median tergite 1 well-defined	***L. hokwoni***
–	B. Lateral longitudinal carinae of median tergite 1 blunt.	***L. robertofernandezi***
	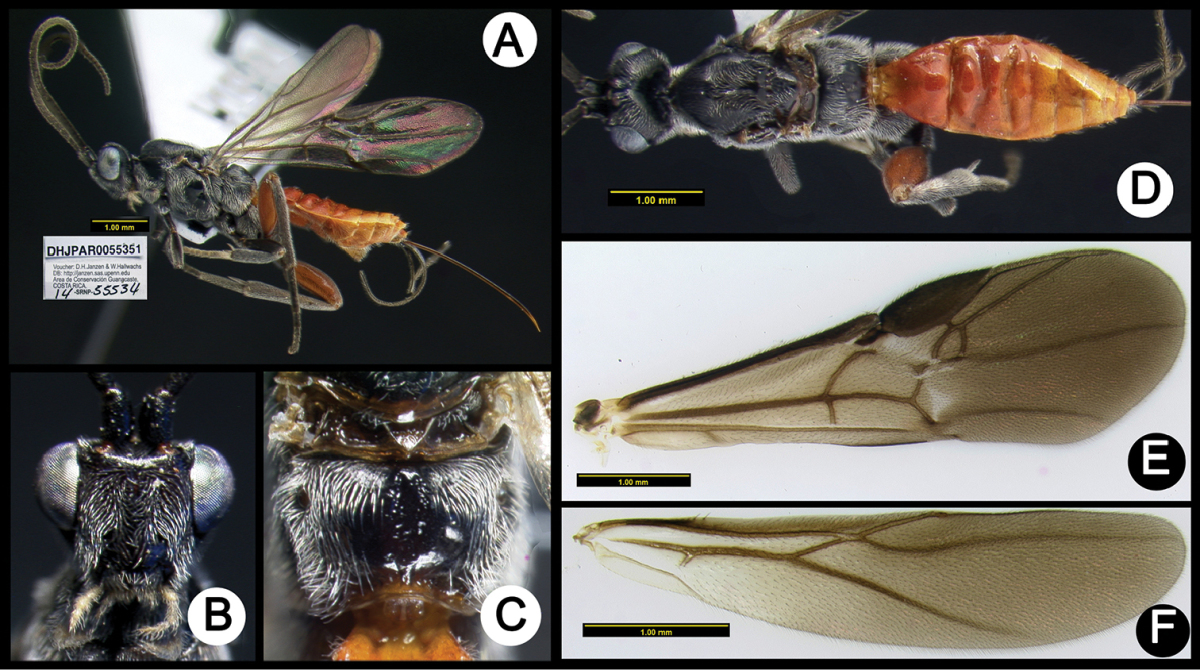	

### Species descriptions

#### Lytopylus alejandromasisi

Taxon classificationAnimaliaHymenopteraBraconidae

Kang sp. n.

http://zoobank.org/C27B1AC1-2924-4700-AB21-134B9882E3F2

Fig. 3

##### Diagnosis.

Fore wing mostly infuscated; hind tibia black basally and distally, yellow at mid-length; pronotum entirely yellow; mesoscutum entirely pale; anterior transverse carina of propodeum reaching the lateral margin; median tergites entirely pale.

##### Description.

Holotype: male. Body length 5.6 mm. Fore wing length 5.4 mm. Fore wing mostly infuscated. Scutellar sulcus with five longitudinal carinae. Median areola of propodeum with well-defined margins. Anterior transverse carina of propodeum reaching the lateral margin. Lateral longitudinal carinae of median tergite 1 well-defined. Median syntergite 2+3 1.1 times longer than wide.

##### Female.

Unknown.

##### Etymology.

*Lytopylus alejandromasisi* is named in honor of Alejandro Masis in recognition of his participation in the collaborative development of the ICE-ACG geothermal project of Pailas II, northwestern Costa Rica.

##### Biology.

Reared one time from Gelechiidae “same as 93-SRNP-3345.1” feeding on very new leaves of *Bursera tomentosa* (Burseraceae) in ACG dry forest at 280 m elevation.

##### Type material.

Holotype ♂: Costa Rica, Guanacaste, Sector Santa Rosa, Quebrada Duende, Area de Conservaciόn Guanacaste 10.83663N -85.61144W 280m., gusaneros coll., host plant: Burseraceae *Bursera tomentosa*, host caterpillar: Gelechiidae, same as 93-SRNP-3345.1, coll. date: 7/7/1993, parasitoid eclosion date: 7/25/1993, DHJPAR0015416.

**Figure 3.**
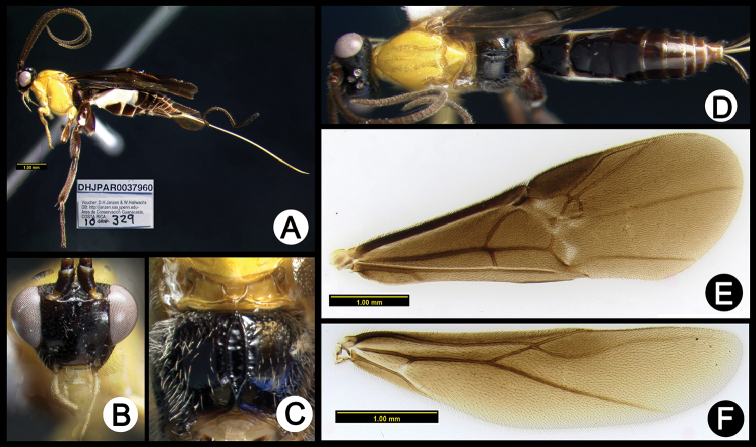
*Lytopylus alejandromasisi* holotype: **A** lateral habitus **B** anterior head **C** propodeum **D** dorsal habitus **E** fore wing **F** hind wing.

#### Lytopylus alfredomainieri

Taxon classificationAnimaliaHymenopteraBraconidae

Kang sp. n.

http://zoobank.org/ED76E996-F310-4605-BB8D-F1EC579B21D9

Fig. 4

##### Diagnosis.

Apical flagellomeres brown not distinctly paler than subapical flagellomeres; vertex of head entirely melanic; fore wing mostly infuscated; pronotum entirely pale (yellow to orange); fore tibia entirely pale (yellow to orange); mesoscutum entirely pale; anterior transverse carina of propodeum not reaching the lateral margin; median tergites entirely pale (yellow to orange); median syntergite 2+3 1.1 times longer than wide.

##### Description.

Holotype: female. Body length 4.4 mm. Fore wing length 5.1 mm. Fore wing mostly infuscated. Pronotum entirely pale (yellow to orange). Scutellar sulcus with one median longitudinal carina. Median areola of propodeum with well-defined margins. Anterior transverse carina of propodeum not reaching the lateral margin. Lateral longitudinal carinae of median tergite 1 well-defined. Median syntergite 2+3 1.1 times longer than wide. Ovipositor about same length as body.

##### Males.

Similar to holotype except for face. Face usually paler than holotype.

##### Etymology.

*Lytopylus alfredomainieri* is named in honor of Alfredo Mainieri in recognition of his participation in the collaborative development of the ICE-ACG geothermal project of Pailas II, northwestern Costa Rica.

##### Biology.

Reared five times from two species of *Olethreutes* (Olethreutinae, Tortricidae) leaf-tiers feeding on mature leaves of *Meliosma glabrata* (Sabiaceae) in ACG cloud forest edge at 1220 to 1276 m elevation.

##### Type material.

Holotype ♀: Costa Rica, Guanacaste, Sector Cacao, Sendero Derrumbe, Area de Conservación Guanacaste 10.92918N -85.46426W 1220m., Manuel Pereira coll., food plant: Sabiaceae *Meliosma glabrata*, host caterpillar: Tortricidae, Olethreutinae, *Olethreutes* Janzen188, coll. date: 2/2/2009, parasitoid eclosion date: 3/19/2009, DHJPAR0035525. Paratypes: [the following have the same data as the holotype except as indicated] ♂, DHJPAR0035519. ♀, DHJPAR0035513. ♂, Sector Pailas Gemelos, 10.76928N -85.34662W, 1276m., Jose Cortez coll., host caterpillar: *Olethreutes* Brown22, coll. date: 10/7/2009, parasitoid eclosion date: 11/2/2009, DHJPAR0038251. [same as previous except eclosion date] ♂, parasitoid eclosion date: 11/1/2009, DHJPAR0037861.

**Figure 4.**
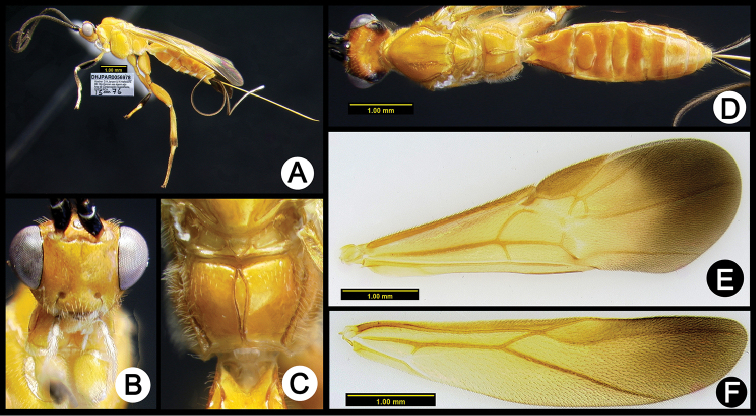
*Lytopylus alfredomainieri* holotype: **A** lateral habitus **B** anterior head **C** propodeum **D** dorsal habitus **E** wings.

#### Lytopylus anamariamongeae

Taxon classificationAnimaliaHymenopteraBraconidae

Kang sp. n.

http://zoobank.org/14F4C7B3-3857-4584-8CCA-3781096E338C

Fig. 5

##### Diagnosis.

Fore wing mostly infuscated; pronotum mostly pale, anteriorly black; mesoscutum entirely pale (yellow to orange); mesopleuron mostly pale, posteroventrally black; scutellar sulcus lacking longitudinal carina; median tergites entirely melanic.

##### Description.

Holotype: female. Body length 6.2 mm. Fore wing length 5.5 mm. Fore wing mostly infuscated. Scutellar sulcus lacking longitudinal carina. Median areola of propodeum with well-defined margins. Anterior transverse carina of propodeum reaching the lateral margin. Lateral longitudinal carinae of median tergite 1 well-defined. Median syntergite 2+3 1.5 times longer than wide. Ovipositor about same length as body.

##### Male.

Unknown.

##### Etymology.

*Lytopylus anamariamongeae* is named in honor of Ana Maria Monge in recognition of her participation in the collaborative development of the ICE-ACG geothermal project of Pailas II, northwestern Costa Rica.

##### Biology.

Reared one time from *Antaeocerconota* Janzen433 (Depressariidae) a leaf-tier feeding on mature leaves of *Inga punctata* (Fabaceae) in ACG dry forest – rain forest ecotone at 540 m elevation.

##### Type material.

Holotype ♀: Costa Rica, Alajuela, Sector San Cristobal, Tajo Angeles, Area de Conservaciόn Guanacaste 10.86472N -85.41531W 540m., Gloria Sihezar coll., food plant: Fabaceae *Inga punctata*, host caterpillar: Depressariidae, Stenomatinae, *Antaeocerconota* Janzen433, coll. date: 11/29/2010, parasitoid eclosion date: 12/17/2010, DHJPAR0041571.

**Figure 5.**
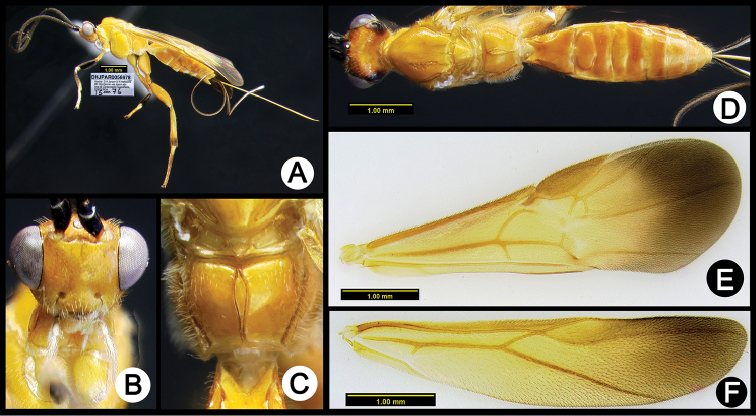
*Lytopylus anamariamongeae* holotype: **A** lateral habitus **B** anterior head **C** propodeum **D** dorsal habitus **E** fore wing **F** hind wing.

#### Lytopylus angelagonzalezae

Taxon classificationAnimaliaHymenopteraBraconidae

Kang sp. n.

http://zoobank.org/53BD9379-0092-4255-A872-311C8795407F

Fig. 6

##### Diagnosis.

Fore wing mostly infuscated; mesoscutum entirely melanic; median areola of propodeum lacking well-defined margins; anterior transverse carina of propodeum absent; median tergites entirely reddish orange; lateral longitudinal carinae of median tergite 1 blunt.

##### Description.

Holotype: female. Body length 5.8 mm. Fore wing length 5.6 mm. Fore wing mostly infuscated. Scutellar sulcus with three longitudinal carinae. Median areola of propodeum lacking well-defined margins. Anterior transverse carina of propodeum absent. Lateral longitudinal carinae of median tergite 1 blunt. Median syntergite 2+3 as long as wide. Ovipositor length longer than metasoma, but shorter than body.

##### Male.

Unknown.

##### Etymology.

*Lytopylus angelagonzalezae* is named in honor of Angela González Grau in recognition of her participation in the collaborative development of the ICE-ACG geothermal project of Pailas II, northwestern Costa Rica.

##### Biology.

Reared two times from *Anacampsis* Janzen353 (Anacampsinae, Gelechiidae) feeding on two species of Rutaceae in ACG dry forest – rain forest ecotone at 280 to 825 m elevation.

##### Type material.

Holotype ♀: Costa Rica, Guanacaste, Sector Pailas, PL12, Area de Conservaciόn Guanacaste 10.76248N -85.33689W 752m., Jose Cortez coll., food plant: Rutaceae *Zanthoxylum acuminatum*, host caterpillar: Gelechiidae, Anacampsinae, *Anacampsis* Janzen353, coll. date: 3/6/2014, parasitoid eclosion date: 4/9/2014, DHJPAR0055351.Paratype: [the following have the same data as the holotype except as indicated] ♀, Sector Del Oro, Puente Mena, 11.04562N -85.45742W 280m., Lucia Ríos coll., food plant: *Amyris pinnata*, coll. date: 4/1/2002, parasitoid eclosion date: 4/15/2002, DHJPAR0015430.

**Figure 6.**
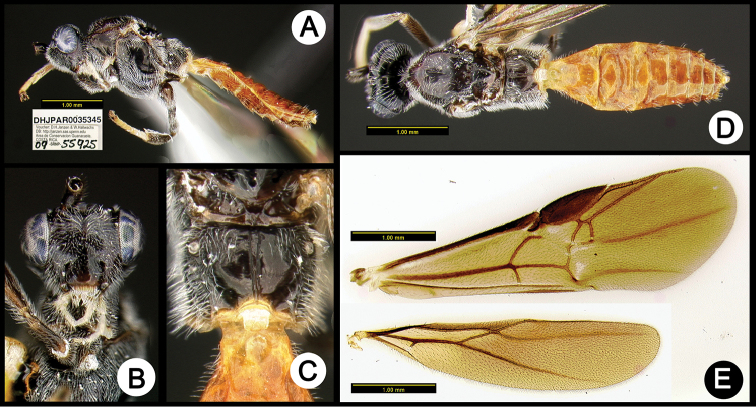
*Lytopylus angelagonzalezae* holotype: **A** lateral habitus **B** anterior head **C** propodeum **D** dorsal habitus **E** fore wing **F** hind wing.

#### Lytopylus cesarmorai

Taxon classificationAnimaliaHymenopteraBraconidae

Kang sp. n.

http://zoobank.org/5D2E689D-6076-4242-938B-73C772324EE1

Fig. 7

##### Diagnosis.

Fore wing mostly infuscated; scutellar sulcus with one median longitudinal carina; median tergites entirely melanic; lateral tergites one and two entirely white.

##### Description.

Holotype: female. Body length 5.7 mm. Fore wing length 5.4 mm. Fore wing mostly infuscated. Scutellar sulcus with one median longitudinal carina. Median areola of propodeum with well-defined margins. Anterior transverse carina of propodeum absent. Lateral longitudinal carinae of median tergite 1 well-defined. Median syntergite 2+3 1.5 times longer than wide. Ovipositor slightly longer than body.

##### Male.

Fore wing with a slight yellow tinge. Body color pattern similar to holotype, but slightly lighter.

##### Etymology.

*Lytopylus cesarmorai* is named in honor of Cesar Mora in recognition of his participation in the collaborative development of the ICE-ACG geothermal project of Pailas II, northwestern Costa Rica.

##### Biology.

Reared two times from *Stenoma* BioLep82 (Depressariidae) feeding on mature leaves of *Apeiba membranacea* (Malvaceae) in ACG rain forest at 527 m elevation.

##### Type material.

Holotype ♀: Costa Rica, Alajuela, Sector San Cristobal, Sendero Huerta, Area de Conservaciόn Guanacaste 10.9305N -85.37223W 527m., Carolina Cano coll., food plant: Malvaceae *Apeiba membranacea*, host caterpillar: Depressariidae, Stenomatinae, *Stenoma* BioLep82, coll. date: 1/11/2010, parasitoid eclosion date: 2/5/2010, DHJPAR0037960. Paratype: [the following have the same data as the holotype except as indicated] ♂, parasitoid eclosion date: 2/9/2010, DHJPAR0038304.

**Figure 7.**
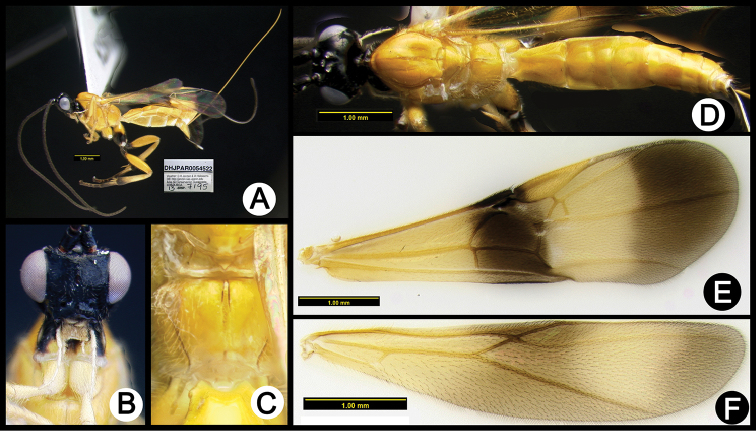
*Lytopylus cesarmorai* holotype: **A** lateral habitus **B** anterior head **C** propodeum **D** dorsal habitus **E** fore wing **F** hind wing.

#### Lytopylus chrysokeras

Taxon classificationAnimaliaHymenopteraBraconidae

(Sharkey)

Fig. 8

Austroearinus chrysokeras (Sharkey), (Sharkey 2006).
Lytopylus chrysokeras Sharkey, (Sharkey et al. 2016).

##### Diagnosis.

Apical flagellomeres usually bright yellow, always distinctly paler than subapical flagellomeres; fore wing mostly infuscated; hind tibia black apically; mesoscutum entirely pale; median tergites entirely pale (yellow to orange).

##### Biology.

Reared 48 times from seven species of dichomeridine Gelechiidae feeding on seven species of mature leaves of Malvaceae, Violaceae, Rubiaceae, Asteraceae, and Fabaceae growing in ACG rain forest at 240 to 645 m elevation.

##### Material.

♀: Costa Rica, Alajuela, Sector San Cristobal, Tajo Angeles, Area de Conservaciόn Guanacaste 10.86472N -85.41531W 540m., Carolina Cano coll., food plant: Violaceae *Rinorea squamata*, host caterpillar: Gelechiidae, Dichomeridinae, gelJanzen01 Janzen485, coll. date: 6/10/2010, parasitoid eclosion date: 6/29/2010, DHJPAR0040492. [the following have the same data as previous except as indicated] ♂, parasitoid eclosion date: 6/26/2010, DHJPAR0040344. ♂, parasitoid eclosion date: 6/27/2010, DHJPAR0040476. ♂, parasitoid eclosion date: 6/30/2010, DHJPAR0040485. ♂, parasitoid eclosion date: 6/27/2010, DHJPAR0040487. ♀, Gloria Sihezar coll., coll. date: 6/15/2010, parasitoid eclosion date: 7/2/2010, DHJPAR0040473. ♀, Gloria Sihezar coll. food plant: Malvaceae *Mortoniodendron costaricense*, host caterpillar: gelJanzen01 Janzen356, coll. date: 7/8/2010, parasitoid eclosion date: 7/25/2010, DHJPAR0040505. ♀, Elda Araya coll., food plant: Malvaceae *Mortoniodendron costaricense*, host caterpillar: gelJanzen01 Janzen356, coll. date: 7/8/2010, parasitoid eclosion date: 7/23/2010, DHJPAR0040460. ♀, Osvaldo Espinoza coll., food plant: *Rinorea deflexiflora*, host caterpillar: gelJanzen01 Janzen179, coll. date: 6/4/2014, parasitoid eclosion date: 6/20/2014, DHJPAR0055352. ♂, Finca San Gabriel, 10.87766N -85.39343W 645m., Carolina Cano coll., food plant: Malvaceae *Mortoniodendron costaricense*, host caterpillar: gelJanzen01 Janzen356, coll. date: 11/16/2012, parasitoid eclosion date: 12/5/2012, DHJPAR0051373. [same as previous except coll. date and eclosion date] ♂, coll. date: 11/30/2012, parasitoid eclosion date: 12/21/2012, DHJPAR0051162. ♀, Gloria Sihezar coll., coll. date: 6/2/2012, parasitoid eclosion date: 6/28/2012, DHJPAR0049276. ♀, Elda Araya coll., coll. date: 10/11/2012, parasitoid eclosion date: 11/4/2012, DHJPAR0051364. [the following have the same data as the holotype except as indicated] ♂, Cementerio Viejo, 10.88111N -85.38889W 570m., Gloria Sihezar coll., food plant: Malvaceae *Mortoniodendron costaricense*, host caterpillar: gelJanzen01 Janzen356, coll. date: 7/30/2014, parasitoid eclosion date: 8/17/2014, DHJPAR0057739. ♂, Rio Blanco Abajo, 10.90037N -85.37254W 500m., food plant: Malvaceae *Mortoniodendron costaricense*, host caterpillar: gelJanzen01 Janzen356, coll. date: 6/29/2009, parasitoid eclosion date: 7/19/2009, DHJPAR0040070. ♀, Sector Rincon Rain Forest, Rio Francia Arriba, 10.89666N -85.29003W 400m., Jose Perez coll., food plant: Fabaceae *Dialium guianense*, host caterpillar: *Dichomeris* Janzen512, coll. date: 1/19/2011, parasitoid eclosion date: 2/7/2011, DHJPAR0042837. [same as previous except and eclosion date] ♀, parasitoid eclosion date: 2/10/2011, DHJPAR0042835. ♀, parasitoid eclosion date: 2/9/2011, DHJPAR0043002. ♀, Sector Rincon Rain Forest, Sendero Anonas, 10.90528N -85.27882W 405m., Jose Perez coll., food plant: Violaceae *Rinorea deflexiflora*, coll. date: 4/6/2012, parasitoid eclosion date: 5/1/2012, DHJPAR0049388. [same as previous except as indicated] ♂, food plant: *Rinorea hummelii*, coll. date: 2/12/2013, parasitoid eclosion date: 2/26/2013, DHJPAR0051817. ♀, Anabelle Cordoba coll., food plant: *Rinorea hummelii*, coll. date: 5/20/2014, parasitoid eclosion date: 6/6/2014, DHJPAR0055505. ♂, Anabelle Cordoba coll., coll. date: 5/20/2014, parasitoid eclosion date: 6/5/2014, DHJPAR0055528. [same as previous except as indicated] ♀, parasitoid eclosion date: 6/8/2014, DHJPAR0055522. ♂, parasitoid eclosion date: 6/7/2014, DHJPAR0055527. ♀, parasitoid eclosion date: 6/11/2014, DHJPAR0055353. ♂, coll. date: 5/23/2014, parasitoid eclosion date: 6/7/2014, DHJPAR0055543. ♂, coll. date: 5/23/2014, parasitoid eclosion date: 6/9/2014, DHJPAR0055490. ♂, coll. date: 5/23/2014, parasitoid eclosion date: 6/7/2014, DHJPAR0055507. ♀, coll. date: 5/30/2014, parasitoid eclosion date: 6/18/2014, DHJPAR0055513. ♀, coll. date: 6/2/2014, parasitoid eclosion date: 6/14/2014, DHJPAR0055504. ♀, Sector Rincon Rain Forest, Jacobo, 10.94076N -85.3177W 461m., Petrona Rios coll., food plant: *Rinorea deflexiflora*, host caterpillar: gelJanzen01 Janzen179, coll. date: 6/7/2014, parasitoid eclosion date: 6/23/2014, DHJPAR0055986. ♂, Sector Rincon Rain Forest, Conguera, 10.91589N -85.26631W 420m., Jose Perez coll., food plant: Asteraceae *Koanophyllon hylonoma*, host caterpillar: *Dichomeris* Janzen703, coll. date: 7/1/2009, parasitoid eclosion date: 7/22/2009, DHJPAR0040067. ♂, Guanacaste, Sector Pitilla, Ingas, 11.00311N -85.42041W 580m., Natalia Santamaria coll. food plant: *Rinorea deflexiflora*, host caterpillar: gelJanzen01 Janzen781, coll. date: 5/29/2004, parasitoid eclosion date: 6/21/2004, DHJPAR0015527. ♂, Guanacaste, Sector Pitilla, Sendero Trichoptera, 10.98571N -85.41869W 655m., Calixto Moraga coll. food plant: *Rinorea sylvatica*, coll. date: 10/8/2009, parasitoid eclosion date: 10/27/2009, DHJPAR0040065. ♂, Guanacaste, Sector Pitilla, Sendero Laguna, 10.9888N -85.42336W 680m., Roster Moraga coll., food plant: Rubiaceae *Chiococca alba*, coll. date: 1/8/2010, parasitoid eclosion date: 1/28/2010, DHJPAR0038356. ♂, Guanacaste, Sector Pitilla, Leonel, 10.99637N -85.40195W 510m., Dinia Martinez coll., food plant: *Rinorea sylvatica*, coll. date: 2/4/2010, parasitoid eclosion date: 2/22/2010, DHJPAR0039071. ♀, Guanacaste, Sector Pitilla, Sendero Orosilito, 10.98332N -85.43623W 900m., Freddy Quesada coll., food plant: Malpighiaceae *Hiraea reclinata*, host caterpillar: gelJanzen01 Janzen19, coll. date: 7/25/2013, parasitoid eclosion date: 8/15/2013, DHJPAR0052673. ♂, Guanacaste, Sector Del Oro, Quebrada Raiz, 11.02865N -85.48669W 280m., Elieth Cantillano coll. food plant: *Rinorea deflexiflora*, coll. date: 5/18/2010, parasitoid eclosion date: 6/3/2010, DHJPAR0040331. [same as previous except collector and eclosion date] ♂, Roster Moraga coll., parasitoid eclosion date: 6/1/2010, DHJPAR0040335. ♂, Roster Moraga coll., parasitoid eclosion date: 6/2/2010, DHJPAR0040503.

**Figure 8.**
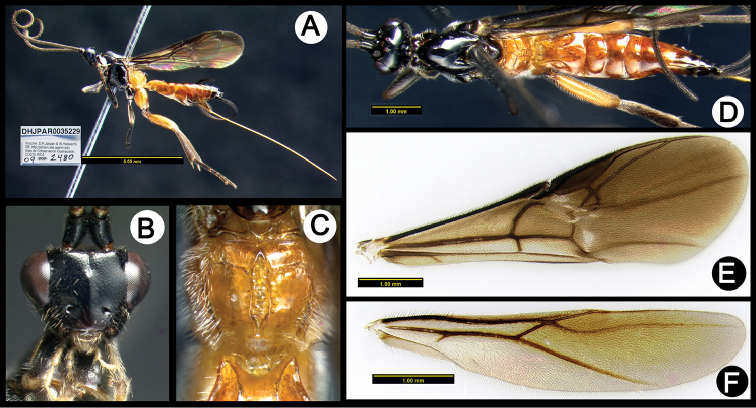
*Lytopylus chrysokeras*: **A** lateral habitus **B** anterior head **C** propodeum **D** dorsal habitus **E** fore wing **F** hind wing.

#### Lytopylus eddysanchezi

Taxon classificationAnimaliaHymenopteraBraconidae

Kang sp. n.

http://zoobank.org/7BD05EB2-A120-4442-AABA-0D70E4AE0FB6

Fig. 9

##### Diagnosis.

Vertex of head entirely yellow; fore wing with one black band; mid tibia mostly melanic, yellow basally.

##### Description.

Holotype: female. Body length 6.0 mm. Fore wing length 5.5 mm. Fore wing with one black band. Scutellar sulcus with one median longitudinal carina. Median areola of propodeum with well-defined margins. Anterior transverse carina of propodeum absent. Lateral longitudinal carinae of median tergite 1 well-defined. Median syntergite 2+3 1.2 times longer than wide. Ovipositor about same length as body.

##### Males.

Similar to holotype except for fore legs color. Fore legs usually less melanic.

##### Etymology.

*Lytopylus eddysanchezi* is named in honor of Eddy Sánchez in recognition of his participation in the collaborative development of the ICE-ACG geothermal project of Pailas II, northwestern Costa Rica.

##### Biology.

Reared 11 times from one species leaf-tier in the Depressariidae, feeding on mature leaves of *Meliosma glabrata* (Sabiaceae) in ACG rain forest at 540 to 645 m elevation.

##### Type material.

Holotype ♀: Costa Rica, Alajuela, Sector San Cristobal, Finca San Gabriel, Area de Conservaciόn Guanacaste 10.87766N -85.39343W 645m., Gloria Sihezar coll., food plant: Sabiaceae *Meliosma glabrata*, host caterpillar: Depressariidae, subfamily unknown, elachJanzen01 Janzen900, coll. date: 1/7/2015, parasitoid eclosion date: 2/7/2015, DHJPAR0056978. Paratypes: [the following have the same data as the holotype except as indicated] ♂, Sendero Corredor, 10.87868N -85.38963W 620m., Elda Araya coll., coll. date: 11/7/2009, parasitoid eclosion date: 12/5/2009, DHJPAR0038240. ♂, Sendero Corredor, 10.87868N -85.38963W 620m., coll. date: 7/25/2013, parasitoid eclosion date: 8/17/2013, DHJPAR0052903. ♀, Tajo Angeles, 10.86472N -85.41531W 540m., coll. date: 9/20/2010, parasitoid eclosion date: 10/14/2010, DHJPAR0041600. ♂, Sendero Carmona, 10.87621N -85.38632W 670m., coll. date: 3/25/2012, parasitoid eclosion date: 4/18/2012, DHJPAR0049278. ♀, coll. date: 2/24/2014, parasitoid eclosion date: 3/31/2014, DHJPAR0055232. ♂, Elda Araya coll., coll. date: 11/26/2009, parasitoid eclosion date: 12/22/2009, DHJPAR0038208. [same as previous except as coll. date and eclosion date] ♀, coll. date: 11/30/2009, parasitoid eclosion date: 1/1/2010, DHJPAR0038180. ♂, coll. date: 11/30/2009, parasitoid eclosion date: 12/23/2009, DHJPAR0038231. ♂, coll. date: 11/30/2009, parasitoid eclosion date: 12/21/2009, DHJPAR0038225. ♂, coll. date: 12/16/2012, parasitoid eclosion date: 1/13/2013, DHJPAR0051357.

**Figure 9.**
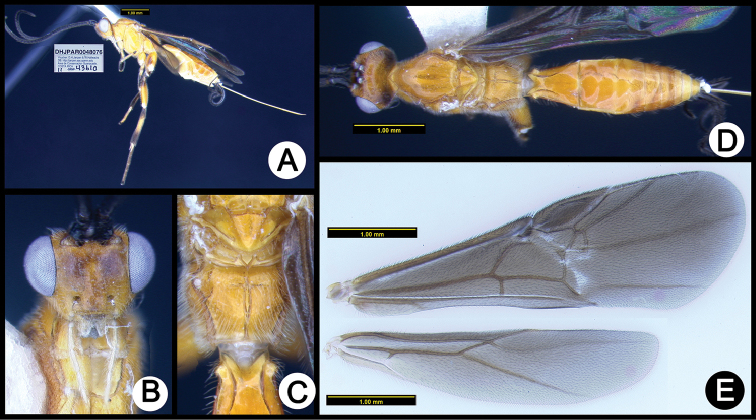
*Lytopylus eddysanchezi* holotype: **A** lateral habitus **B** anterior head **C** propodeum **D** dorsal habitus **E** fore wing **F** hind wing.

#### Lytopylus eliethcantillanoae

Taxon classificationAnimaliaHymenopteraBraconidae

Kang sp. n.

http://zoobank.org/747FAD7E-4637-4F8E-8E35-93BCFD8D5739

Fig. 10

##### Diagnosis.

Fore wing mostly infuscated; mesoscutum entirely melanic; anterior transverse carina of propodeum absent; median areola of propodeum narrow and not closed posteriorly; median areola length 11x its width, lateral longitudinal carinae of median tergite 1 well-defined; median tergites entirely reddish orange.

##### Description.

Holotype: male. Body length 5.2 mm. Fore wing length 5.3 mm. Fore wing mostly infuscated. Scutellar sulcus with three longitudinal carinae. Median areola of propodeum with well-defined margins. Anterior transverse carina of propodeum absent. Lateral longitudinal carinae of median tergite 1 well-defined. Median syntergite 2+3 1.1 times longer than wide. Ovipositor longer than metasoma, but shorter than body.

##### Female.

Unknown.

##### Etymology.

*Lytopylus eliethcantillanoae* is named in honor of Elieth Cantillano in recognition of her participation in the collaborative development of the ICE-ACG geothermal project of Pailas II, northwestern Costa Rica.

##### Biology.

Reared perhaps one time from elachJanzen01 Janzen873 (Depressariidae) feeding on *Malvaviscus arboreus* (Malvaceae) in ACG dry forest – rain forest ecotone at 840 m elevation.

##### Type material.

Holotype ♂: Costa Rica, Guanacaste, Sector Santa Maria, Estacion Santa Maria, Area de Conservaciόn Guanacaste 10.76448N -85.31161W 840m., Jose Cortez coll., food plant: Malvaceae *Malvaviscus arboreus*, host caterpillar: Depressariidae, subfamily unknown, elachJanzen01 Janzen873, coll date: 4/26/2009, parasitoid eclosion date: 5/12/2009, DHJPAR0035345.

**Figure 10.**
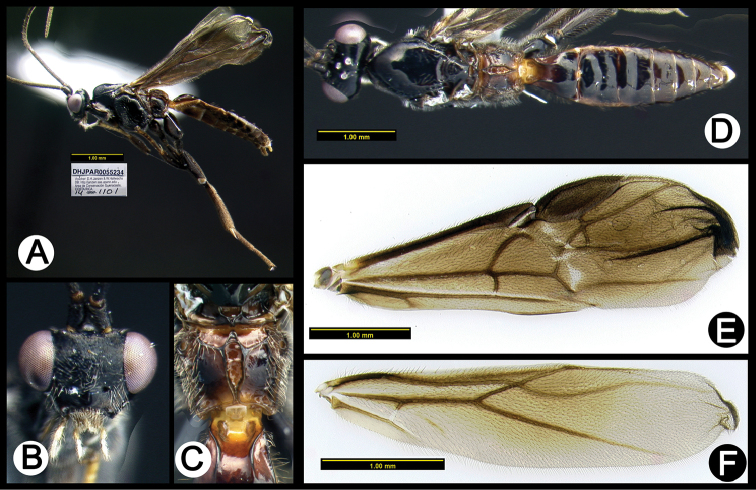
*Lytopylus eliethcantillanoae* holotype: **A** lateral habitus **B** anterior head **C** propodeum **D** dorsal habitus **E** wings.

#### Lytopylus ericchapmani

Taxon classificationAnimaliaHymenopteraBraconidae

Kang sp. n.

http://zoobank.org/A2C8DCE5-4DA5-4F79-94A2-1D7750C5AFDA

Fig. 11

##### Diagnosis.

Fore wing with two black bands; pronotum anteriorly black and posteriorly pale.

##### Description.

Holotype: female. Body length 5.8 mm. Fore wing length 5.7 mm. Fore wing with two black bands. Scutellar sulcus lacking longitudinal carina. Anterior transverse carina of propodeum absent. Median areola of propodeum with well-defined margins. Median areola of propodeum narrow. Lateral longitudinal carinae of median tergite 1 well-defined. Median syntergite 2+3 1.7 times longer than wide. Ovipositor slightly longer than body.

##### Male.

Body color similar to holotype. Body length slightly shorter than holotype.

##### Etymology.

Named in honor of Dr Eric G. Chapman, research analyst in the Department of Entomology at the University of Kentucky, for his kindly advice on molecular systematics and phylogenetics.

##### Biology.

Reared five times but only from the leaf-tier *Stenoma adytodes* (Depressariidae) feeding on mature leaves of *Pouteria juruana* (Sapotaceae) at the intersection of the ACG dry forest and rain forest ecosystems at 722 m elevation.

##### Type material.

Holotype ♀: Costa Rica, Alajuela, Sector San Cristobal, Jardin Estrada, Area de Conservaciόn Guanacaste 10.86546N -85.39694W 722m., Carolina Cano coll., food plant: Sapotaceae *Pouteria juruana*, host caterpillar: Depressariidae, Stenomatinae, *Stenoma adytodes*, coll. date: 12/10/2013, parasitoid eclosion date: 1/12/2014, DHJPAR0054533. Paratypes: [the following have the same data as the holotype except as indicated] ♀, coll. date: 2/5/2013, parasitoid eclosion date: 1/4/2014, DHJPAR0054527. ♀, parasitoid eclosion date: 1/9/2014, DHJPAR0054522. ♀, parasitoid eclosion date: 1/7/2014, DHJPAR0055238. ♂, Elda Araya coll., coll. date: 12/5/2013, parasitoid eclosion date: 12/26/2013, DHJPAR0054526.

**Figure 11.**
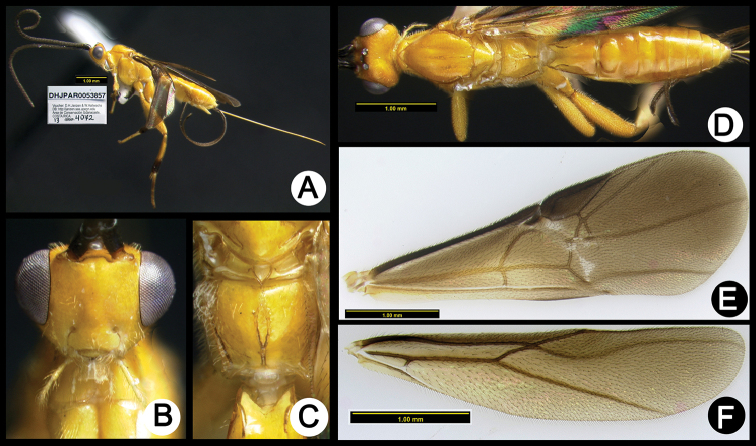
*Lytopylus ericchapmani* holotype: **A** lateral habitus **B** anterior head **C** propodeum **D** dorsal habitus **E** fore wing **F** hind wing.

#### Lytopylus gahyunae

Taxon classificationAnimaliaHymenopteraBraconidae

Kang sp. n.

http://zoobank.org/34C499A2-CEC2-4D38-8D16-342D152B15B8

Fig. 12

##### Diagnosis.

Fore wing mostly infuscated; hind coxa entirely pale; mesoscutum entirely melanic; scutellar sulcus with one median longitudinal carina; anterior transverse carina of propodeum reaching the lateral margin; median tergites mostly pale with posterior terga black; median syntergite 2+3 1.4 times longer than wide.

##### Description.

Holotype: female. Body length 7.1 mm. Fore wing length 6.4 mm. Fore wing mostly infuscated. Scutellar sulcus with one median longitudinal carina. Median areola of propodeum with well-defined margins. Anterior transverse carina of propodeum reaching the lateral margin. Lateral longitudinal carinae of median tergite 1 well-defined. Median syntergite 2+3 1.4 times longer than wide. Ovipositor slightly longer than body.

##### Males.

Similar to holotype.

##### Etymology.

*Lytopylus gahyunae* is named in honor of Gahyun Park, wife of the first author.

##### Biology.

Reared 43 times from six species of *Antaeotricha* (40) and *Stenoma* (2) (Depressariidae) feeding on mature leaves of 3 species of *Guarea* and 1 of *Trichilia* (Meliaceae) in ACG rain forest at 380 to 620 m elevation.

##### Type material.

Holotype♀: Costa Rica, Alajuela, Sector San Cristobal, Rio Areno, Area de Conservaciόn Guanacaste 10.91407N -85.38174W 460m., Osvaldo Espinoza coll., food plant: Meliaceae *Guarea bullata*, host caterpillar: Depressariidae, Stenomatinae, *Antaeotricha* Janzen09, coll. date: 6/4/2009, parasitoid eclosion date: 6/26/2009, DHJPAR0035229. Paratypes: [the following have the same data as the holotype except as indicated] 2♀, coll. date: 6/4/2009, parasitoid eclosion date: 6/22/2009, DHJPAR0035298, DHJPAR0035294. ♀, parasitoid eclosion date: 6/23/2009, DHJPAR0036371. ♀, Elda Araya coll., food plant: *Guarea rhopalocarpa*, coll. date: 5/4/2009, parasitoid eclosion date: 6/21/2009, DHJPAR0035231. DHJPAR0035294. pes: [the following have the same data as the holotype except as indicated] 2odeum with well-defined ma8/27/2009, DHJPAR0036721. [same as previous except coll. date and eclosion date] ♀, coll. date: 9/13/2009, parasitoid eclosion date: 10/2/2009, DHJPAR0036686. ♂, Puente Palma, 10.9163N -85.37869W 460m., Carolina Cano coll., food plant: *Guarea kegelii*, coll. date: 11/10/2011, parasitoid eclosion date: 11/26/2011, DHJPAR0046956. ♀, Puente Palma, 10.9163N -85.37869W 460m., Gloria Sihezar coll., host caterpillar: *Antaeotricha* Janzen12, coll. date: 11/24/2012, parasitoid eclosion date: 12/27/2012, DHJPAR0051365. ♂, Tajo Angeles, 10.86472N -85.41531W 540m., Gloria Sihezar coll., food plant: *Guarea kegelii*, host caterpillar: Depressariinae, *Antaeotricha* Janzen07, coll. date: 9/24/2011, parasitoid eclosion date: 10/14/2011, DHJPAR0045788. [same as previous except coll. date and eclosion date] ♀, host caterpillar: Stenomatinae, coll. date: 11/6/2011, parasitoid eclosion date: 11/24/2011, DHJPAR0046745. ♀, host caterpillar: Stenomatinae, *Antaeotricha* Janzen09, coll. date: 11/22/2011, parasitoid eclosion date: 12/13/2011, DHJPAR0046744. ♀, Sendero Huerta, 10.9305N -85.37223W 527m., Gloria Sihezar coll., coll. date: 7/2/2012, parasitoid eclosion date: 7/27/2012, DHJPAR0049943. [same as previous except coll. date and eclosion date] ♀, 12/2/2012 12/24/2012, DHJPAR0051374. [same as previous except food plant, coll. date and eclosion date] 1♀, 1♂, food plant: *Guarea kegelii*, coll. date: 12/8/2012, parasitoid eclosion date: 1/1/2013, DHJPAR0051349, coll. date: 12/8/2012, parasitoid eclosion date: 12/31/2012, DHJPAR0051370. ♀, Elda Araya coll., host caterpillar: *Stenoma* Janzen144, coll. date: 7/21/2012, parasitoid eclosion date: 8/14/2012, DHJPAR0049649. [same as previous except food plant, coll. date and eclosion date] 2♀, food plant: *Guarea kegelii*, coll. date:12/23/2012, parasitoid eclosion date: 1/10/2012, DHJPAR0051360, coll. date: 12/23/2012, parasitoid eclosion date: 1/7/2013, DHJPAR0051371. ♀, Sendero Perdido, 10.8794N -85.38607W 620m., Carolina Cano coll., food plant: *Guarea kegelii*, coll. date: 10/9/2013, parasitoid eclosion date: 10/29/2013, DHJPAR0053649. [same as previous except coll. date and eclosion date] 2♂, 10/11/2013 10/26/2013, DHJPAR0053652, coll. date: 10/11/2013, parasitoid eclosion date: 10/29/2013, DHJPAR0053655. [same as previous except food plant, coll. date and eclosion date] ♀, food plant: *Trichilia adolfi*, coll. date: 10/9/2013, parasitoid eclosion date: 10/30/2013, DHJPAR0053654. ♂, Sendero Perdido, 10.8794N -85.38607W 620m., Gloria Sihezar coll., food plant: *Guarea kegelii*, 9/24/2013 10/9/2013, DHJPAR0053658. [same as previous except caterpillar, coll. date and eclosion date] ♂, host caterpillar: *Antaeotricha* Janzen07, 10/25/2013 11/22/2013, DHJPAR0054538. ♂, Sendero Perdido, 10.8794N -85.38607W 620m., Elda Araya coll., food plant: *Guarea kegelii*, coll. date: 1/9/2014, parasitoid eclosion date: 1/23/2014, DHJPAR0054515. [same as previous except coll. date and eclosion date] ♂, 1/9/2014 1/25/2014, DHJPAR0054517. ♀, Sendero Perdido, 10.8794N -85.38607W 620m., Elda Araya coll., food plant: *Guarea rhopalocarpa*, coll. date: 11/29/2013, parasitoid eclosion date: 12/29/2013, DHJPAR0054539. [same as previous except caterpillar and eclosion date] ♀, host caterpillar: *Antaeotricha thapsinopa*, parasitoid eclosion date: 12/31/2013, DHJPAR0054532. ♀, Sendero Perdido, 10.8794N -85.38607W 620m., Elda Araya coll., food plant: *Trichilia adolfi*, host caterpillar: *Antaeotricha* Janzen07, 2/1/2010 2/14/2010, DHJPAR0038910. ♀, Finca San Gabriel, 10.87766N -85.39343W 645m., Carolina Cano coll., food plant: *Guarea kegelii*, host caterpillar: *Antaeotricha* Janzen07, coll. date: 10/18/2013, parasitoid eclosion date: 11/2/2013, DHJPAR0054519. ♂, Finca San Gabriel, 10.87766N -85.39343W 645m., Carolina Cano coll., food plant: *Guarea rhopalocarpa*, host caterpillar: *Stenoma* Janzen144, coll. date: 8/11/2013, parasitoid eclosion date: 8/29/2013, DHJPAR0053645. ♀, Finca San Gabriel, 10.87766N -85.39343W 645m., Elda Araya coll., food plant: *Guarea kegelii*, coll. date: 1/6/2014, parasitoid eclosion date: 1/24/2014, DHJPAR0054511. ♂, Sendero Palo Alto, 10.88186N -85.38221W 570m., Carolina Cano coll., food plant: *Guarea rhopalocarpa*, coll. date: 9/12/2013, parasitoid eclosion date: 9/29/2013, DHJPAR0053618. [same as previous except eclosion date] ♂, parasitoid eclosion date: 10/2/2013, DHJPAR0053621. ♀, Cementerio Viejo, 10.88111N -85.38889W 570m., Carolina Cano coll., food plant: *Guarea kegelii*, coll. date: 9/10/2013, parasitoid eclosion date: 10/1/2013, DHJPAR0053611. [same as previous except eclosion date] ♂, parasitoid eclosion date: 9/30/2013, DHJPAR0053619. [same as previous except caterpillar, coll. date and eclosion date] ♀, host caterpillar: *Antaeotricha ribbei* 12/3/2013 12/21/2013, DHJPAR0054520. [same as previous except coll. date and eclosion date] ♀, 12/3/2013 12/21/2013, DHJPAR0054534. ♂, Sendero Corredor, 10.87868N -85.38963W 620m., Carolina Cano coll., food plant: *Trichilia adolfi*, host caterpillar: Depressariinae, *Antaeotricha* Janzen09, coll. date: 1/3/2014, parasitoid eclosion date: 1/25/2014, DHJPAR0054518. ♀, Rio Blanco Abajo, 10.90037N -85.37254W 500m., Elda Araya coll., coll. date: 8/11/2009, parasitoid eclosion date: 8/27/2009, DHJPAR0036722. ♂, Guanacaste, Sector Del Oro, Margarita, 11.03234N -85.43954W 380m., Lucia Ríos coll., host caterpillar: *Antaeotricha thapsinopa*, coll. date: 1/15/2005, parasitoid eclosion date: 2/1/2005, DHJPAR0015317.

**Figure 12.**
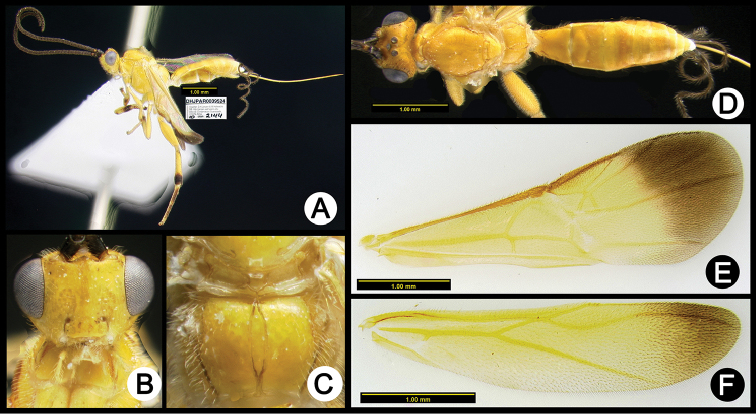
*Lytopylus gahyunae* holotype: **A** lateral habitus **B** anterior head **C** propodeum **D** dorsal habitus **E** fore wing **F** hind wing.

#### Lytopylus gisukae

Taxon classificationAnimaliaHymenopteraBraconidae

Kang sp. n.

http://zoobank.org/40757AA3-B52C-4733-AEF2-334C861F561F

Fig. 13

##### Diagnosis.

Vertex of head mostly pale; fore wing mostly infuscated with a quadrate second submarginal cell; mesoscutum entirely pale (yellow to orange); median areola of propodeum length 15x its width; anterior transverse carina of propodeum not reaching the lateral margin; median tergites entirely pale (yellow to orange).

##### Description.

Holotype: female. Body length 5.0 mm. Fore wing length 5.0 mm. Fore wing mostly infuscated with a quadrate second submarginal cell. Scutellar sulcus with one median longitudinal carina. Median areola of propodeum length 15x its width with well-defined margins. Anterior transverse carina of propodeum not reaching the lateral margin. Lateral longitudinal carinae of median tergite 1 well-defined. Median syntergite 2+3 1.2 times longer than wide. Ovipositor longer than metasoma, but shorter than body.

##### Male.

Unknown.

##### Etymology.

*Lytopylus gisukae* is named in honor of Gisuk Lee, mother-in-law of the first author.

##### Biology.

Reared one time from *Antaeotricha* Janzen405 (Stenomatinae, Depressariidae) feeding on mature leaves of *Astrocaryum alatum* (Arecaceae) in ACG rain forest at 420 m elevation.

##### Type material.

Holotype ♀: Costa Rica, Alajuela, Sector Rincon Rain Forest, Sendero Venado, Area de Conservaciόn Guanacaste 10.89678N -85.27001W 420m., Pablo Umaña coll., food plant: Arecaceae *Astrocaryum alatum*, host caterpillar: Depressariidae, Stenomatinae, *Antaeotricha* Janzen405, coll. date: 8/1/2011, parasitoid eclosion date: 9/11/2011, DHJPAR0048076.

**Figure 13.**
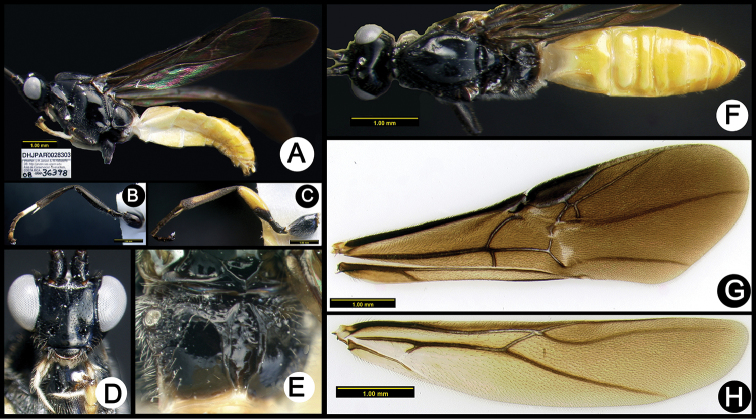
*Lytopylus gisukae* holotype: **A** lateral habitus **B** anterior head **C** propodeum **D** dorsal habitus **E** wings.

#### Lytopylus guillermopereirai

Taxon classificationAnimaliaHymenopteraBraconidae

Kang sp. n.

http://zoobank.org/FAC851A7-13CC-4DB7-AFDE-562014FE4D02

Fig. 14

##### Diagnosis.

Fore wing mostly infuscated; mesoscutum entirely melanic; scutellar sulcus lacking longitudinal carina; median tergites entirely melanic.

##### Description.

Holotype: female. Body length 5.4 mm. Fore wing length 4.5 mm. Fore wing mostly infuscated. Scutellar sulcus lacking longitudinal carina. Median areola of propodeum with well-defined margins. Anterior transverse carina of propodeum reaching the lateral margin. Lateral longitudinal carinae of median tergite 1 well-defined. Median syntergite 2+3 1.3 times longer than wide.

##### Female.

Unknown.

##### Etymology.

*Lytopylus guillermopereirai* is named in honor of Guillermo Pereira in recognition of his participation in the collaborative development of the ICE-ACG geothermal project of Pailas II, northwestern Costa Rica.

##### Biology.

Reared one time from elachJanzen01 Janzen726 (Depressariidae) feeding on *Sloanea faginea* (Elaeocarpaceae) in ACG rain forest at 645 m elevation.

##### Type material.

Holotype ♂: Costa Rica, Alajuela, Sector San Cristobal, Finca San Gabriel, Area de Conservaciόn Guanacaste 10.87766N -85.39343W 645m., Gloria Sihezar coll., food plant: Elaeocarpaceae *Sloanea faginea*, host caterpillar: Depressariidae, subfamily unknown, elachJanzen01 Janzen726, coll. date: 2/24/2014, parasitoid eclosion date: 3/17/2014, DHJPAR0055234.

**Figure 14.**
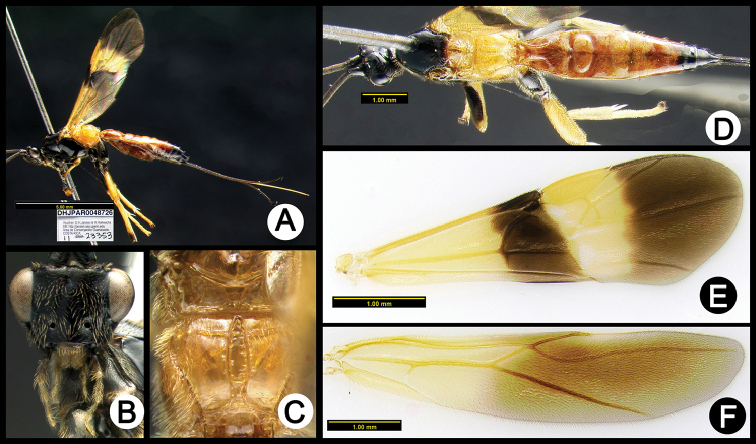
*Lytopylus guillermopereirai* holotype: **A** lateral habitus **B** anterior head **C** propodeum **D** dorsal habitus **E** fore wing **F** hind wing.

#### Lytopylus gustavoindunii

Taxon classificationAnimaliaHymenopteraBraconidae

Kang sp. n.

http://zoobank.org/9008E51A-5E03-4E95-BCF1-E427306B35F9

Fig. 15

##### Diagnosis.

Apical flagellomeres brown not distinctly paler than subapical flagellomeres; vertex of head entirely pale; fore wing mostly infuscated with a triangular second submarginal cell; mesoscutum entirely pale (yellow to orange); median areola spindle-shaped; anterior transverse carina of propodeum absent; median tergites entirely pale (yellow to orange).

##### Description.

Holotype: female. Body length 4.8 mm. Fore wing length 4.5 mm. Fore wing mostly infuscated. Scutellar sulcus with one median longitudinal carina. Median areola of propodeum with well-defined margins. Anterior transverse carina of propodeum absent. Lateral longitudinal carinae of median tergite 1 well-defined. Median syntergite 2+3 1.3 times longer than wide. Ovipositor slightly longer than body.

##### Male.

Unknown.

##### Etymology.

*Lytopylus gustavoindunii* is named in honor of Gustavo Induni in recognition of his participation in the collaborative development of the ICE-ACG geothermal project of Pailas II, northwestern Costa Rica.

##### Biology.

Reared 12 times from two species of palm-feeding (*Geonoma, Chamaedorea*) Depressariidae (*Stenoma* Janzen142 and *Stenoma* Janzen284) in the understory of ACG rain forest from 645-742 m elevation.

##### Type material.

Holotype ♀: Costa Rica, Alajuela, Sector San Cristobal, Sendero Perdido, Area de Conservaciόn Guanacaste 10.8794N -85.38607W 620m., Elda Araya coll., food plant: Arecaceae *Geonoma ferruginea*, host caterpillar: Depressariidae, Stenomatinae, *Stenoma* Janzen284, coll. date: 8/9/2013, parasitoid eclosion date: 8/28/2013, DHJPAR0053857. Paratypes: [the following have the same data as the holotype except as indicated] ♀, parasitoid eclosion date: 8/27/2013, DHJPAR0053648. ♀, parasitoid eclosion date: 8/31/2013, DHJPAR0053653. ♀, parasitoid eclosion date: 9/1/2013, DHJPAR0053646. ♀, Finca San Gabriel, 10.87766N -85.39343W 645m., host caterpillar: *Stenoma* Janzen142 coll. date: 11/16/2012, parasitoid eclosion date: 12/11/2012, DHJPAR0051369. [same as previous except eclosion date] ♀, parasitoid eclosion date: 12/20/2012, DHJPAR0051359. ♀, parasitoid eclosion date: 12/15/2012, DHJPAR0051372. ♀, Sendero Aguas Termales, geolocation unknown, food plant: *Chamaedorea tepejilote*, host caterpillar: *Stenoma* Janzen142, coll. date: 10/10/2010, parasitoid eclosion date: 10/29/2010, DHJPAR0041560.

**Figure 15.**
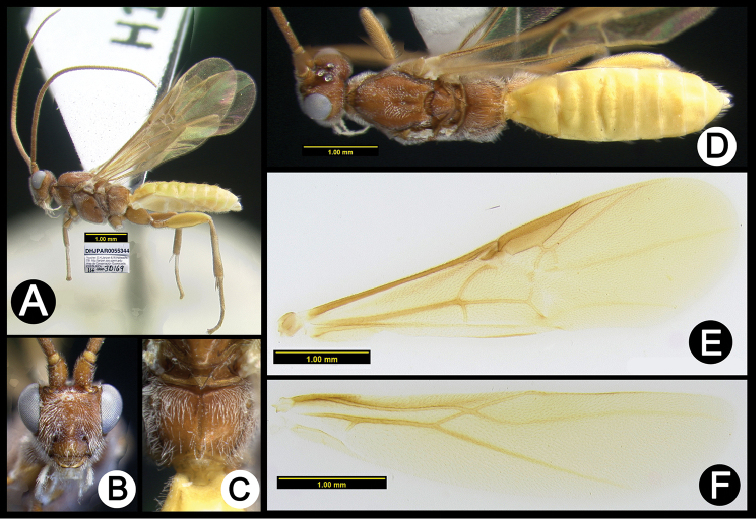
*Lytopylus gustavoindunii* holotype: **A** lateral habitus **B** anterior head **C** propodeum **D** dorsal habitus **E** fore wing **F** hind wing.

#### Lytopylus hartmanguidoi

Taxon classificationAnimaliaHymenopteraBraconidae

Kang sp. n.

http://zoobank.org/42792E91-4825-4562-93BE-7B2BB40A40FA

Fig. 16

##### Diagnosis.

Fore wing with one black band; mid tibia black basally and distally, yellow at mid-length.

##### Description.

Holotype: female. Body length 4.3 mm. Fore wing length 4.3 mm. Fore wing with one black band. Scutellar sulcus with one median longitudinal carina. Median areola of propodeum with well-defined margins. Anterior transverse carina of propodeum absent. Lateral longitudinal carinae of median tergite 1 well-defined. Median syntergite 2+3 1.3 times longer than wide. Ovipositor longer than metasoma, but shorter than body.

##### Male.

Unknown.

##### Etymology.

*Lytopylus hartmanguidoi* is named in honor of Hartman Guido in recognition of his participation in the collaborative development of the ICE-ACG geothermal project of Pailas II, northwestern Costa Rica.

##### Biology.

Reared five times from three species leaf-tiers in the Depressariidae, feeding on mature leaves of *Hiraea reclinata* (Malpighiaceae) at the intersection of the ACG dry forest and rain forest ecosystems at 540 m elevation.

##### Type material.

Holotype ♀: Costa Rica, Area de Conservaciόn Guanacaste, Alajuela, Sector San Cristobal, Tajo Angeles, 10.86472N -85.41531W 540m., Elda Araya coll., food plant: Malpighiaceae *Hiraea reclinata*, host caterpillar: Depressariidae, subfamily unknown, elachJanzen01 Janzen392, coll. date: 4/27/2010, parasitoid eclosion date: 5/19/2010, DHJPAR0039524. Paratypes: [the following have the same data as the holotype except as indicated] ♀, Gloria Sihezar coll., host caterpillar: *Psilocorsis* Janzen369, coll. date: 4/20/2010, parasitoid eclosion date: 5/15/2010, DHJPAR0039513. ♀, Gloria Sihezar coll., parasitoid eclosion date: 6/4/2010, DHJPAR0039514. ♀, Gloria Sihezar host caterpillar: Depressariidae, Stenomatinae, *Antaeotricha* Janzen126. 1/10/2011 2/6/2011, DHJPAR0042831. [same as previous except as coll. date and eclosion date] ♀, coll. date: 1/13/2011, parasitoid eclosion date: 2/8/2011, DHJPAR0042844.

**Figure 16.**
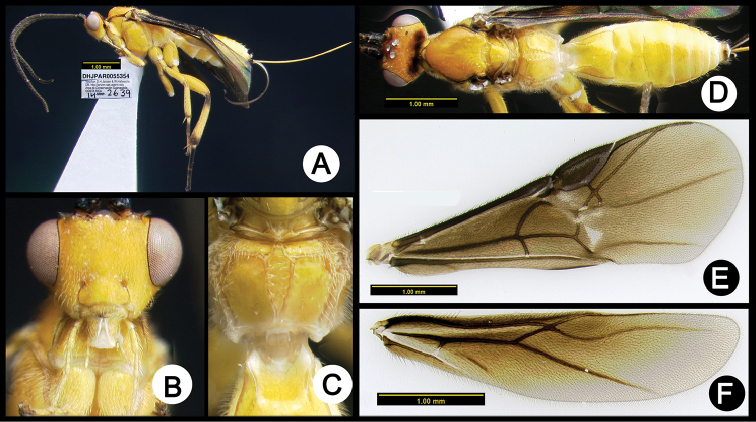
*Lytopylus hartmanguidoi* holotype: **A** lateral habitus **B** anterior head **C** propodeum **D** dorsal habitus **E** fore wing **F** hind wing.

#### Lytopylus hernanbravoi

Taxon classificationAnimaliaHymenopteraBraconidae

Kang sp. n.

http://zoobank.org/028AF18F-E671-4FC7-9447-6EAD6AA30616

Fig. 17

##### Diagnosis.

Fore wing mostly infuscated; hind coxa entirely black; mesoscutum entirely melanic; anterior transverse carina of propodeum reaching the lateral margin; median tergites entirely yellow.

##### Description.

Holotype: male. Body length 6.0 mm. Fore wing length 6.7 mm. Fore wing mostly infuscated. Scutellar sulcus lacking longitudinal carina. Median areola of propodeum with well-defined margins. Anterior transverse carina of propodeum reaching the lateral margin. Lateral longitudinal carinae of median tergite 1 well-defined. Median syntergite 2+3 1.1 times longer than wide.

##### Female.

Unknown.

##### Etymology.

*Lytopylus hernanbravoi* is named in honor of Hernan Bravo in recognition of his participation in the collaborative development of the ICE-ACG geothermal project of Pailas II, northwestern Costa Rica.

##### Biology.

Reared one time from *Anadasmus* Janzen08 (Depressariidae), a stenomine leaf-tier feeding on mature foliage of *Ocotea austinii* (Lauraceae) in ACG cloud forest at 1460 m elevation.

##### Type material.

Holotype ♂: Costa Rica, Guanacaste, Sector Cacao, Sendero Cima, Area de Conservaciόn Guanacaste 10.93328N -85.45729W 1460m., Harry Ramirez coll., food plant: Lauraceae *Ocotea austinii*, host caterpillar: Depressariidae, Stenomatinae, *Anadasmus* Janzen08, coll. date: 8/11/2008, parasitoid eclosion date: 9/6/2008, DHJPAR0028303.

**Figure 17.**
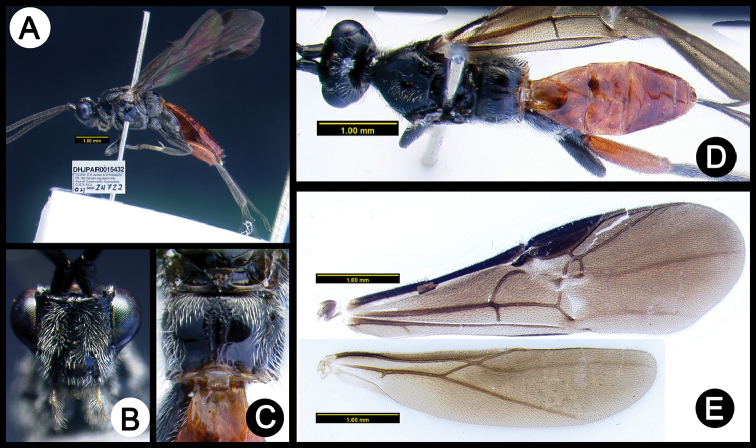
*Lytopylus hernanbravoi* holotype: **A** lateral habitus **B** mid leg **C** hind leg **D** anterior head **E** propodeum **F** dorsal habitus **G** fore wing **H** hind wing.

#### Lytopylus hokwoni

Taxon classificationAnimaliaHymenopteraBraconidae

Kang sp. n.

http://zoobank.org/21D4210B-AFC4-4FF0-9AE9-360D26C6C940

Fig. 18

##### Diagnosis.

Fore wing with two black bands; pronotum entirely melanic; lateral longitudinal carinae of median tergite 1 well-defined.

##### Description.

Holotype: female. Body length 8.0 mm. Fore wing length 4.6 mm. Fore wing with two black bands. Scutellar sulcus with one median longitudinal carina. Anterior transverse carina of propodeum reaching the lateral margin. Median areola of propodeum with well-defined margins. Lateral longitudinal carinae of median tergite 1 well-defined. Median syntergite 2+3 1.6 times longer than wide. Ovipositor slightly longer than body.

##### Male.

Mesoscutum bicolored. Mesopleuron pale.

##### Etymology.

Named in honor of Hokwon Kang, father of the first author.

##### Biology.

Reared nine times from seven species of stenomatine Depressariidae feeding as leaf-tiers on six species of plants in seven plant families at the intersection of the ACG dry forest and rain forest ecosystems at 280-640 m elevation.

##### Type material.

Holotype ♀: Costa Rica, Guanacaste, Sector El Hacha, Estacion Los Almendros, Area de Conservaciόn Guanacaste 11.03226N -85.52776W 290m., Elieth Cantillano coll., food plant: Clusiaceae *Clusia rosea*, host caterpillar: Depressariidae, Stenomatinae, *Stenoma* Janzen08, coll. date: 11/23/2011, parasitoid eclosion date: 12/10/2011, DHJPAR0048726. Paratypes: [the following have the same data as the holotype except as indicated] ♂, 290m., Lucia Ríos coll., coll. date: 9/19/2008, parasitoid eclosion date: 10/6/2008, DHJPAR0030605. ♀, Sendero Bejuquilla, 11.03004N -85.52699W 280m., food plant: Piperaceae *Peperomia angularis*, host caterpillar: Depressariidae, subfamily and species name unknown, coll. date: 1/11/2010, parasitoid eclosion date: 2/5/2010, DHJPAR0037940. ♀, Sendero Bejuquilla, 11.03004N -85.52699W 280m., Dunia Garcia coll., food plant: Hypericaceae *Vismia baccifera*, host caterpillar: *Cerconota recurvella*, coll. date: 10/26/2002, parasitoid eclosion date: 11/13/2002, DHJPAR0015414. ♀, Sector Cacao, Cuesta Caimito, 10.8908N -85.47192W 640m., Mariano Pereira coll., food plant: Hypericaceae *Vismia baccifera*, host caterpillar: *Cerconota recurvella*, coll. date: 5/16/2004, parasitoid eclosion date: 5/30/2004, DHJPAR0029302. ♀, Sector Cacao, Quebrada Otilio, 10.88996N -85.47966W 550m., Daniel Garcia coll., food plant: Fabaceae *Inga punctata*, host caterpillar: *Antaeotricha* Phillips01, coll. date: 9/24/2007, parasitoid eclosion date: 10/7/2007, DHJPAR0028279. ♀, Sector Pitilla, Cabrera, 11.00891N -85.40977W 500m., Calixto Moraga coll., food plant: Hypericaceae *Vismia baccifera*, host caterpillar: *Cerconota recurvella*, coll. date: 2/7/2007, parasitoid eclosion date: 2/14/2007, DHJPAR0017274. ♀, Alajuela, Sector Rincon Rain Forest, Sendero Tucan, 10.90424N -85.2712W 410m., Pablo Umaña Calderon coll., food plant: Melastomataceae *Miconia trinervia*, host caterpillar: elachJanzen01 Janzen211, coll. date: 4/7/2012, parasitoid eclosion date: 5/3/2012, DHJPAR0049051. ♀, Alajuela, Sector Rincon Rain Forest, Conguera, 10.91589N -85.26631W 420m., Jose Perez coll., food plant: Malpighiaceae *Banisteriopsis elegans*, host caterpillar: *Antaeotricha* Janzen127, coll. date: 5/17/2012, parasitoid eclosion date: 6/7/2012, DHJPAR0048714.

**Figure 18.**
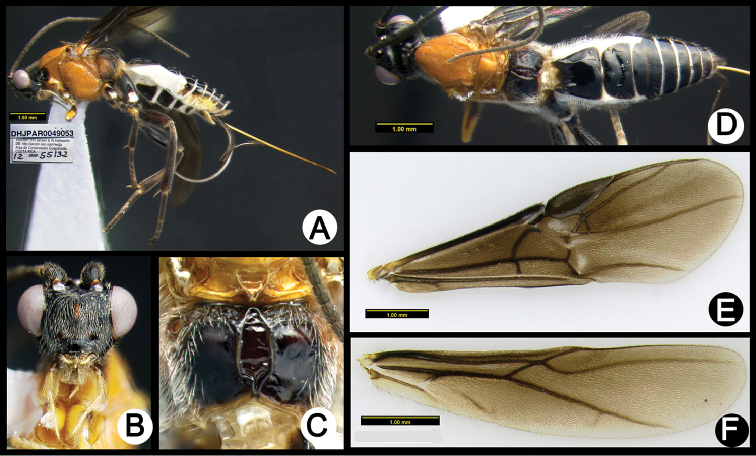
*Lytopylus hokwoni* holotype: **A** lateral habitus **B** anterior head **C** propodeum **D** dorsal habitus **E** fore wing **F** hind wing.

#### Lytopylus ivanniasandovalae

Taxon classificationAnimaliaHymenopteraBraconidae

Kang sp. n.

http://zoobank.org/11328F60-489F-41A1-B3FC-0F2EF21EAE40

Fig. 19

##### Diagnosis.

Fore wing with a slight yellow tinge; anterior transverse carina of propodeum absent; median tergites entirely pale.

##### Description.

Holotype: male. Body length 5.8 mm. Fore wing length 5.0 mm. Fore wing with a slight yellow tinge. Scutellar sulcus with four longitudinal carinae. Median areola of propodeum with well-defined margins. Anterior transverse carina of propodeum absent. Lateral longitudinal carinae of median tergite 1 well-defined. Median syntergite 2+3 as long as wide.

##### Female.

Unknown.

##### Etymology.

*Lytopylus ivanniasandovalae* is named in honor of Ivannia Sandoval in recognition of her participation in the collaborative development of the ICE-ACG geothermal project of Pailas II, northwestern Costa Rica.

##### Biology.

Reared one time from *Dichomerus* Janzen703 (Dichomeridinae, Gelechiidae) tying and feeding on mature leaves of *Neurolaena lobata* (Asteraceae) in ACG rain forest at 660 m elevation.

##### Type material.

Holotype ♂: Costa Rica, Guanacaste, Sector Pitilla, Sendero Carica, Area de Conservaciόn Guanacaste 10.99284N -85.42936W 660m., Calixto Moraga coll., food plant: Asteraceae *Neurolaena lobata*, host caterpillar: Gelechiidae, Dichomeridinae, *Dichomeris* Janzen703, coll. date: 1/25/2014, parasitoid eclosion date: 2/06/2014, DHJPAR0055344.

**Figure 19.**
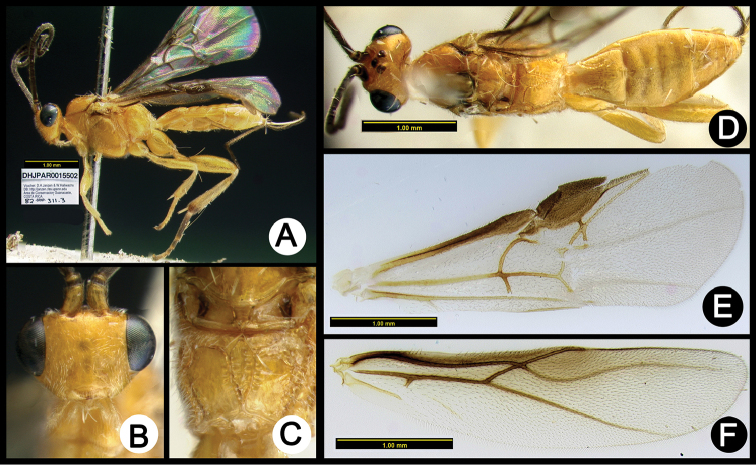
*Lytopylus ivanniasandovalae* holotype: **A** lateral habitus **B** anterior head **C** propodeum **D** dorsal habitus **E** fore wing **F** hind wing.

#### Lytopylus johanvalerioi

Taxon classificationAnimaliaHymenopteraBraconidae

Kang sp. n.

http://zoobank.org/862254CE-7A11-4D65-B7A6-69A723E1392C

Fig. 20

##### Diagnosis.

fore wing mostly infuscated; pronotum mostly yellow; mesoscutum mostly pale (yellow to orange); anterior transverse carina of propodeum reaching the lateral margin; median tergites mostly pale posterior tergum black.

##### Description.

Holotype: female. Body length 4.9 mm. Fore wing length 4.6 mm. Fore wing mostly infuscated. Scutellar sulcus with one median longitudinal carina. Median areola of propodeum with well-defined margins. Anterior transverse carina of propodeum reaching the lateral margin. Lateral longitudinal carinae of median tergite 1 well-defined. Median syntergite 2+3 0.9 times longer than wide. Ovipositor longer than metasoma, but shorter than body.

##### Males.

Occiput usually more melanic. Median tergites usually mostly pale with posterior terga black.

##### Etymology.

*Lytopylus johanvalerioi* is named in honor of Johan Valerio in recognition of his participation in the collaborative development of the ICE-ACG geothermal project of Pailas II, northwestern Costa Rica.

##### Biology.

Reared six times from two species of *Cerconota* leaf-tiers in the Depressariidae, feeding on mature leaves of three species of *Inga* (Fabaceae) in ACG rain forest at 540-645 m elevation.

##### Type material.

Holotype ♀: Costa Rica, Alajuela, Sector San Cristobal, Sendero Huerta, Area de Conservaciόn Guanacaste 10.9305N -85.37223W 527m., Gloria Sihezar coll., food plant: Fabaceae Inga oerstediana, host caterpillar: Depressariidae, Stenomatinae, Cerconota Janzen82, coll. date: 5/25/2014, parasitoid eclosion date: 6/7/2014, DHJPAR0055354. Paratypes: [the following have the same data as the holotype except as indicated] ♀, Brasilia, Moga, 11.01227N -85.34929W 320m., Duvalier Briceño coll., coll. date: 6/7/2012, parasitoid eclosion date: 6/19/2012, DHJPAR0049935. ♂, Guanacaste, Sector Pitilla, Estacion Quica, 10.99697N -85.39666W 470m., Ricardo Calero coll., food plant: Fabaceae Inga spectabilis, host caterpillar: Depressariidae, Stenomatinae, *Cerconota* Janzen216, coll. date: 5/25/2009, parasitoid eclosion date: 6/8/2009, DHJPAR0035498. [same as previous except coll. date and eclosion date] ♂, 5/27/2009 6/22/2009, DHJPAR0040066. ♂, Guanacaste, Sector Pitilla, Leonel, 10.99637N -85.40195W 510m., Mauricio Siezar coll., food plant: Inga spectabilis, host caterpillar: *Cerconota* Janzen216, coll. date: 6/15/2008, parasitoid eclosion date: 6/30/2008, DHJPAR0028298.

**Figure 20.**
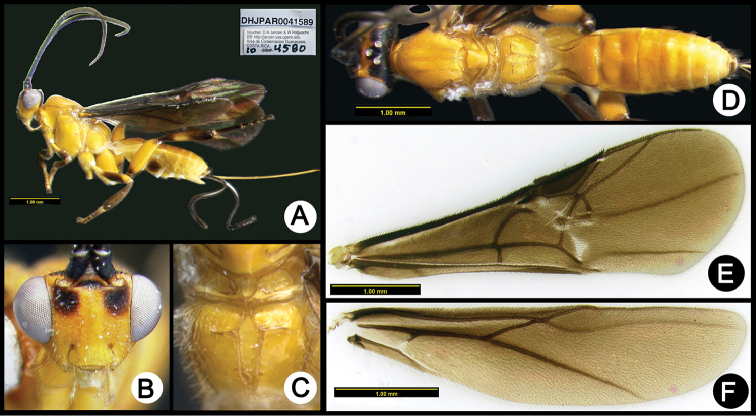
*Lytopylus johanvalerioi* holotype: **A** lateral habitus **B** anterior head **C** propodeum **D** dorsal habitus **E** fore wing **F** hind wing.

#### Lytopylus josecortesi

Taxon classificationAnimaliaHymenopteraBraconidae

Kang sp. n.

http://zoobank.org/F15FE11D-1DE7-4A48-B4C5-0DCA61C97445

Fig. 21

##### Diagnosis.

Fore wing mostly infuscated; mesoscutum entirely melanic; anterior transverse carina of propodeum absent; median areola of propodeum spindle-shape; lateral longitudinal carinae of median tergite 1 well-defined; median tergites entirely reddish orange.

##### Description.

Holotype: female. Body length 5.2 mm. Fore wing length 5.3 mm. Fore wing mostly infuscated. Scutellar sulcus with three longitudinal carinae. Median areola of propodeum with well-defined margins. Median areola length 6x its width. Median areola closed posteriorly. Anterior transverse carina of propodeum absent. Lateral longitudinal carinae of median tergite 1 well-defined. Median syntergite 2+3 1.1 times longer than wide. Ovipositor longer than metasoma, but shorter than body.

##### Male.

Similar to holotype.

##### Etymology.

*Lytopylus josecortesi* is named in honor of José Cortés in recognition of his participation in the collaborative development of the ICE-ACG geothermal project of Pailas II, northwestern Costa Rica.

##### Biology.

Reared two times from *Dichomeris* Janzen703 (Dichomeridinae, Gelechiidae) feeding on mature leaves of *Neurolaena lobata* (Asteraceae) in ACG dry forest – rain forest ecotone at 620 m elevation.

##### Type material.

Holotype ♀: Costa Rica, Guanacaste, Sector Del Oro, Bosque Aguirre, Area de Conservaciόn Guanacaste 11.0006N -85.438W 620m., Elieth Cantillano coll., food plant: Asteraceae *Neurolaena lobata*, host caterpillar: Gelechiidae, Dichomeridinae, *Dichomeris* Janzen703, coll. date: 9/21/2004, parasitoid eclosion date: 10/3/2004, DHJPAR0015432. Paratype: [the following have the same data as the holotype except as indicated] ♂, parasitoid eclosion date: 10/13/2004, DHJPAR0015431.

**Figure 21.**
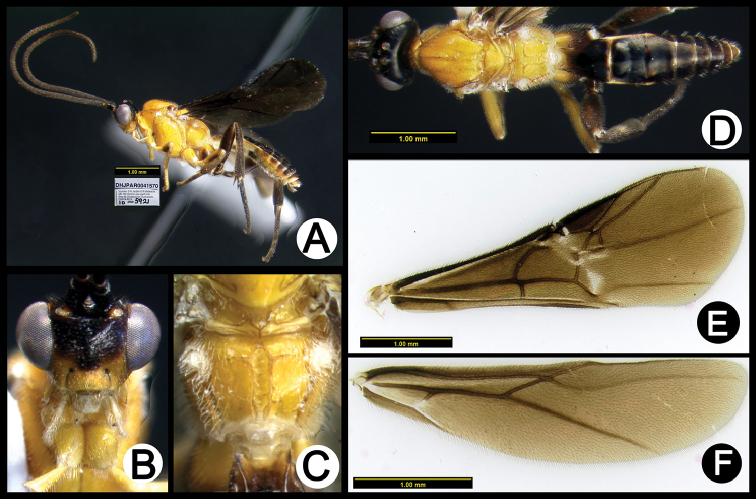
*Lytopylus josecortesi* holotype: **A** lateral habitus **B** anterior head **C** propodeum **D** dorsal habitus **E** fore wing **F** hind wing.

#### Lytopylus luisgaritai

Taxon classificationAnimaliaHymenopteraBraconidae

Kang sp. n.

http://zoobank.org/EE7D1A31-9D2F-4B74-A6A6-C95012768953

Fig. 22

##### Diagnosis.

Fore wing mostly infuscated; pronotum mostly pale, anteriorly melanic; mesoscutum entirely pale; mesopleuron entirely orange; scutellar sulcus lacking longitudinal carina; median tergites mostly melanic.

##### Description.

Holotype: female. Body length 6.7 mm. Fore wing length 6.3 mm. Fore wing mostly infuscated. Scutellar sulcus lacking longitudinal carina. Median areola of propodeum with well-defined margins. Anterior transverse carina of propodeum reaching the lateral margin. Lateral longitudinal carinae of median tergite 1 well-defined. Median syntergite 2+3 as long as wide. Ovipositor longer than metasoma, but shorter than body.

##### Male.

Unknown.

##### Variation.

Paratype propodeum mostly pale.

##### Etymology.

*Lytopylus luisgaritai* is named in honor of Luis Garita in recognition of his participation in the collaborative development of the ICE-ACG geothermal project of Pailas II, northwestern Costa Rica.

##### Biology.

Reared two times from *Oecophora* Janzen52 (Oecophorinae, Oecophoridae) feeding on mature leaves of *Clethra lanata* (Clethraceae) in ACG dry forest at 733–740 m elevation.

##### Type material.

Holotype ♀: Costa Rica, Guanacaste, Sector Mundo Nuevo, Camino Pozo Tres, Area de Conservaciόn Guanacaste 10.77079N -85.37422W 733m., Jose Cortez coll., food plant: Clethraceae *Clethra lanata*, host caterpillar: Depressariidae, Oecophorinae, *Oecophora* Janzen52, coll. date: 1/22/2012, parasitoid eclosion date: 3/3/2012, DHJPAR0049053. Paratype: [the following have the same data as the holotype except as indicated] ♀, Cerro Gongora Pelado, 10.76307N -85.41332W 740m., coll. date: 1/18/2014, parasitoid eclosion date: 2/22/2014, DHJPAR0055239.

**Figure 22.**
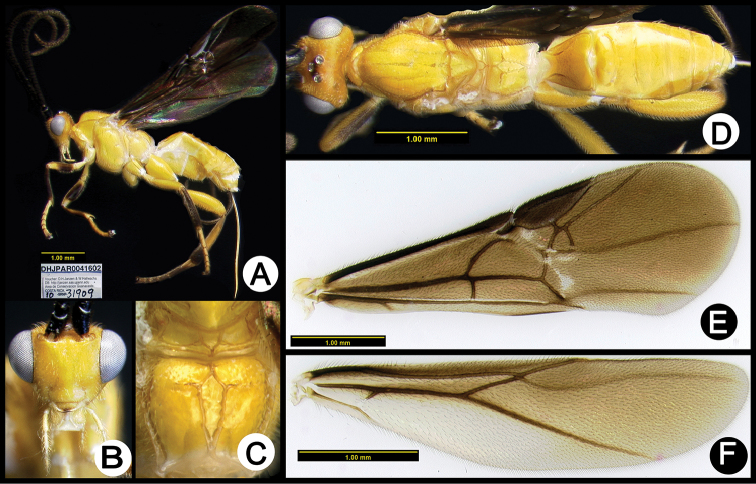
*Lytopylus luisgaritai* holotype: **A** lateral habitus **B** anterior head **C** propodeum **D** dorsal habitus **E** fore wing **F** hind wing.

#### Lytopylus mariamartachavarriae

Taxon classificationAnimaliaHymenopteraBraconidae

Kang sp. n.

http://zoobank.org/1691B66E-87CB-475D-AA54-0588AD1BCB13

Fig. 23

##### Diagnosis.

Fore wing hyaline; fore wing RS+Ma tubular on less than one third its length; anterior transverse carina of propodeum reaching the lateral margin; median tergites entirely pale.

##### Description.

Holotype: female. Body length 4.4 mm. Fore wing length 4.0 mm. Fore wing hyaline. Scutellar sulcus with three longitudinal carinae. Median areola of propodeum with well-defined margins. Anterior transverse carina of propodeum reaching the lateral margin. Lateral longitudinal carinae of median tergite 1 well-defined. Median syntergite 2+3 0.9 times longer than wide. Ovipositor longer than metasoma, but shorter than body.

##### Male.

Similar to holotype, but median tergites mostly pale with three posterior terga melanic.

##### Etymology.

*Lytopylus mariamartachavarriae* is named in honor of María Marta Chavarría in recognition of her participation in the collaborative development of the ICE-ACG geothermal project of Pailas II, northwestern Costa Rica.

##### Biology.

Reared four times from *Dichomeris santarosensis* (Dichomeridinae, Gelechiidae) feeding on new leaves of *Quercus oleoides* (Fagaceae) in ACG dry forest at 305 m elevation.

##### Type material.

Holotype ♀: Costa Rica, Guanacaste, Sector Santa Rosa, Arboles Via, Area de Conservaciόn Guanacaste 10.86081N -85.60828W 305m., Daniel H. Janzen coll., food plant: Fagaceae *Quercus oleoides*, host caterpillar: Gelechiidae, Dichomeridinae, *Dichomeris santarosensis*, coll. date: 6/24/1982, eclosion date unknown, DHJPAR0015502. Paratypes: [the following have the same data as the holotype except as indicated] 2♀, 1♂, DHJPAR0015501, DHJPAR0015503, DHJPAR0015500.

**Figure 23.**
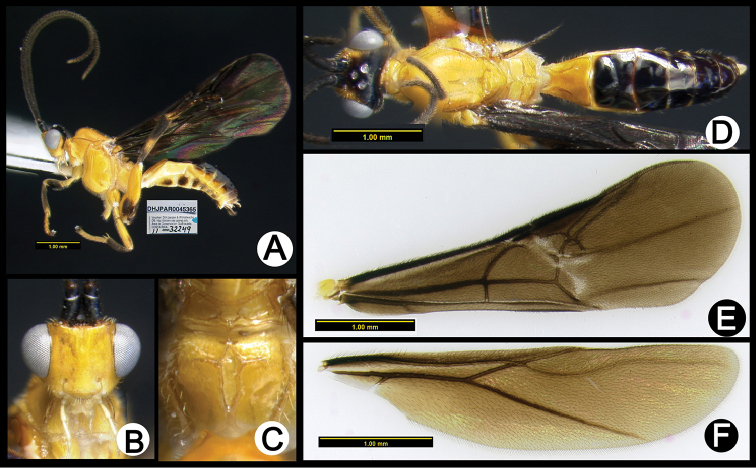
*Lytopylus mariamartachavarriae* holotype: **A** lateral habitus **B** anterior head **C** propodeum **D** dorsal habitus **E** fore wing **F** hind wing.

#### Lytopylus miguelviquezi

Taxon classificationAnimaliaHymenopteraBraconidae

Kang sp. n.

http://zoobank.org/1BE064F2-17DB-44CB-8B99-5A6F4224C7C1

Fig. 24 , 25

##### Diagnosis.

Apical flagellomeres brown not distinctly paler than subapical flagellomeres; fore wing mostly infuscated with a triangular second submarginal cell; fore tibia mostly melanic, yellow basally; hind tibia black basally and distally, yellow at mid-length; pronotum entirely pale (yellow to orange); mesoscutum entirely pale (yellow to orange); median areola of propodeum kite-shaped; anterior transverse carina of propodeum not reaching the lateral margin; median tergites mostly pale with posterior terga black; median syntergite 2+3 1.1 times longer than wide.

##### Description.

Holotype: female. Body length 5.1 mm. Fore wing length 4.9 mm. Fore wing mostly infuscated. Scutellar sulcus with one median longitudinal carina. Median areola of propodeum with well-defined margins. Anterior transverse carina of propodeum not reaching the lateral margin. Lateral longitudinal carinae of median tergite 1 well-defined. Median syntergite 2+3 1.1 times longer than wide. Ovipositor longer than metasoma, but shorter than body.

##### Males.

Body length usually shorter than holotype. Median tergites mostly melanic.

##### Variation.

Female anterior head varies from mostly pale to mostly melanic.

##### Etymology.

*Lytopylus miguelviquezi* is named in honor of Miguel Viquez in recognition of his participation in the collaborative development of the ICE-ACG geothermal project of Pailas II, northwestern Costa Rica.

##### Biology.

Reared 58 times from the *Dichomeris designatella* complex (21), gelJanzen01 Janzen179 (13), and gelJanzen01 Janzen485 (16), all leaf tying dichomeridine Gelechiidae feeding on mature leaves of two species of *Erythroxylum* (Erythroxylaceae) and two species of *Rinorea* (Violaceae) in ACG rain forest-dry forest ecotone, and rain forest at 109 to 540 m elevation.

**Figure 24.**
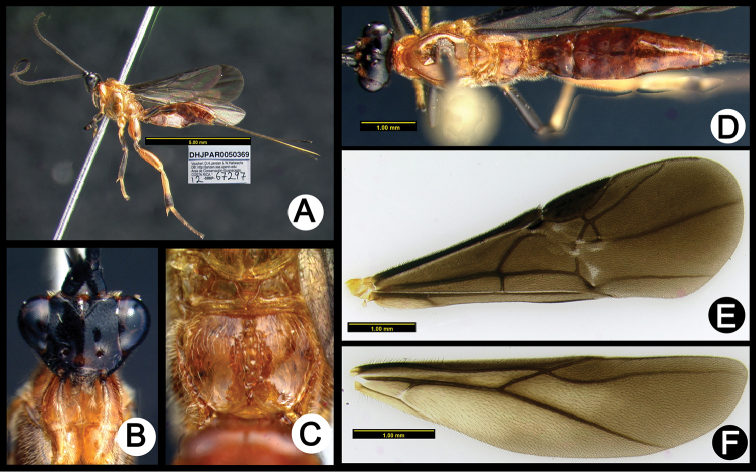
*Lytopylus miguelviquezi* holotype: **A** lateral habitus **B** anterior head **C** propodeum **D** dorsal habitus **E** fore wing **F** hind wing.

##### Type material.

Holotype ♀: Costa Rica, Alajuela, Sector San Cristobal, Tajo Angeles, Area de Conservaciόn Guanacaste 10.86472N -85.41531W 540m., Carolina Cano coll., food plant: Erythroxylaceae *Erythroxylum macrophyllum*, host caterpillar: Gelechiidae, Dichomeridinae, *Dichomeris* designatellaDHJ02, coll. date: 8/19/2010, parasitoid eclosion date: 9/8/2010, DHJPAR0041589. Paratypes: [the following have the same data as the holotype except as indicated] ♂, host caterpillar: *Dichomeris* designatellaDHJ03, coll. date: 1/25/2011, parasitoid eclosion date: 2/17/2011, DHJPAR0043147. ♀, coll. date: 7/8/2010, parasitoid eclosion date: 7/24/2010, DHJPAR0040341. ♀, coll. date: 7/8/2010, parasitoid eclosion date: 7/26/2010, DHJPAR0040347. ♀, food plant: Violaceae *Rinorea squamata*, host caterpillar: gelJanzen01 Janzen485, coll. date: 6/10/2010, parasitoid eclosion date: 6/26/2010, DHJPAR0040336. [same as previous except coll. date and eclosion date] ♀, coll. date: 10/15/2010, parasitoid eclosion date: 11/2/2010, DHJPAR0041566. ♂, coll. date: 10/15/2010, parasitoid eclosion date: 10/30/2010, DHJPAR0041570. ♂, coll. date: 10/15/2010, parasitoid eclosion date: 10/27/2010, DHJPAR0041565. ♂, coll. date: 10/20/2010, parasitoid eclosion date: 11/7/2010, DHJPAR0041567. ♂, coll. date: 11/7/2010, parasitoid eclosion date: 11/25/2010, DHJPAR0041555. ♂, coll. date: 11/7/2010, parasitoid eclosion date: 11/24/2010, DHJPAR0041553. ♀, Elda Araya coll., coll. date: 3/4/2010, parasitoid eclosion date: 4/6/2010, DHJPAR0038911. [same as previous except coll. date and eclosion date] ♀, coll. date: 3/4/2010, parasitoid eclosion date: 3/21/2010, DHJPAR0038920. ♀, coll. date: 3/4/2010, parasitoid eclosion date: 3/26/2010, DHJPAR0038918. ♀, coll. date: 3/4/2010, parasitoid eclosion date: 3/25/2010, DHJPAR0038906. ♀, coll. date: 3/4/2010, parasitoid eclosion date: 3/26/2010, DHJPAR0038912. ♀, coll. date: 3/4/2010, parasitoid eclosion date: 3/26/2010, DHJPAR0038916. ♀, coll. date: 6/25/2010, parasitoid eclosion date: 7/11/2010, DHJPAR0040340. ♀, coll. date: 10/25/2010, parasitoid eclosion date: 11/14/2010, DHJPAR0041575. ♀, food plant: Violaceae *Rinorea squamata*, host caterpillar: gelJanzen01 Janzen485, coll. date: 3/7/2010, parasitoid eclosion date: 3/24/2010, DHJPAR0038909. [same as previous except coll. date and eclosion date] ♀, coll. date: 6/10/2010, parasitoid eclosion date: 6/30/2010, DHJPAR0040502. ♀, coll. date: 10/19/2010, parasitoid eclosion date: 11/10/2010, DHJPAR0041563. ♀, host caterpillar: gelJanzen01 Janzen179, 5/6/2011 6/23/2011, DHJPAR0045296. [same as previous except and eclosion date] ♂, 5/6/2011 6/22/2011, DHJPAR0045305. ♀, parasitoid eclosion date: 6/20/2011, DHJPAR0045373. ♀, parasitoid eclosion date: 6/22/2011, DHJPAR0045371. ♀, parasitoid eclosion date: 6/20/2011, DHJPAR0045276. ♀, parasitoid eclosion date: 6/19/2011, DHJPAR0045372. ♀, parasitoid eclosion date: 6/25/2011, DHJPAR0045369. ♀, parasitoid eclosion date: 6/22/2011, DHJPAR0045368. [same as previous except food plant and eclosion date] ♂, food plant: *Rinorea deflexiflora*, coll. date: 6/4/2014, parasitoid eclosion date: 6/17/2014, DHJPAR0055506. ♀, Gloria Sihezar coll., coll. date: 6/25/2010, parasitoid eclosion date: 7/20/2010, DHJPAR0040327. [same as previous except coll. date and eclosion date] ♀, coll. date: 7/8/2010, parasitoid eclosion date: 7/30/2010, DHJPAR0040332. ♀, coll. date: 11/1/2010, parasitoid eclosion date: 11/25/2010, DHJPAR0041561. ♂, host caterpillar: *Dichomeris* designatellaDHJ03, coll date: 7/26/2010, parasitoid eclosion date: 8/8/2010, DHJPAR0040330. [same as previous except eclosion date] ♂, parasitoid eclosion date: 8/13/2010, DHJPAR0040474. ♂, parasitoid eclosion date: 8/12/2010, DHJPAR0040459. ♂, parasitoid eclosion date: 8/11/2010, DHJPAR0040348. ♀, parasitoid eclosion date: 8/13/2010, DHJPAR0040342. ♂, parasitoid eclosion date: 8/13/2010, DHJPAR0040483. ♂, parasitoid eclosion date: 8/17/2010, DHJPAR0041588. ♀, host caterpillar: *Dichomeris* designatellaDHJ02, coll. date: 9/28/2010, parasitoid eclosion date: 10/25/2010, DHJPAR0041592. [same as previous except coll. date and eclosion date] ♀, coll. date: 3/14/2010, parasitoid eclosion date: 3/29/2010, DHJPAR0038917. [same as previous except as indicated] ♂, food plant: Violaceae *Rinorea squamata*, host caterpillar: gelJanzen01 Janzen485, coll. date: 5/24/2010, parasitoid eclosion date: 6/7/2010, DHJPAR0039509. [same as previous except coll. date and eclosion date] ♂, coll. date: 5/24/2010, parasitoid eclosion date: 6/7/2010, DHJPAR0039516. ♂, coll. date: 5/24/2010, parasitoid eclosion date: 6/8/2010, DHJPAR0039508. ♀, coll. date: 10/30/2010, parasitoid eclosion date: 11/27/2010, DHJPAR0041574. ♀, Gloria Sihezar coll., food plant: Erythroxylaceae *Erythroxylum havanense*, coll. date: 6/25/2010, parasitoid eclosion date: 7/10/2010, DHJPAR0040326. [same as previous except as indicated] ♀, food plant: *Erythroxylum macrophyllum*, host caterpillar: *Dichomeris* designatellaDHJ02, coll. date: 2/9/2011, parasitoid eclosion date: 3/1/2011, DHJPAR0042843. ♀, Osvaldo Espinoza coll., food plant: Violaceae *Rinorea squamata*, host caterpillar: gelJanzen01 Janzen179, coll. date: 2/9/2011, parasitoid eclosion date: 2/26/2011, DHJPAR0042842. ♂, Osvaldo Espinoza coll., coll. date: 8/29/2010, parasitoid eclosion date: 9/12/2010, DHJPAR0041597. ♀, Rio Blanco Abajo, 10.90037N -85.37254W 500m., Gloria Sihezar coll., food plant: Violaceae *Rinorea squamata*, host caterpillar: gelJanzen01 Janzen179, coll. date: 6/9/2011, parasitoid eclosion date: 6/24/2011, DHJPAR0045374. ♂, Sector Rincon Rain Forest, Sendero Anonas, 10.90528N -85.27882W 405m., Anabelle Cordoba coll., food plant: Violaceae *Rinorea hummelii*, host caterpillar: gelJanzen01 Janzen485, coll. date: 5/20/2014, parasitoid eclosion date: 6/10/2014, DHJPAR0055484. ♀, Sector Rincon Rain Forest, Quebrada Bambu, 10.9301N -85.25205W 109m., Cirilo Umaña coll., food plant: Violaceae *Rinorea deflexiflora*, host caterpillar: gelJanzen01 Janzen179, coll. date: 5/29/2014, parasitoid eclosion date: 6/10/2014, DHJPAR0055562. ♀, Guanacaste, Sector Del Oro, Quebrada Raiz, 11.02865N -85.48669W 280m., Roster Moraga coll., food plant: Violaceae *Rinorea deflexiflora*, host caterpillar: gelJanzen01 Janzen485, coll. date 6/3/2005, parasitoid eclosion date: 5/21/2005, DHJPAR0015528. ♀, Guanacaste, Sector Del Oro, Canyon Rio Mena, 10.99616N -85.45562W 560m., Lucia Ríos coll., coll. date: 3/26/2009, parasitoid eclosion date: 5/3/2009, DHJPAR0037860. ♂, Guanacaste, Sector Del Oro, Meteorologico, 11.00199N -85.46166W 590m., Lucia Ríos coll., coll. date: 9/3/2010, parasitoid closion date: 9/21/2010, DHJPAR0041949.

**Figure 25.**
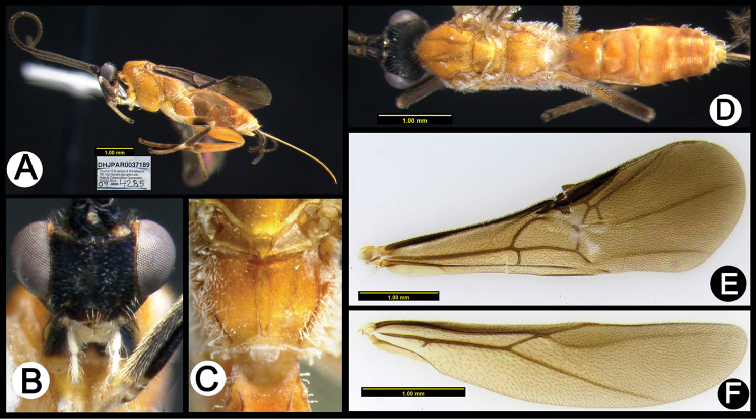
*Lytopylus miguelviquezi* male: **A** lateral habitus **B** anterior head **C** propodeum **D** dorsal habitus **E** fore wing **F** hind wing.

#### Lytopylus motohasegawai

Taxon classificationAnimaliaHymenopteraBraconidae

Kang sp. n.

http://zoobank.org/2F55247B-D2C7-4931-982F-7105C023953C

Fig. 26 , 27

##### Diagnosis.

Vertex of head entirely yellow; fore wing mostly infuscated with a quadrate second submarginal cell; mesoscutum mostly or entirely pale (yellow to orange); median areola of propodeum kite-shaped; anterior transverse carina of propodeum not reaching the lateral margin; median tergites entirely pale (yellow to orange).

##### Description.

Holotype: female. Body length 4.9 mm. Fore wing length 4.9 mm. Fore wing mostly infuscated. Scutellar sulcus with one median longitudinal carina. Median areola of propodeum with well-defined margins. Anterior transverse carina of propodeum not reaching the lateral margin. Lateral longitudinal carinae of median tergite 1 well-defined. Median syntergite 2+3 as long as wide. Ovipositor longer than metasoma, but shorter than body.

##### Males.

Vertical of head and occiput usually mostly melanic. Body length usually shorter than holotype. Median tergites mostly melanic.

##### Variation.

Female occiput varies from entirely pale to mostly pale. Male hind femur color varies from mostly pale to black and pale with a similar percentage of each color.

##### Etymology.

*Lytopylus motohasegawai* is named in honor of Motohiro Hasegawa in recognition of his participation in the collaborative development of the ICE-ACG geothermal project of Pailas II, northwestern Costa Rica.

##### Biology.

Reared 36 times from gelJanzen01 Janzen28, a leaf-tier in the Gelechiidae feeding on mature leaves of two species of *Roupala* (Proteaceae) in ACG rain forest at 415 to 740 m elevation.

**Figure 26.**
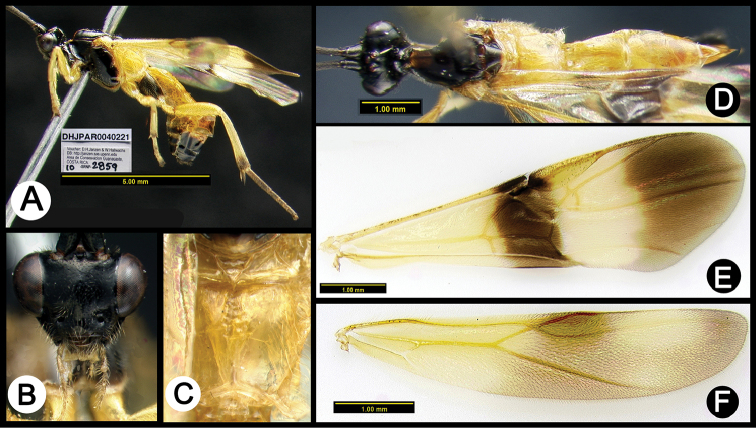
*Lytopylus motohasegawai*, holotype: **A** lateral habitus **B** anterior head **C** propodeum **D** dorsal habitus **E** fore wing **F** hind wing.

##### Type material.

Holotype ♀: Costa Rica, Guanacaste, Sector Pitilla, Sendero Naciente, Area de Conservaciόn Guanacaste 10.98705N -85.42816W 700m., Manuel Rios coll., food plant: Proteaceae *Roupala glaberrima*, host caterpillar: Gelechiidae, subfamily unknown, gelJanzen01 Janzen28, coll. date: 8/30/2010, parasitoid eclosion date: 9/23/2010, DHJPAR0041602. Paratypes: [the following have the same data as the holotype except as indicated] ♀, coll. date: 10/13/2010, parasitoid eclosion date: 11/1/2010, DHJPAR0041962. ♀, coll. date: 8/6/2011, parasitoid eclosion date: 9/10/2011, DHJPAR0048071. ♀, parasitoid eclosion date: 8/29/2011, DHJPAR0045318. ♂, parasitoid eclosion date: 9/8/2011, DHJPAR0048064. ♀, parasitoid eclosion date: 9/8/2011, DHJPAR0048068. ♀, Petrona Rios coll., parasitoid eclosion date: 8/31/2011, DHJPAR0048067. [same as previous except eclosion date] ♂, parasitoid eclosion date: 8/29/2011, DHJPAR0045364. ♂, Calixto Moraga coll., coll. date: 8/9/2011, parasitoid eclosion date: 8/29/2011, DHJPAR0045365. [same as previous except coll. date and eclosion date] ♀, coll. date: 1/17/2011, parasitoid eclosion date: 2/21/2011, DHJPAR0042463. ♀, Sendero Memos, 10.98171N -85.42785W 740m., Petrona Rios coll., coll. date: 8/8/2011, parasitoid eclosion date: 9/18/2011, DHJPAR0048065. [same as previous except eclosion date] ♀, parasitoid eclosion date: 9/9/2011, DHJPAR0048063. ♀, parsitoids eclosion date: 9/10/2011, DHJPAR0048066. ♀, Sendero Nacho, 10.98445N -85.42481W 710m., Petrona Rios coll., coll. date: 8/24/2010, parasitoid eclosion date: 10/1/2010, DHJPAR0041601. [same as previous except as indicated] ♀, Manuel Rios coll., coll. date: 9/27/2010, parasitoid eclosion date: 10/21/2010, DHJPAR0041203. [same as previous except eclosion date] ♂, parasitoid eclosion date: 11/2/2010, DHJPAR0041960. ♀, parasitoid eclosion date: 11/3/2010, DHJPAR0041963. ♂, parasitoid eclosion date: 10/28/2010, DHJPAR0041954. ♂, Manguera, 10.9959N -85.39842W 470m., Manuel Rios coll., coll. date: 1/4/2011, parasitoid eclosion date: 1/29/2011, DHJPAR0041577. [same as previous except as indicated] ♀, Ricardo Calero coll., coll. date: 7/15/2011, parasitoid eclosion date: 8/6/2011, DHJPAR0045330. [same as previous except eclosion date] ♀, parasitoid eclosion date: 9/12/2011, DHJPAR0048062. ♀, parasitoid eclosion date: 9/13/2011, DHJPAR0048061. ♀, parasitoid eclosion date: 9/13/2011, DHJPAR0048059. [same as previous except coll. date and eclosion date] ♀, coll. date: 9/11/2011 parasitoid eclosion date: 9/29/2011, DHJPAR0048060. ♂, coll. date: 9/18/2011 parasitoid eclosion date: 10/20/2011, DHJPAR0048073. ♀, coll. date: 9/18/2011 parasitoid eclosion date: 10/24/2011, DHJPAR0048072. ♀, Sendero Cuestona, 10.99455N -85.41461W 640m., Freddy Quesada coll., coll. date: 8/25/2011, parasitoid eclosion date: 9/14/2011, DHJPAR0048070. ♀, Alajuela, Sector San Cristobal, Tajo Angeles, 10.86472N -85.41531W 540m., Elda Araya coll., food plant: Proteaceae *Roupala montana*, coll. date: 10/9/2010, parasitoid eclosion date: 10/30/2010, DHJPAR0041573. [same as previous except coll. date and eclosion date] ♀, coll. date: 10/23/2010, parasitoid eclosion date: 11/12/2010, DHJPAR0041568. ♀, coll. date: 10/23/2010, parasitoid eclosion date: 12/3/2010, DHJPAR0041564. ♀, coll. date: 12/22/2010, parasitoid eclosion date: 1/28/2011, DHJPAR0041584. ♂, Sector Rincon Rain Forest, Jacobo, 10.94076N -85.3177W 461m, Edwin Apu coll., food plant: Proteaceae *Roupala glaberrima*, coll. date: 1/18/2014, parasitoid eclosion date: 2/6/2014, DHJPAR0054745. ♀, Estacion Caribe, 10.90187N -85.27495W 415m., Pablo Umaña Calderon coll., food plant: Proteaceae *Roupala montana*, coll. date: 7/31/2009, parasitoid eclosion date: 8/19/2009, DHJPAR0040071.

**Figure 27.**
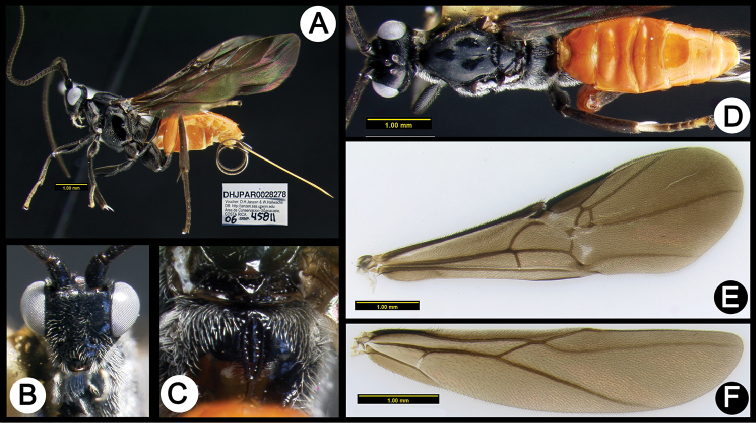
*Lytopylus motohasegawai* male: **A** lateral habitus **B** anterior head **C** propodeum **D** dorsal habitus **E** fore wing **F** hind wing.

#### Lytopylus okchunae

Taxon classificationAnimaliaHymenopteraBraconidae

Kang sp. n.

http://zoobank.org/36FA08EB-98CC-45A1-828D-72542BE201E9

Fig. 28

##### Diagnosis.

Apical flagellomeres brown not distinctly paler than subapical flagellomeres; vertex of head entirely melanic; fore wing mostly infuscated; mesoscutum entirely orange; anterior transverse carina of propodeum absent; median tergites entirely orange; median syntergite 2+3 1.5 times longer than wide.

##### Description.

Holotype: female. Body length 6.8 mm. Fore wing length 6.3 mm. Fore wing mostly infuscated. Scutellar sulcus with one median longitudinal carina. Median areola of propodeum with well-defined margins. Anterior transverse carina of propodeum absent. Lateral longitudinal carinae of median tergite 1 well-defined. Median syntergite 2+3 1.5 times longer than wide. Ovipositor about same length as body.

##### Males.

Similar to holotype except for median tergite color. Median tergites usually mostly pale with posterior three terga melanic.

##### Etymology.

*Lytopylus okchunae* is named in honor of Okchun Kim, grandmother of the first author.

##### Biology.

Reared 18 times from three species of *Antaeotricha* (Depressariidae) leaf-webbers feeding on mature leaves of five species of broad-leafed monocots (Hylaeanthe, Renealmia, Hedychium, Pleiostachya, Calathea) in the Marantaceae and Zingiberaceae in ACG rain forest 96-575 m elevation.

##### Type material.

Holotype ♀: Costa Rica, Alajuela, Sector Rincon Rain Forest, Palomo, Area de Conservaciόn Guanacaste 10.96187N -85.28045W 96m., Cirilo Umaña coll., food plant: Marantaceae *Pleiostachya leiostachya*, host caterpillar: Depressariidae, Stenomatinae, *Antaeotricha* Janzen224, coll. date: 2/20/2012 parasitoid eclosion date: 3/4/2012, DHJPAR0050369. Paratypes: [the following have the same data as the holotype except as indicated] ♀, Sendero Anonas, 10.90528N -85.27882W 405m., Jose Perez coll., food plant: Marantaceae *Hylaeanthe hoffmannii*, host caterpillar: *Antaeotricha* Janzen78, coll. date: 12/9/2010 parasitoid eclosion date: 1/2/2011, DHJPAR0041164. ♂, Anabelle Cordoba coll., food plant: Zingiberaceae *Renealmia cernua*, host caterpillar: *Antaeotricha* Janzen727, coll. date: 12/4/2012, parasitoid eclosion date: 12/23/2012, DHJPAR0051363. ♀, Quebrada Bambu, 10.9301N -85.25205W 109m., Cirilo Umaña coll. food plant: *Hylaeanthe hoffmannii*, host caterpillar: *Antaeotricha* Janzen78, coll. date: 10/29/2012, parasitoid eclosion date: 11/16/2012, DHJPAR0050939. [same as previous except coll. date and eclosion date] ♀, coll. date: 2/14/2013, parasitoid eclosion date: 3/24/2013, DHJPAR0051910. ♂, coll. date: 12/30/2014, parasitoid eclosion date: 1/16/2015, DHJPAR0057424. ♂, coll. date: 12/30/2014, parasitoid eclosion date: 1/14/2015, DHJPAR0056977. ♀, coll. date: 1/6/2015, parasitoid eclosion date: 1/20/2015, DHJPAR0056982. ♀, coll. date: 1/6/2015, parasitoid eclosion date: 1/20/2015, DHJPAR0056980. ♂, Finca Esmeralda, 10.93548N -85.25314W 123m., Cirilo Umaña coll., food plant: *Hylaeanthe hoffmannii*, host caterpillar: *Antaeotricha* Janzen78, coll. date: 1/6/2015, parasitoid eclosion date: 1/18/2015, DHJPAR0056981. ♀, Sector San Cristobal, Sendero Colegio, 10.89296N -85.3788W 520m., Carolina Cano coll. food plant: *Hylaeanthe hoffmannii*, host caterpillar: *Antaeotricha* Janzen78, coll. date: 9/30/2009, parasitoid eclosion date: 10/12/2009, DHJPAR0037191. ♀, Estacion San Gerardo, 10.88009N -85.38887W 575m., Gloria Sihezar coll. food plant: Zingiberaceae *Hedychium coronarium* (introduced), host caterpillar: *Antaeotricha* Janzen727, coll. date: 5/1/2014, parasitoid eclosion date: 5/16/2014, DHJPAR0055345. [same as previous except coll. date and eclosion date] ♀, coll. date: 5/1/2014, parasitoid eclosion date: 5/17/2014, DHJPAR0055355. ♀, coll. date: 5/1/2014, parasitoid eclosion date: 5/19/2014, DHJPAR0055984. ♀, Rio Blanco Abajo, 10.90037N -85.37254W 500m., Gloria Sihezar coll., food plant: Zingiberaceae *Hedychium coronarium* (introduced), host caterpillar: *Antaeotricha* Janzen727, coll. date: 5/9/2014, parasitoid eclosion date: 6/1/2014, DHJPAR0055819. ♀, Guanacaste, Sector Pitilla, Pasmompa, 11.01926N -85.40997W 440m., Manuel Rios coll., food plant: *Calathea marantifolia*, host caterpillar: *Antaeotricha* Janzen78, coll. date: 12/1/2005, parasitoid eclosion date: 12/16/2005, DHJPAR0015529.

**Figure 28.**
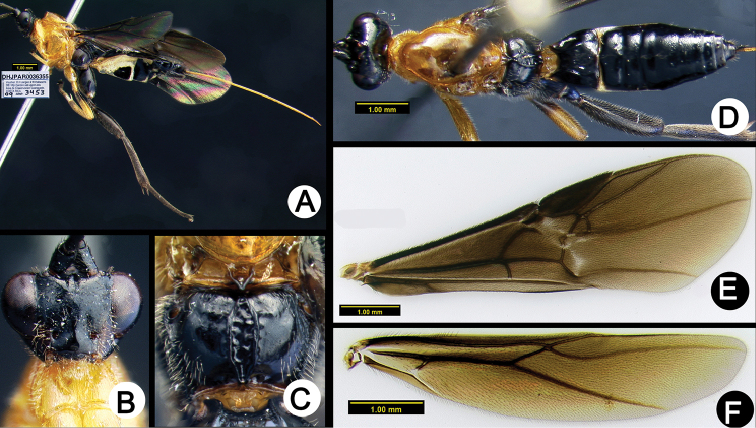
*Lytopylus okchunae* holotype: **A** lateral habitus **B** anterior head **C** propodeum **D** dorsal habitus **E** fore wing **F** hind wing.

#### Lytopylus pablocobbi

Taxon classificationAnimaliaHymenopteraBraconidae

Kang sp. n.

http://zoobank.org/388474DC-9ED2-4C53-A0EA-46C8AA3EAFB4

Fig. 29

##### Diagnosis.

Vertex of head entirely melanic; fore wing mostly infuscated; pronotum mostly orange, anteriorly black; mesoscutum entirely orange; anterior transverse carina of propodeum absent; median tergites entirely pale (yellow to orange); median syntergite 2+3 1.1 times longer than wide.

##### Description.

Holotype: female. Body length 4.8 mm. Fore wing length 5.0 mm. Fore wing mostly infuscated. Pronotum bicolored. Scutellar sulcus with one median longitudinal carina. Median areola of propodeum with well-defined margins. Anterior transverse carina of propodeum absent. Lateral longitudinal carinae of median tergite 1 well-defined. Median syntergite 2+3 1.1 times longer than wide. Ovipositor longer than metasoma, but shorter than body.

##### Male.

Unknown.

##### Etymology.

*Lytopylus pablocobbi* is named in honor of Pablo Cobb in recognition of his participation in the collaborative development of the ICE-ACG geothermal project of Pailas II, northwestern Costa Rica.

##### Biology.

Reared one time from elachJanzen01 Janzen640 (Depressariidae), a stenomine leaf-tier feeding on mature foliage of *Bunchosia odorata* (Malpighiaceae) in ACG dry forest – rain forest ecotone at 722 m elevation.

##### Type material.

Holotype ♀: Costa Rica, Alajuela, Sector San Cristobal, Jardin Estrada, Area de Conservaciόn Guanacaste 10.86546N -85.39694W 722m., Gloria Sihezar coll., food plant: Malpighiaceae *Bunchosia odorata*, host caterpillar: Depressariidae, Stenomatinae, elachJanzen01 Janzen640, coll. date: 8/19/2009, parasitoid eclosion date: 9/16/2009, DHJPAR0037189.

**Figure 29.**
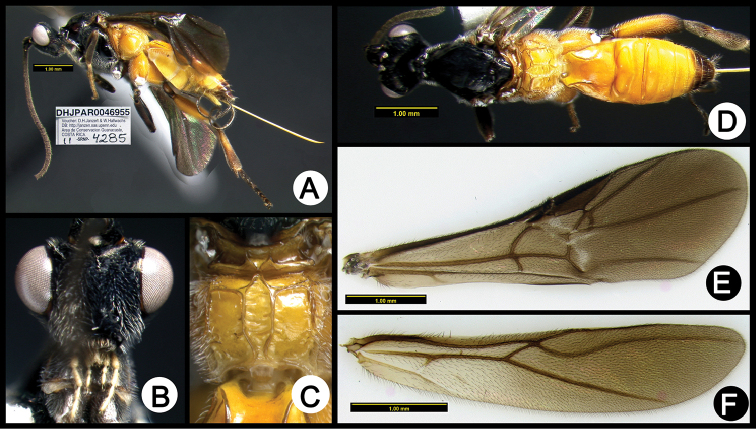
*Lytopylus pablocobbi* holotype: **A** lateral habitus **B** anterior head **C** propodeum **D** dorsal habitus **E** fore wing **F** hind wing.

#### Lytopylus robertofernandezi

Taxon classificationAnimaliaHymenopteraBraconidae

Kang sp. n.

http://zoobank.org/E4012DEC-AAA8-4964-A1D2-F714C99EC057

Fig. 30

##### Diagnosis.

Fore wing with two black bands; pronotum entirely black; lateral longitudinal carinae of median tergite 1 blunt.

##### Description.

Holotype: male. Body length 7.6 mm. Fore wing length 6.7 mm. Fore wing with two black bands. Scutellar sulcus with two longitudinal carinae. Anterior transverse carina of propodeum absent. Median areola of propodeum lacking well-defined margins. Lateral longitudinal carinae of median tergite 1 blunt. Median syntergite 2+3 1.6 times longer than wide.

##### Female.

Unknown.

##### Etymology.

*Lytopylus robertofernandezi* is named in honor of Roberto Fernández in recognition of his participation in the collaborative development of the ICE-ACG geothermal project of Pailas II, northwestern Costa Rica.

##### Biology.

Reared only one time and from the leaf-tier *Stenoma* Janzen687 (Depressariidae) feeding on mature leaves of *Pouteria exfoliata* (Sapotaceae) at the intersection of the ACG dry forest and rain forest ecosystems at 540 m elevation.

##### Type material.

Holotype ♂: Costa Rica, Alajuela, Sector San Cristobal, Tajo Angeles, Area de Conservaciόn Guanacaste 10.86472N -85.41531W 540m., Carolina Cano coll., food plant: Sapotaceae *Pouteria exfoliata*, host caterpillar: Depressariidae, Stenomatinae, *Stenoma* Janzen687, coll. date: 6/7/2010, parasitoid eclosion date: 6/23/2010, DHJPAR0040221.

**Figure 30.**
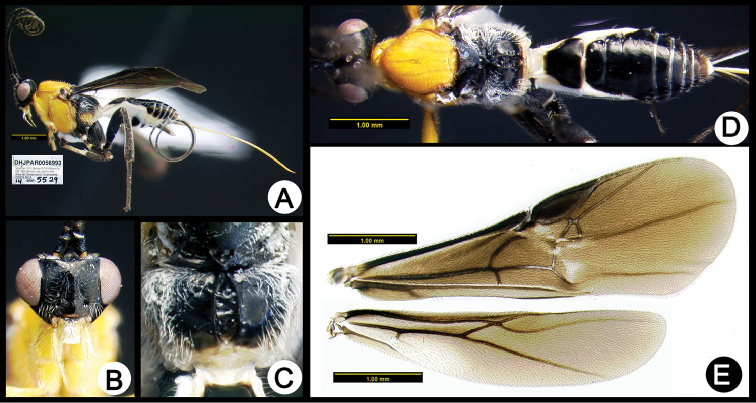
*Lytopylus robertofernandezi* holotype: **A** lateral habitus **B** anterior head **C** propodeum **D** dorsal habitus **E** fore wing **F** hind wing.

#### Lytopylus rogerblancoi

Taxon classificationAnimaliaHymenopteraBraconidae

Kang sp. n.

http://zoobank.org/DE0D5410-134C-43BF-8A55-51B006AE8C76

Fig. 31

##### Diagnosis.

Fore wing mostly infuscated; mesoscutum entirely black; median areola of propodeum with well-defined margins; anterior transverse carina of propodeum absent; median tergites entirely orange; lateral longitudinal carinae of median tergite 1 blunt.

##### Description.

Holotype: female. Body length 6.0 mm. Fore wing length 6.4 mm. Fore wing mostly infuscated. Scutellar sulcus with three longitudinal carinae. Median areola of propodeum with well-defined margins. Anterior transverse carina of propodeum absent. Lateral longitudinal carinae of median tergite 1 blunt. Ovipositor longer than metasoma, but shorter than body. Median syntergite 2+3 0.9 times longer than wide.

##### Male.

Similar to holotype.

##### Etymology.

*Lytopylus rogerblancoi* is named in honor of Roger Blanco in recognition of his participation in the collaborative development of the ICE-ACG geothermal project of Pailas II, northwestern Costa Rica.

##### Biology.

Reared eight times, one time from a waif pupa and seven times from gelJanzen01 Janzen356 (Dichomeridinae, Gelechiidae) feeding on mature leaves of *Hampea* and *Mortoniodendron* (Malvaceae) in ACG rainforest at 600 to 1180 m elevation.

##### Type material.

Holotype ♀: Costa Rica, Alajuela, Sector San Cristobal, Finca San Gabriel, Area de Conservaciόn Guanacaste 10.87766N -85.39343W 645m., Carolina Cano coll., food plant: Malvaceae *Mortoniodendron costaricense*, host caterpillar: Gelechiidae, Dichomeridinae, gelJanzen01 Janzen356, coll. date: 1/28/2010, parasitoid eclosion date: 2/25/2010, DHJPAR0038905. Paratypes: [the following have the same data as the holotype except as indicated] ♀, coll. date: 11/30/2012, parasitoid eclosion date: 1/1/2013, DHJPAR0051361. ♂, coll. date: 2/21/2013, parasitoid eclosion date: 3/18/2013, DHJPAR0051914. ♂, Jardin Estrada, 10.86546N -85.39694W 722m., Gloria Sihezar coll., coll. date: 12/10/2013, parasitoid eclosion date: 1/14/2014, DHJPAR0054536. ♂, Guanacaste, Sendero Segundo, 10.92679N -85.45332W 1180m., Manuel Pereira coll., food plant: Malvaceae *Hampea appendiculate*, coll. date: 7/30/2007, parasitoid eclosion date: 8/24/2007, DHJPAR0028281. ♂, Guanacaste, Sector Santa Maria, Sendero Canal, 10.76544N -85.28539W 799m., Mariano Pereira coll., food plant: Malvaceae *Mortoniodendron guatemalense*, coll. date: 7/23/2009, parasitoid eclosion date: 8/10/2009, DHJPAR0036354. ♀, Guanacaste, Sector Cacao, Gongora Bananal, 10.88919N -85.47609W 600m., Dunia Garcia coll., food plant unknown, host caterpillar: Gelechiidae, Dichomeridinae, species unknown, coll. date: 6/29/2006, parasitoid eclosion date: 7/24/2006, DHJPAR0028278.

**Figure 31.**
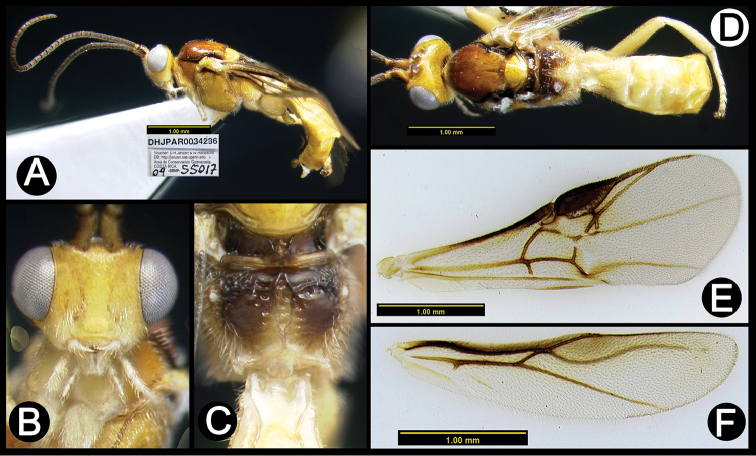
*Lytopylus rogerblancoi* holotype: **A** lateral habitus **B** anterior head **C** propodeum **D** dorsal habitus **E** fore wing **F** hind wing.

#### Lytopylus salvadorlopezi

Taxon classificationAnimaliaHymenopteraBraconidae

Kang sp. n.

http://zoobank.org/6E529BF2-79C8-4B9C-9C3C-DC555133BCCD

Fig. 32

##### Diagnosis.

Scutellar sulcus lacking longitudinal carina; fore wing mostly infuscated; anterior transverse carina of propodeum not reaching the lateral margin; median tergites mostly black.

##### Description.

Holotype: female. Body length 7.5 mm. Fore wing length 7.3 mm. Fore wing mostly infuscated. Scutellar sulcus lacking longitudinal carina. Median areola of propodeum with well-defined margins. Anterior transverse carina of propodeum not reaching the lateral margin. Lateral longitudinal carinae of median tergite 1 well-defined. Median syntergite 2+3 1.2 times longer than wide. Ovipositor length about same length as body.

##### Male.

Unknown.

##### Etymology.

*Lytopylus salvadorlopezi* is named in honor of Salvador López in recognition of his participation in the collaborative development of the ICE-ACG geothermal project of Pailas II, northwestern Costa Rica.

##### Biology.

Reared five times from two species of leaf-tying *Stenoma* (Depressariidae) feeding on *Persea schiedeana* (Lauraceae) in ACG in the rain forest rain forest at 700 m elevation.

##### Type material.

Holotype ♀: Costa Rica, Alajuela, Sector San Cristobal, Quebrada Cementerio, Area de Conservaciόn Guanacaste 10.87124N -85.38749W 700m., Osvaldo Espinoza coll., food plant: Lauraceae *Persea schiedeana*, host caterpillar: Depressariidae, Stenomatinae, *Stenoma* Janzen06, coll. date: 7/6/2009, parasitoid eclosion date: 8/6/2009, DHJPAR0036355. Paratypes: [the following have the same data as the holotype except as indicated] ♀, parasitoid eclosion date: 8/15/2009, DHJPAR0036345. ♀, host caterpillar: *Stenoma* Janzen12, coll. date: 11/19/2009, parasitoid eclosion date: 12/18/2009, DHJPAR0037949.

**Figure 32.**
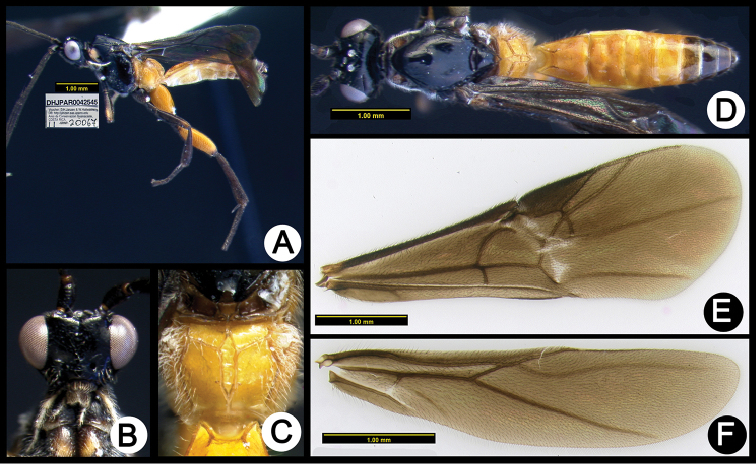
*Lytopylus salvadorlopezi* holotype: **A** lateral habitus **B** anterior head **C** propodeum **D** dorsal habitus **E** fore wing **F** hind wing.

#### Lytopylus sangyeoni

Taxon classificationAnimaliaHymenopteraBraconidae

Kang sp. n.

http://zoobank.org/571E1D60-DF10-4EB9-B070-8291838382F7

Fig. 33

##### Diagnosis.

Fore wing mostly infuscated; hind coxa entirely pale; mesoscutum entirely black; scutellar sulcus with one median longitudinal carina; anterior transverse carina of propodeum reaching the lateral margin; median tergites mostly pale with posterior terga black; median syntergite 2+3 as long as wide.

##### Description.

Holotype: female. Body length 5.5 mm. Fore wing length 5.3 mm. Fore wing mostly infuscated. Scutellar sulcus with one median longitudinal carina. Median areola of propodeum with well-defined margins. Anterior transverse carina of propodeum reaching the lateral margin. Lateral longitudinal carinae of median tergite 1 well-defined. Median syntergite 2+3 as long as wide. Ovipositor longer than metasoma, but shorter than body.

##### Male.

Unknown.

##### Etymology.

Named in honor of Sangyeon Park, father-in-law of the first author.

##### Biology.

Reared one time from elachJanzen01 Janzen847 (Depressariidae) as a leaf-tier feeding on mature leaves of *Senegalia tenuifolia* (Fabaceae) in ACG rain forest at 527 m elevation.

##### Type material.

Holotype ♀: Costa Rica, Alajuela, Sector San Cristobal, Sendero Huerta, Area de Conservaciόn Guanacaste 10.9305N -85.37223W 527m., Carolina Cano coll., food plant: Fabaceae *Senegalia tenuifolia*, host caterpillar: Depressariidae, subfamily unknown, elachJanzen01 Janzen847, coll. date: 11/4/2011, parasitoid eclosion date: 11/21/2011, DHJPAR0046955.

**Figure 33.**
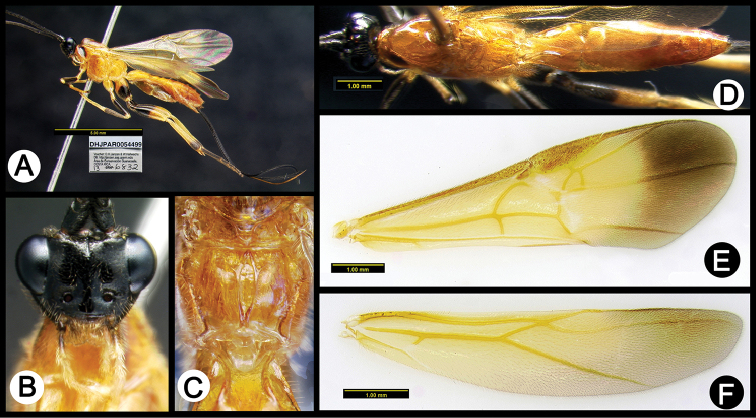
*Lytopylus sangyeoni* holotype: **A** lateral habitus **B** anterior head **C** propodeum **D** dorsal habitus **E** fore wing **F** hind wing.

#### Lytopylus sarahmeierottoae

Taxon classificationAnimaliaHymenopteraBraconidae

Kang sp. n.

http://zoobank.org/8A0864DC-0480-4A42-9052-2E32B869093B

Fig. 34

##### Diagnosis.

Fore wing mostly infuscated; pronotum entirely pale; mesoscutum entirely pale; scutellar sulcus lacking longitudinal carina; anterior transverse carina of propodeum reaching the lateral margin; median tergites mostly melanic, anteriorly white.

##### Description.

Holotype: female. Body length 5.1 mm. Fore wing length 4.8 mm. Fore wing mostly infuscated. Scutellar sulcus lacking longitudinal carina. Median areola of propodeum with well-defined margins. Anterior transverse carina of propodeum reaching the lateral margin. Lateral longitudinal carinae of median tergite 1 well-defined. Median syntergite 2+3 as long as wide. Ovipositor slightly longer than body.

##### Male.

Unknown.

##### Etymology.

Named in honor of Sarah Meierotto, graduate student in the Department of Entomology at the University of Kentucky, for her assistance.

##### Biology.

Reared four times from *Cerconota* Janzen82 (Stenomatinae, Depressariidae) feeding on mature leaves of *Inga micheliana* (Fabaceae) in ACG rain forest at 730 m elevation.

##### Type material.

Holotype ♀: Costa Rica, Alajuela, Sector San Cristobal, Sendero Vivero, Area de Conservaciόn Guanacaste 10.86739N -85.38744W 730m., Elda Araya coll., food plant: Fabaceae *Inga micheliana*, host caterpillar: Depressariidae, Stenomatinae, *Cerconota* Janzen82, coll. date: 12/20/2014, parasitoid eclosion date: 1/13/2015, DHJPAR0056993. Paratypes: [the following have the same data as the holotype except as indicated] 2♀, parasitoid eclosion date: 1/10/2015, DHJPAR0056984, DHJPAR0056991. ♀, parasitoid eclosion date: 1/17/2015, DHJPAR0056992.

**Figure 34.**
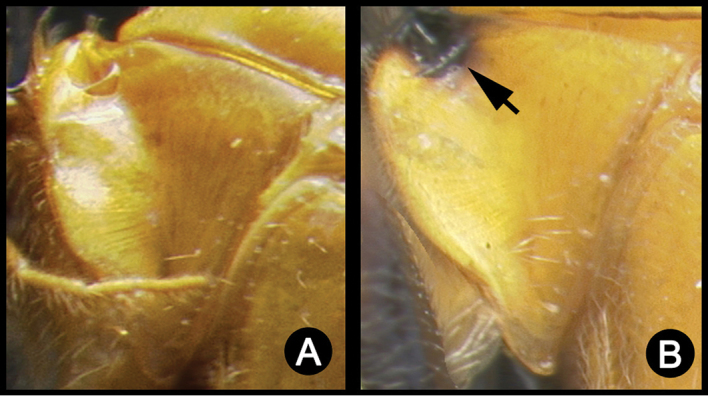
*Lytopylus sarahmeierottoae* holotype: **A** lateral habitus **B** anterior head **C** propodeum **D** dorsal habitus **E** wings.

#### Lytopylus sergiobermudezi

Taxon classificationAnimaliaHymenopteraBraconidae

Kang sp. n.

http://zoobank.org/93CB5271-223B-4600-8D35-75486F6BA0C5

Fig. 35

##### Diagnosis.

Fore wing hyaline; fore wing RS+Ma tubular on more than half its length; anterior transverse carina of propodeum reaching the lateral margin; median tergites mostly pale with posterior terga black.

##### Description.

Holotype: male. Body length 3.9 mm. Fore wing length 3.6 mm. Fore wing hyaline. Fore wing RS+Ma more complete. Scutellar sulcus with four longitudinal carinae. Median areola of propodeum with well-defined margins. Anterior transverse carina of propodeum reaching the lateral margin. Lateral longitudinal carinae of median tergite 1 well-defined. Median syntergite 2+3 1.1 times longer than wide.

##### Female.

Unknown.

##### Variation.

Male mesoscutum varies from less melanic to mostly pale. Male propodeum varies bicolored to entirely pale.

##### Etymology.

*Lytopylus sergiobermudezi* is named in honor of Sergio Bermúdez in recognition of his participation in the collaborative development of the ICE-ACG geothermal project of Pailas II, northwestern Costa Rica.

##### Biology.

Reared two times from *Dichomerus santarosensis* (Dichomeridinae, Gelechiidae) leaf-tier feeding on new foliage of *Quercus oleioides* (Fagaceae) in ACG dry forest at 420 m elevation.

##### Type material.

Holotype ♂: Costa Rica, Guanacaste, Sector Mundo Nuevo, Punta Plancha, Area de Conservaciόn Guanacaste 10.7416N -85.42734W 420m., Mariano Pereira coll., food plant: Fagaceae *Quercus oleoides*, host caterpillar: Gelechiidae, Dichomeridinae, *Dichomeris santarosensis*, coll. date: 1/5/2009, parasitoid eclosion date: 1/19/2009, DHJPAR0034286. Paratype: [the following has the same data as the holotype except as indicated] DHJPAR0030601.

**Figure 35.**
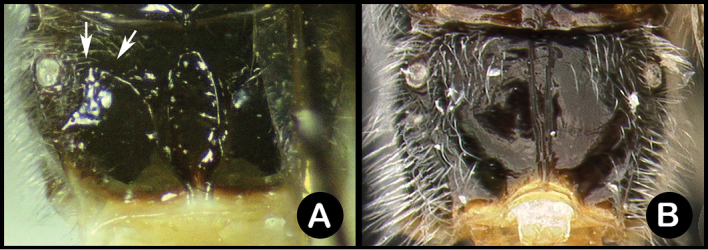
*Lytopylus sergiobermudezi* holotype: **A** lateral habitus, B. anterior head **C** propodeum **D** dorsal habitus **E** fore wing **F** hind wing.

#### Lytopylus sigifredomarini

Taxon classificationAnimaliaHymenopteraBraconidae

Kang sp. n.

http://zoobank.org/5E10353D-E093-4A12-886F-C92596DEE86E

Fig. 36

##### Diagnosis.

Fore wing mostly infuscated; hind coxa entirely pale; mesoscutum entirely black; scutellar sulcus lacking longitudinal carina; anterior transverse carina of propodeum reaching the lateral margin.

##### Description.

Holotype: male. Body length 5.2 mm. Fore wing length 4.8 mm. Fore wing mostly infuscated. Scutellar sulcus lacking longitudinal carina. Median areola of propodeum with well-defined margins. Anterior transverse carina of propodeum reaching the lateral margin. Lateral longitudinal carinae of median tergite 1 well-defined. Median syntergite 2+3 1.2 times longer than wide.

##### Female.

Unknown.

##### Etymology.

*Lytopylus sigifredomarini* is named in honor of Sigifredo Marín in recognition of his participation in the collaborative development of the ICE-ACG geothermal project of Pailas II, northwestern Costa Rica.

##### Biology.

Reared three times from *Antaeotricha* Janzen224 (Stenomatinae, Depressariidae) feeding on mature leaves of *Hirtella media* (Chrysobalanaceae) in ACG rain forest at 410 to 620 m elevation.

##### Type material.

Holotype ♂: Costa Rica, Guanacaste, Sector Del Oro, Tangelo, Area de Conservaciόn Guanacaste 11.01823N -85.45024W 410m., Elieth Cantillano coll., food plant: Chrysobalanaceae *Hirtella triandra*, host caterpillar: Depressariidae, Stenomatinae, *Antaeotricha* Janzen224, coll. date: 1/6/2011, parasitoid eclosion date: 1/31/2011, DHJPAR0042545. Paratypes: [the following have the same data as the holotype except as indicated] ♂, Bosque Aguirre, 11.0006N -85.438W 620m., Roster Moraga coll., coll. date: 5/7/2010, parasitoid eclosion date: 5/31/2010, DHJPAR0040328. ♂, Sector Pitilla, Coneja, 11.01525N -85.39766W 415m., Dinia Martinez coll., food plant: *Hirtella media*, coll. date: 5/6/2013, parasitoid eclosion date: 5/27/2013, DHJPAR0052899.

**Figure 36.**
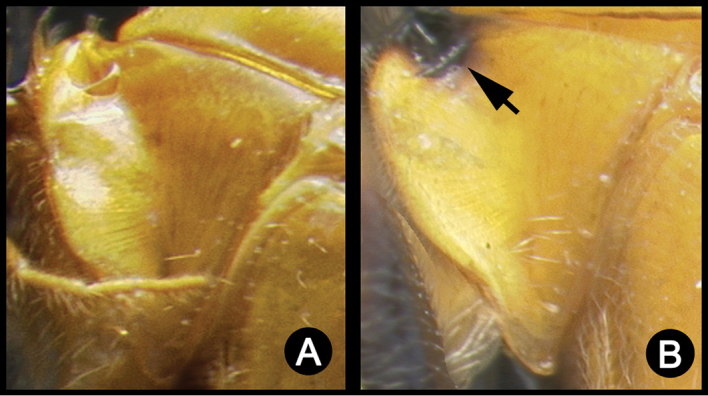
*Lytopylus sigifredomarini* holotype: **A** lateral habitus **B** anterior head **C** propodeum **D** dorsal habitus **E** fore wing **F** hind wing.

#### Lytopylus youngcheae

Taxon classificationAnimaliaHymenopteraBraconidae

Kang sp. n.

http://zoobank.org/6890246F-A78D-4AA9-AE26-F4322390B5DE

Fig. 37

##### Diagnosis.

Fore wing with one black band; vertex of head entirely melanic.

##### Description.

Holotype: female. Body length 8.9 mm. Fore wing length 8.1 mm. Fore wing with one black band. Scutellar sulcus with one median longitudinal carina. Anterior transverse carina of propodeum not reaching the lateral margin. Median areola of propodeum with well-defined margins. Lateral longitudinal carinae of median tergite 1 well-defined. Median syntergite 2+3 1.5 times longer than wide. Ovipositor slightly longer than body.

##### Males.

Body length usually shorter than holotype. Hind femur varies from mostly pale to mostly melanic.

##### Etymology.

Named in honor of Youngche Choi, mother of the first author.

##### Biology.

Reared five times from two species of stenomatine Depressariidae leaf-tiers feeding on mature leaves of *Calophyllum brasiliense* (Calophyllaceae) in ACG rain forest at 540 to 740 m elevation.

##### Type material.

Holotype ♀: Costa Rica, Alajuela, Sector San Cristobal, Cementerio Viejo, Area de Conservaciόn Guanacaste 10.88111N -85.38889W 570m., Carolina Cano coll., food plant: Calophyllaceae *Calophyllum brasiliense*, host caterpillar: Depressariidae, Stenomatinae, *Cerconota* Janzen140, coll. date: 11/27/2013, parasitoid eclosion date: 12/31/2013, DHJPAR0054499. Paratypes: [the following have the same data as the holotype except as indicated] ♀, Guanacaste, Sector Pitilla, Sendero Memos, 10.98171N -85.42785W 740m., Elieth Cantillano coll., coll. date: 4/27/2007 parasitoid eclosion date: 5/27/2007, DHJPAR0021132. ♂, Finca San Gabriel, 10.87766N -85.39343W 645m., host caterpillar: *Antaeotricha* Janzen134, coll. date: 4/1/2013, parasitoid eclosion date: 4/30/2013, DHJPAR0052197. ♀, Tajo Angeles, 10.86472N -85.41531W 540m., Elda Araya coll., host caterpillar: *Antaeotricha* Janzen134, coll. date: 12/31/2010, parasitoid eclosion date: 1/24/2011, DHJPAR0041586. [same as previous except as eclosion date] ♂, parasitoid eclosion date:1/25/2011, DHJPAR0041585.

**Figure 37.**
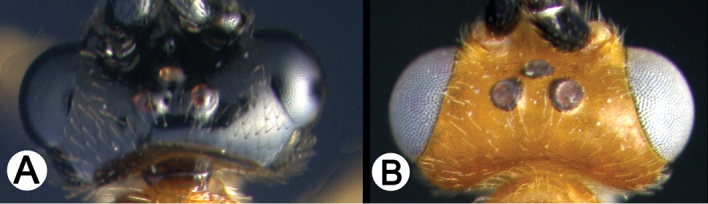
*Lytopylus youngcheae* holotype: **A** lateral habitus **B** anterior head **C** propodeum **D** dorsal habitus **E** fore wing **F** hind wing.

## Supplementary Material

XML Treatment for Lytopylus

XML Treatment for Lytopylus alejandromasisi

XML Treatment for Lytopylus alfredomainieri

XML Treatment for Lytopylus anamariamongeae

XML Treatment for Lytopylus angelagonzalezae

XML Treatment for Lytopylus cesarmorai

XML Treatment for Lytopylus chrysokeras

XML Treatment for Lytopylus eddysanchezi

XML Treatment for Lytopylus eliethcantillanoae

XML Treatment for Lytopylus ericchapmani

XML Treatment for Lytopylus gahyunae

XML Treatment for Lytopylus gisukae

XML Treatment for Lytopylus guillermopereirai

XML Treatment for Lytopylus gustavoindunii

XML Treatment for Lytopylus hartmanguidoi

XML Treatment for Lytopylus hernanbravoi

XML Treatment for Lytopylus hokwoni

XML Treatment for Lytopylus ivanniasandovalae

XML Treatment for Lytopylus johanvalerioi

XML Treatment for Lytopylus josecortesi

XML Treatment for Lytopylus luisgaritai

XML Treatment for Lytopylus mariamartachavarriae

XML Treatment for Lytopylus miguelviquezi

XML Treatment for Lytopylus motohasegawai

XML Treatment for Lytopylus okchunae

XML Treatment for Lytopylus pablocobbi

XML Treatment for Lytopylus robertofernandezi

XML Treatment for Lytopylus rogerblancoi

XML Treatment for Lytopylus salvadorlopezi

XML Treatment for Lytopylus sangyeoni

XML Treatment for Lytopylus sarahmeierottoae

XML Treatment for Lytopylus sergiobermudezi

XML Treatment for Lytopylus sigifredomarini

XML Treatment for Lytopylus youngcheae

## Supplementary materials

Supplementary material 1 Interactive key, DELTA data matrix, and images for the revision of the species of *Lytopylus* from Area de Conservación Guanacaste, northwestern Costa Rica (Hymenoptera, Braconidae, Agathidinae) Data type: interactive key This is an open access article distributed under the terms of the Creative Commons Attribution License (CC BY 4.0), which permits unrestricted use, distribution, and reproduction in any medium, provided the original author and source are credited.Ilgoo Kang, Eric G. Chapman, Daniel H. Janzen, Winnie Hallwachs, Tanya Dapkey, Smith M. Alex, Michael J. Sharkey
