# Supplementary figures and images for: Revision of the species of Lytopylus from Area de Conservación Guanacaste, northwestern Costa Rica (Hymenoptera, Braconidae, Agathidinae)
Source: Zookeys. 2017 Dec 12;(721):93–158. doi: 10.3897/zookeys.721.20287 (PMC5740408; doi:10.3897/zookeys.721.20287)

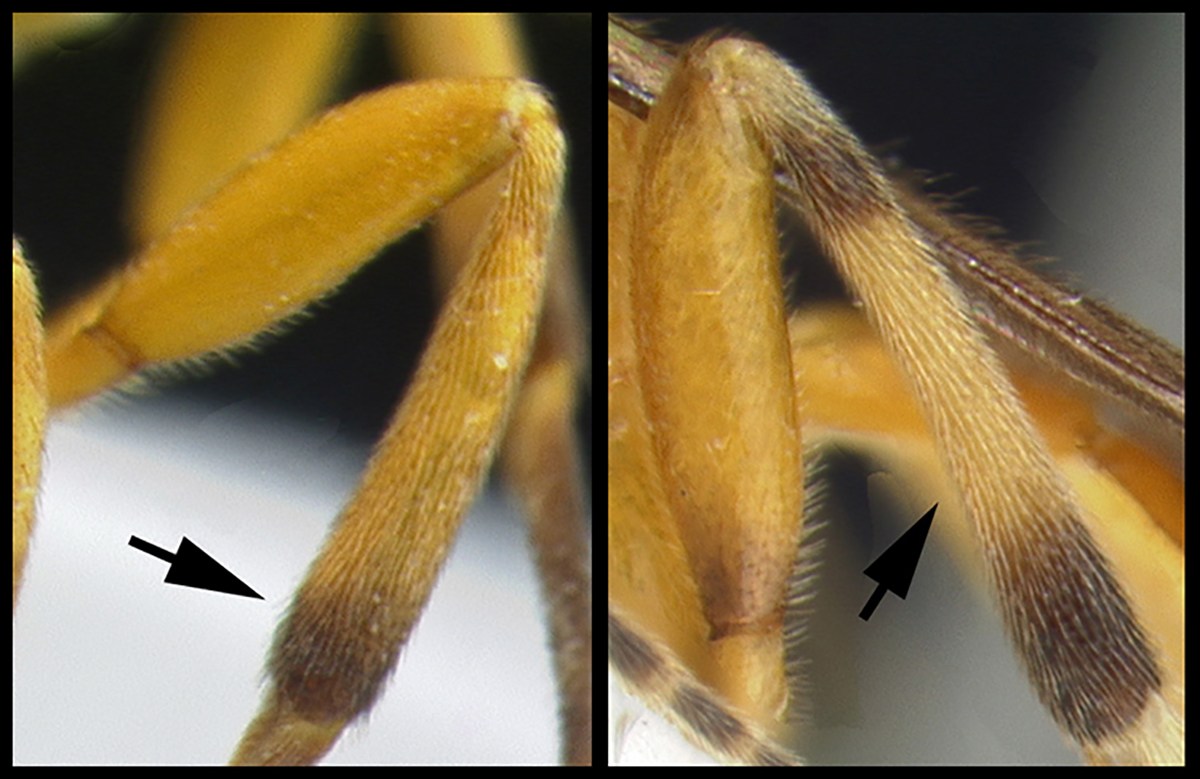

Supplement: Supplementary material 1 — Interactive key, DELTA data matrix, and images for the revision of the species of Lytopylus from Area de Conservación Guanacaste, northwestern Costa Rica (Hymenoptera, Braconidae, Agathidinae) [file zookeys-721-093-s001.zip › Revised ACG Lytopylus Interactive key/Revised Lytopylus Interactive key/images/10hindtibia.jpg]

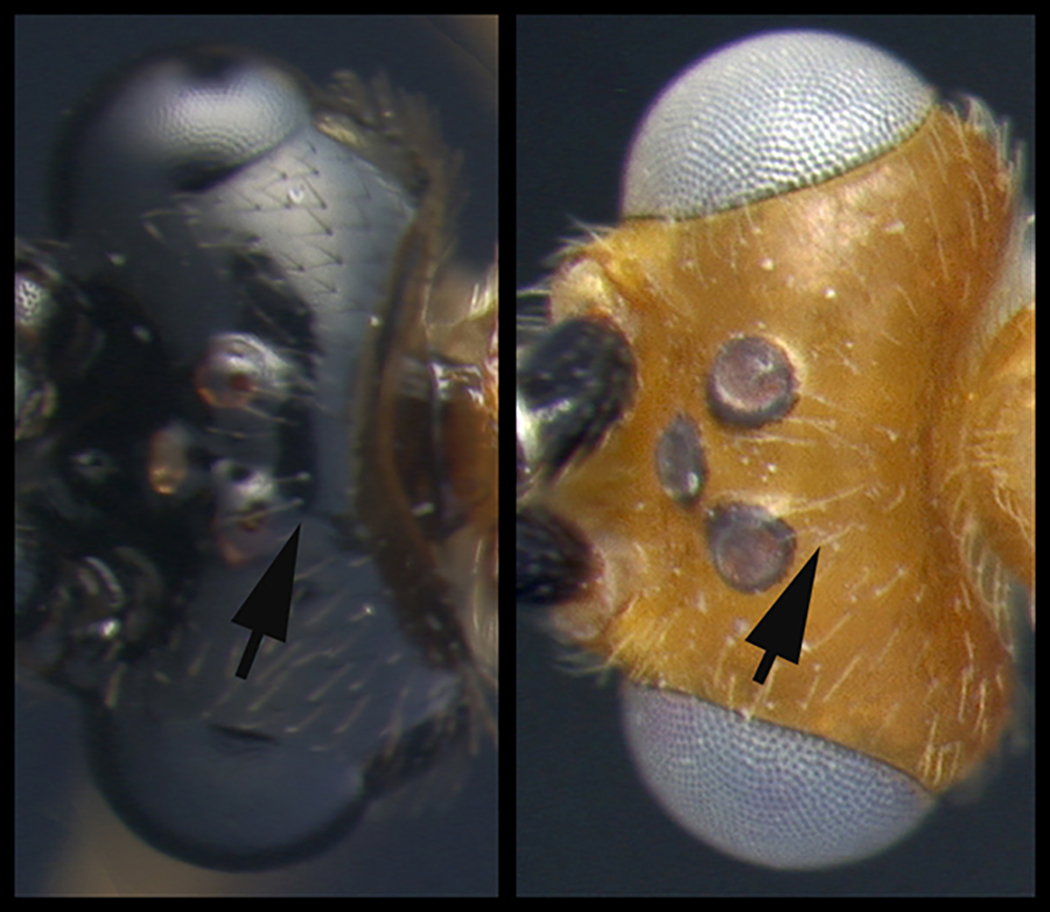

Supplement: Supplementary material 1 — Interactive key, DELTA data matrix, and images for the revision of the species of Lytopylus from Area de Conservación Guanacaste, northwestern Costa Rica (Hymenoptera, Braconidae, Agathidinae) [file zookeys-721-093-s001.zip › Revised ACG Lytopylus Interactive key/Revised Lytopylus Interactive key/images/11vertexofhead.jpg]

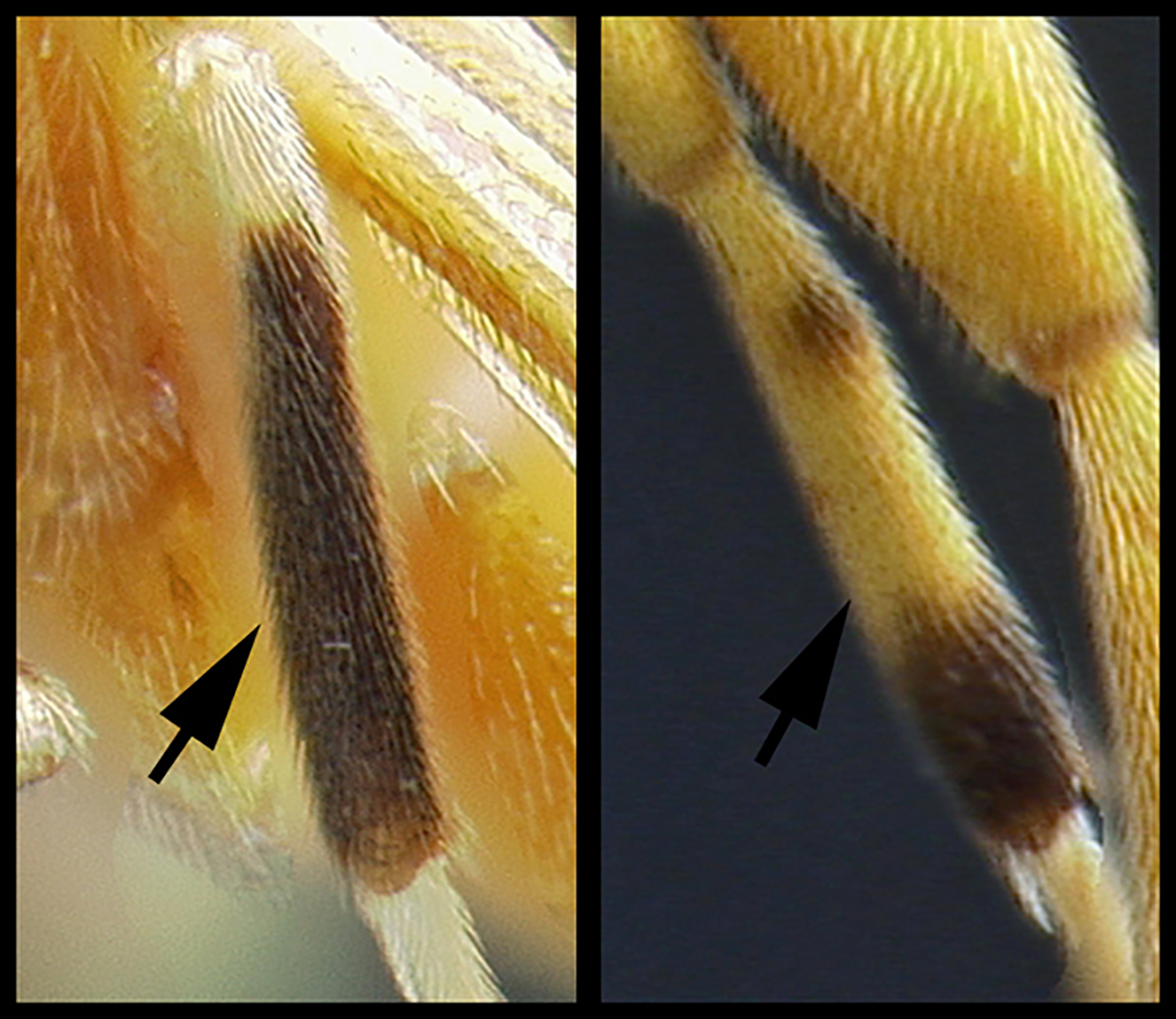

Supplement: Supplementary material 1 — Interactive key, DELTA data matrix, and images for the revision of the species of Lytopylus from Area de Conservación Guanacaste, northwestern Costa Rica (Hymenoptera, Braconidae, Agathidinae) [file zookeys-721-093-s001.zip › Revised ACG Lytopylus Interactive key/Revised Lytopylus Interactive key/images/12midtibia.jpg]

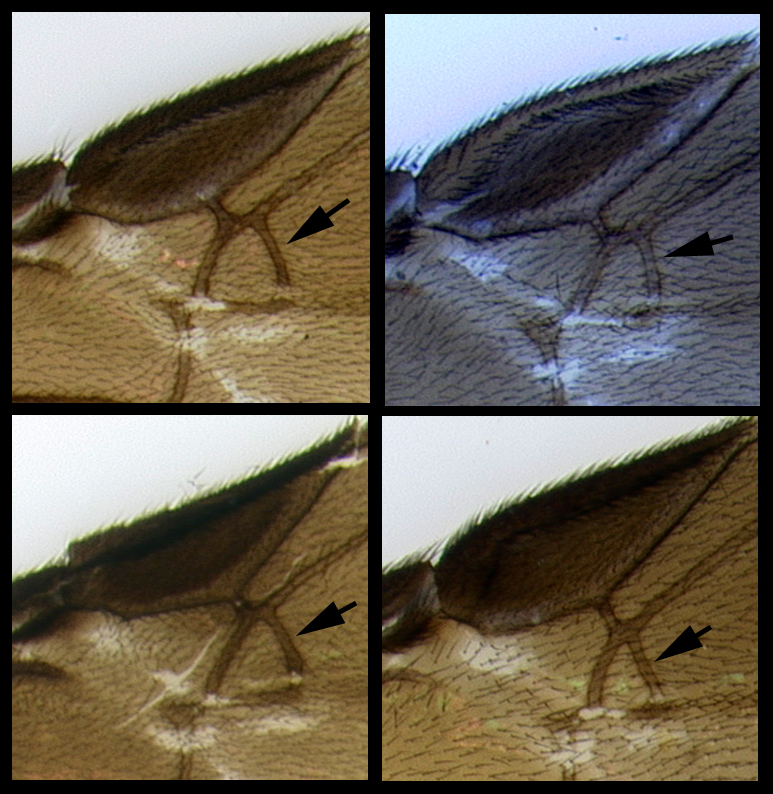

Supplement: Supplementary material 1 — Interactive key, DELTA data matrix, and images for the revision of the species of Lytopylus from Area de Conservación Guanacaste, northwestern Costa Rica (Hymenoptera, Braconidae, Agathidinae) [file zookeys-721-093-s001.zip › Revised ACG Lytopylus Interactive key/Revised Lytopylus Interactive key/images/13secondsubmarginalforewing.jpg]

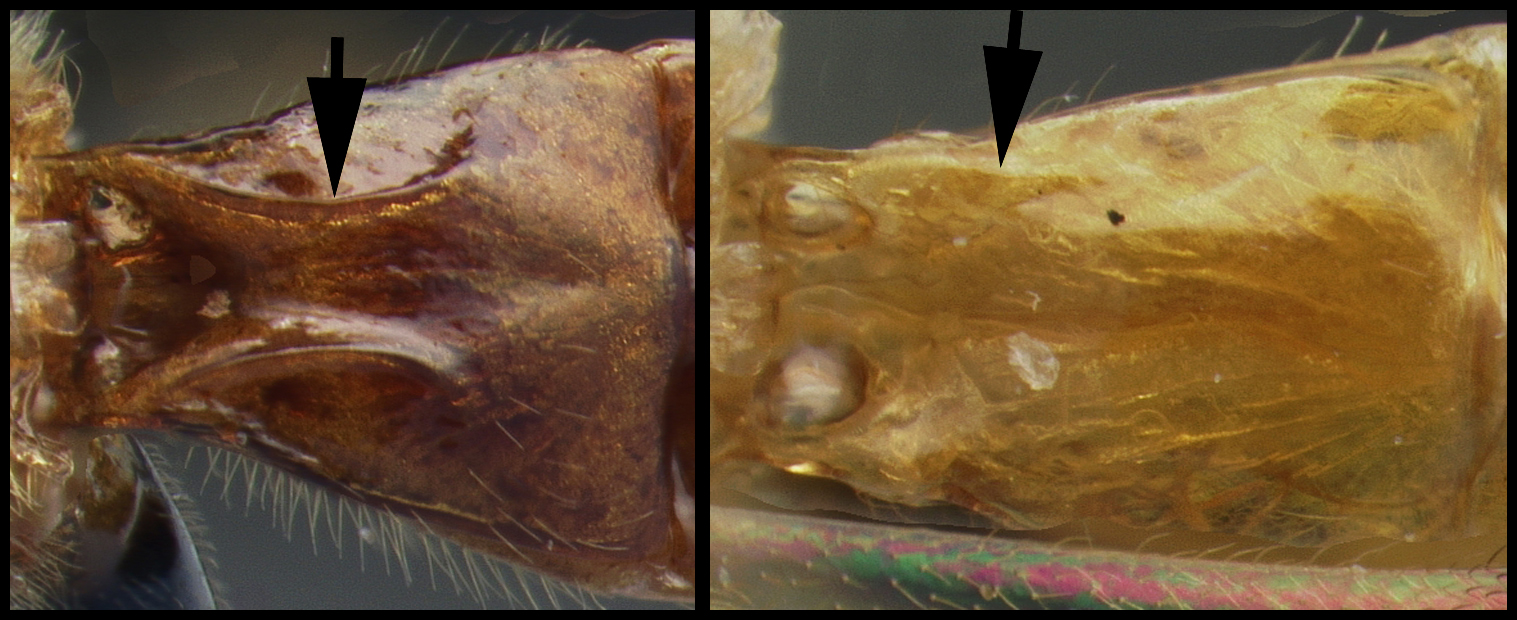

Supplement: Supplementary material 1 — Interactive key, DELTA data matrix, and images for the revision of the species of Lytopylus from Area de Conservación Guanacaste, northwestern Costa Rica (Hymenoptera, Braconidae, Agathidinae) [file zookeys-721-093-s001.zip › Revised ACG Lytopylus Interactive key/Revised Lytopylus Interactive key/images/14laterallongitudinalcarinae.jpg]

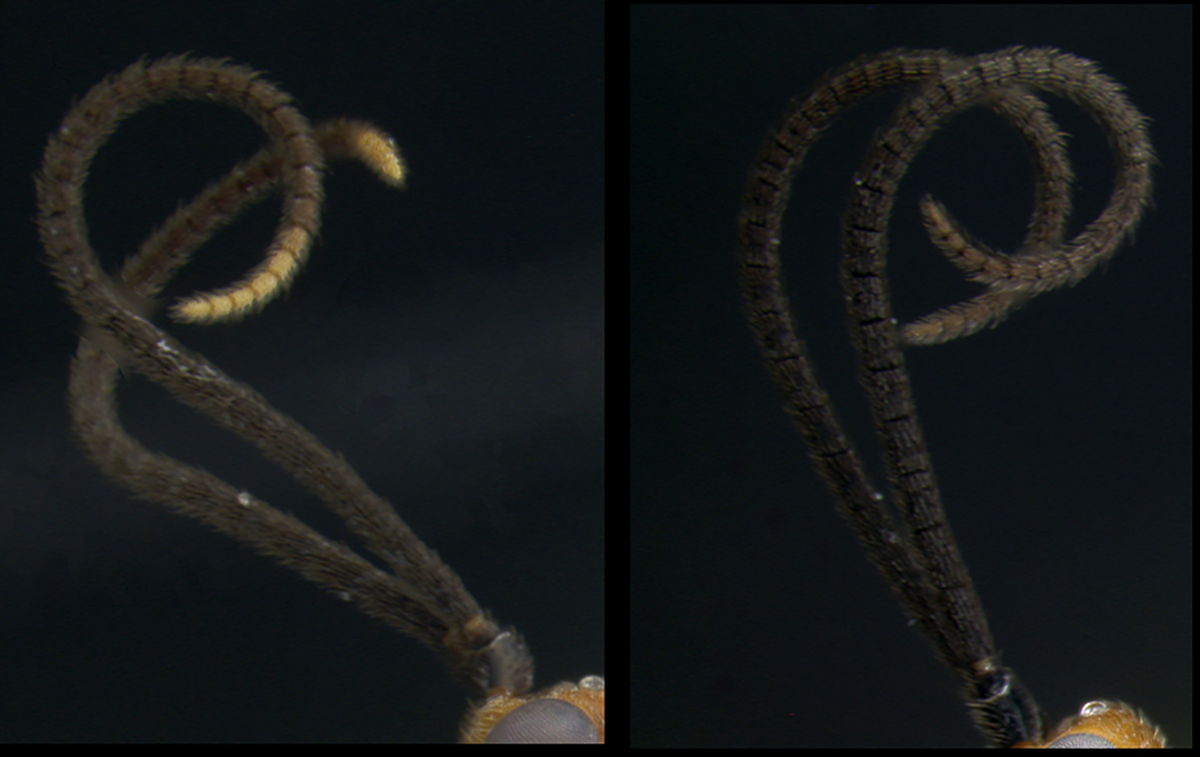

Supplement: Supplementary material 1 — Interactive key, DELTA data matrix, and images for the revision of the species of Lytopylus from Area de Conservación Guanacaste, northwestern Costa Rica (Hymenoptera, Braconidae, Agathidinae) [file zookeys-721-093-s001.zip › Revised ACG Lytopylus Interactive key/Revised Lytopylus Interactive key/images/15apicalflagellomeres.jpg]

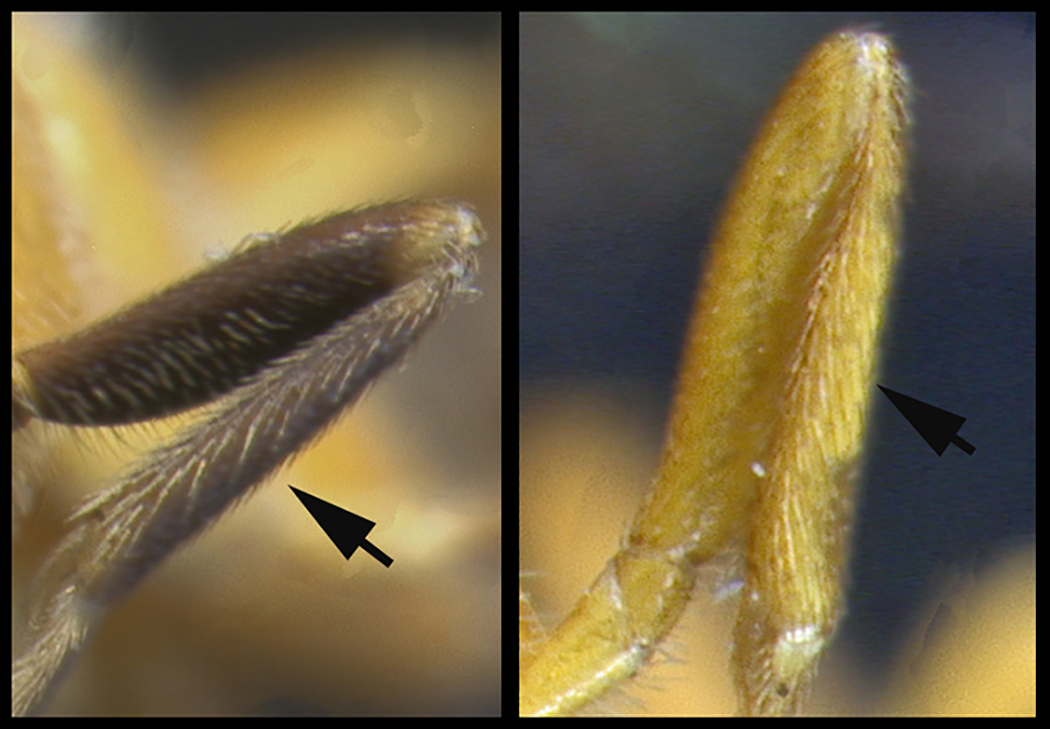

Supplement: Supplementary material 1 — Interactive key, DELTA data matrix, and images for the revision of the species of Lytopylus from Area de Conservación Guanacaste, northwestern Costa Rica (Hymenoptera, Braconidae, Agathidinae) [file zookeys-721-093-s001.zip › Revised ACG Lytopylus Interactive key/Revised Lytopylus Interactive key/images/16foretibia.jpg]

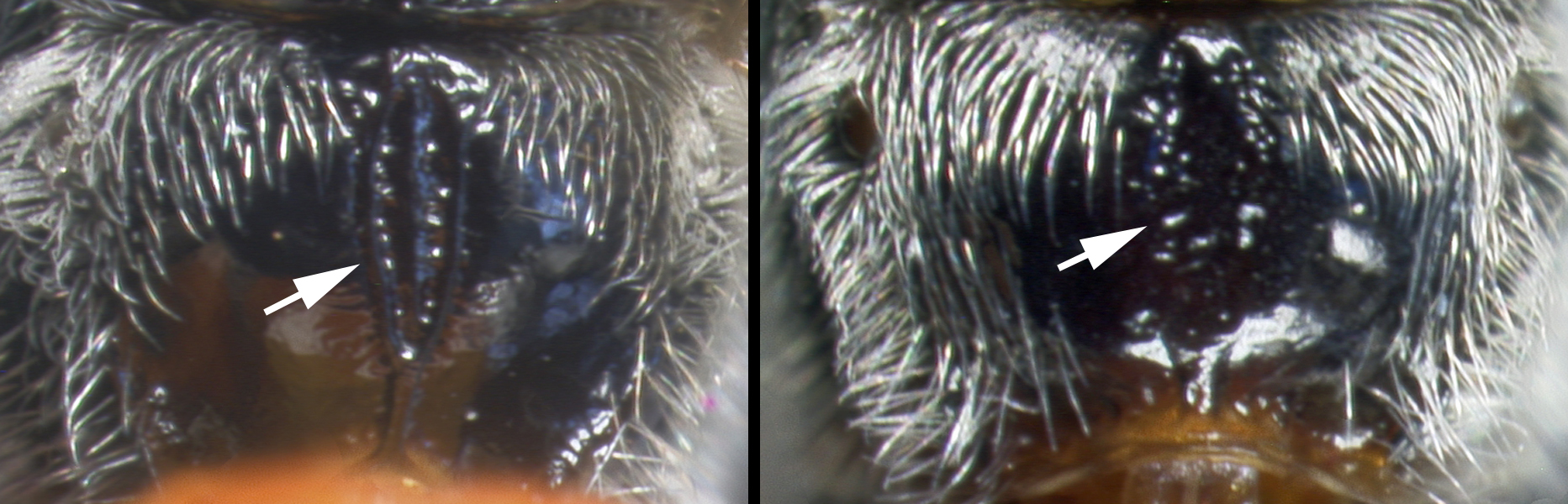

Supplement: Supplementary material 1 — Interactive key, DELTA data matrix, and images for the revision of the species of Lytopylus from Area de Conservación Guanacaste, northwestern Costa Rica (Hymenoptera, Braconidae, Agathidinae) [file zookeys-721-093-s001.zip › Revised ACG Lytopylus Interactive key/Revised Lytopylus Interactive key/images/17medianareolaofpropodeum.jpg]

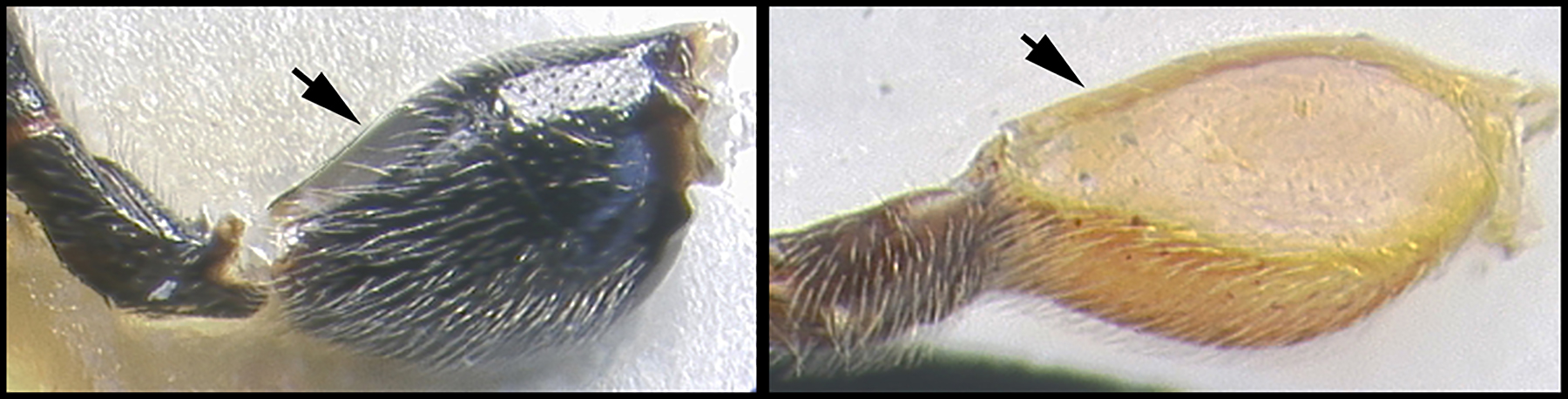

Supplement: Supplementary material 1 — Interactive key, DELTA data matrix, and images for the revision of the species of Lytopylus from Area de Conservación Guanacaste, northwestern Costa Rica (Hymenoptera, Braconidae, Agathidinae) [file zookeys-721-093-s001.zip › Revised ACG Lytopylus Interactive key/Revised Lytopylus Interactive key/images/18hindcoxa.jpg]

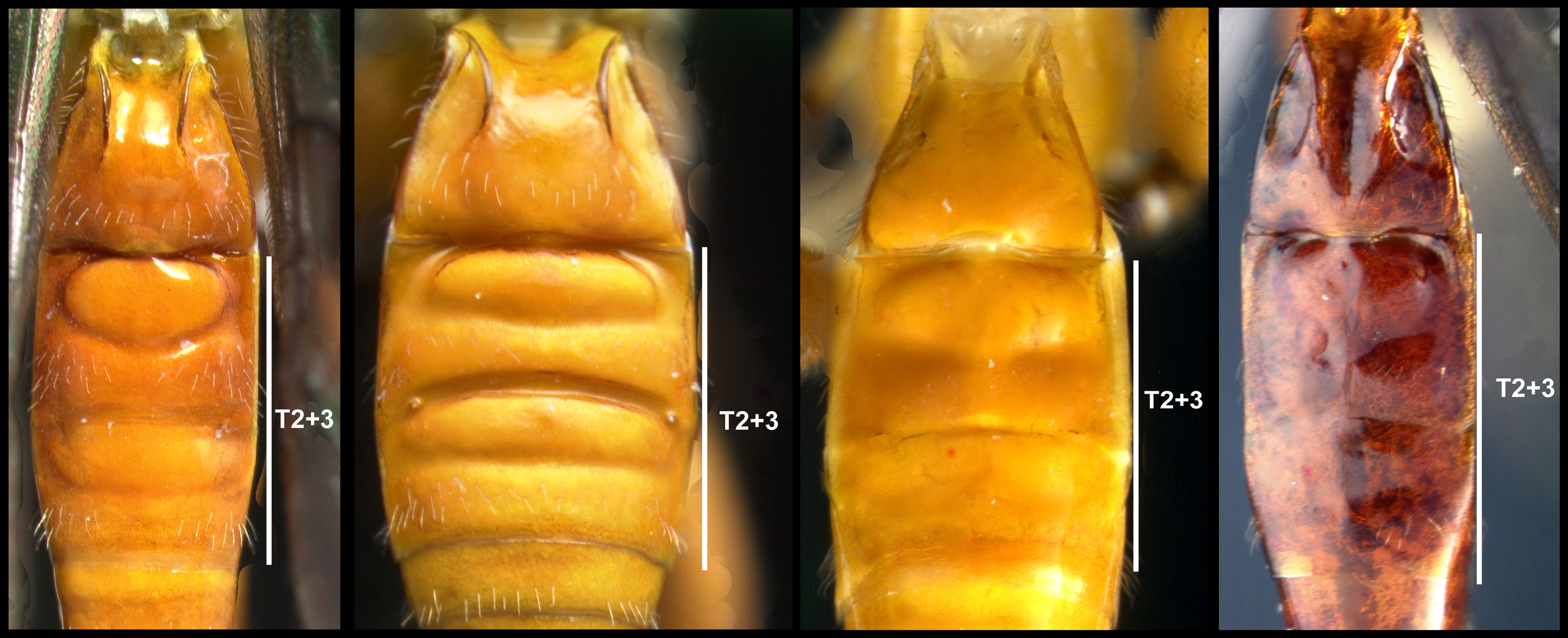

Supplement: Supplementary material 1 — Interactive key, DELTA data matrix, and images for the revision of the species of Lytopylus from Area de Conservación Guanacaste, northwestern Costa Rica (Hymenoptera, Braconidae, Agathidinae) [file zookeys-721-093-s001.zip › Revised ACG Lytopylus Interactive key/Revised Lytopylus Interactive key/images/19mediansyntergite.jpg]

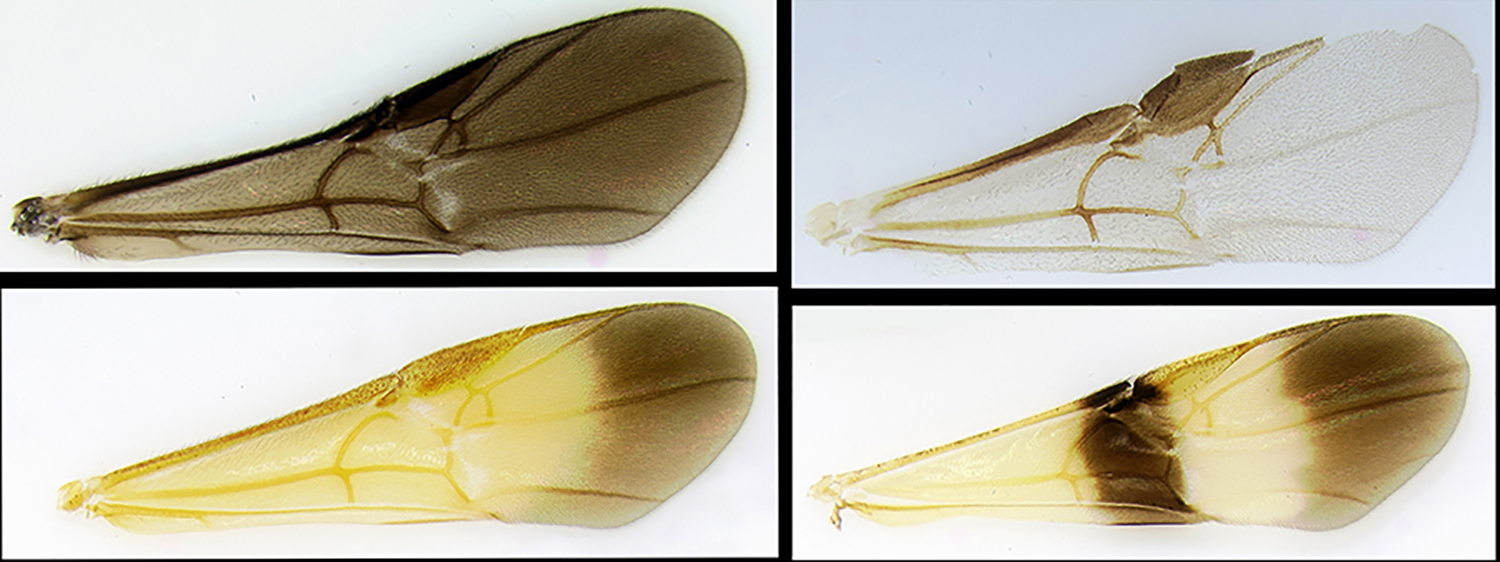

Supplement: Supplementary material 1 — Interactive key, DELTA data matrix, and images for the revision of the species of Lytopylus from Area de Conservación Guanacaste, northwestern Costa Rica (Hymenoptera, Braconidae, Agathidinae) [file zookeys-721-093-s001.zip › Revised ACG Lytopylus Interactive key/Revised Lytopylus Interactive key/images/1forewing.jpg]

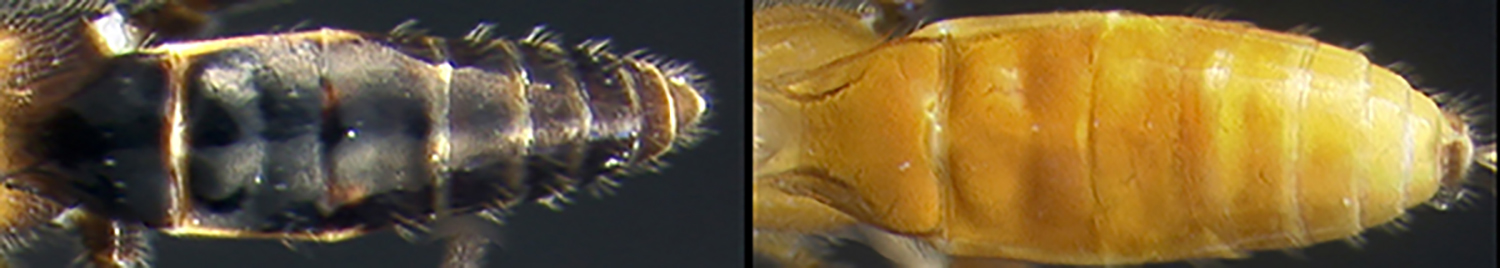

Supplement: Supplementary material 1 — Interactive key, DELTA data matrix, and images for the revision of the species of Lytopylus from Area de Conservación Guanacaste, northwestern Costa Rica (Hymenoptera, Braconidae, Agathidinae) [file zookeys-721-093-s001.zip › Revised ACG Lytopylus Interactive key/Revised Lytopylus Interactive key/images/2mediantergites.jpg]

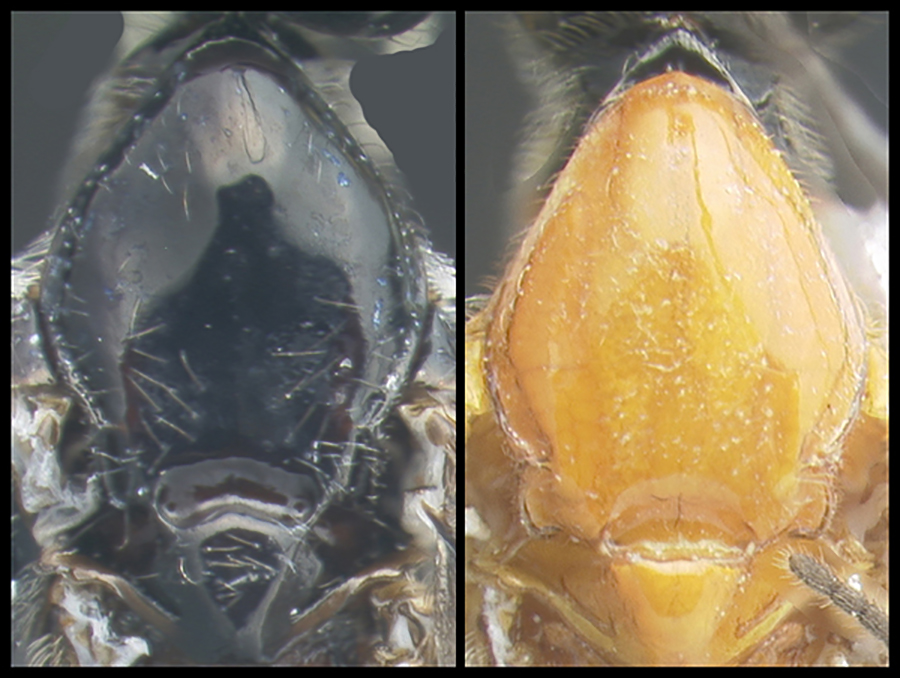

Supplement: Supplementary material 1 — Interactive key, DELTA data matrix, and images for the revision of the species of Lytopylus from Area de Conservación Guanacaste, northwestern Costa Rica (Hymenoptera, Braconidae, Agathidinae) [file zookeys-721-093-s001.zip › Revised ACG Lytopylus Interactive key/Revised Lytopylus Interactive key/images/3mesoscutum.jpg]

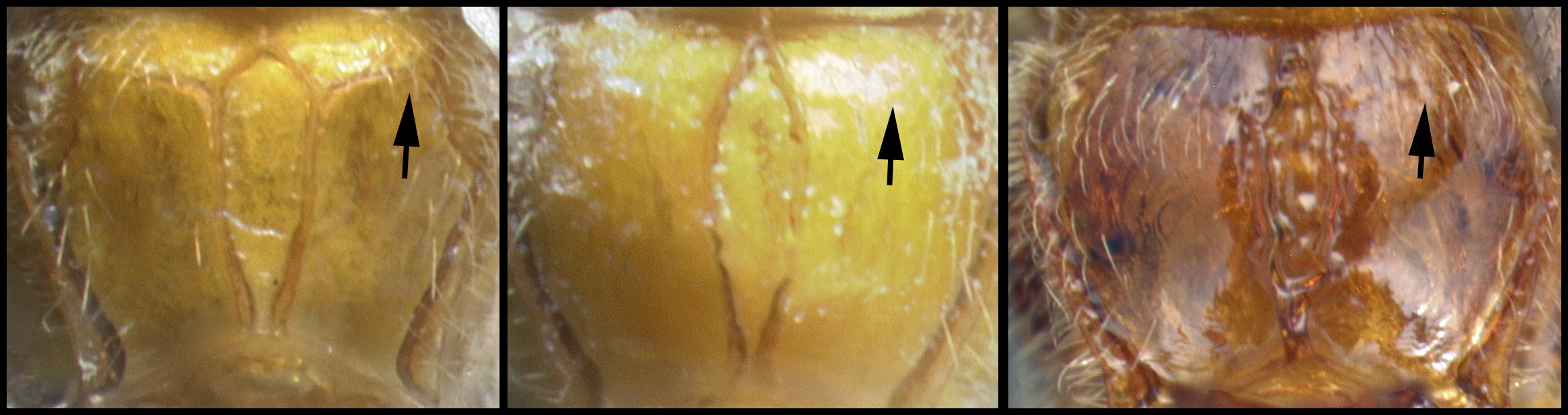

Supplement: Supplementary material 1 — Interactive key, DELTA data matrix, and images for the revision of the species of Lytopylus from Area de Conservación Guanacaste, northwestern Costa Rica (Hymenoptera, Braconidae, Agathidinae) [file zookeys-721-093-s001.zip › Revised ACG Lytopylus Interactive key/Revised Lytopylus Interactive key/images/4anteriortransversecarinae.jpg]

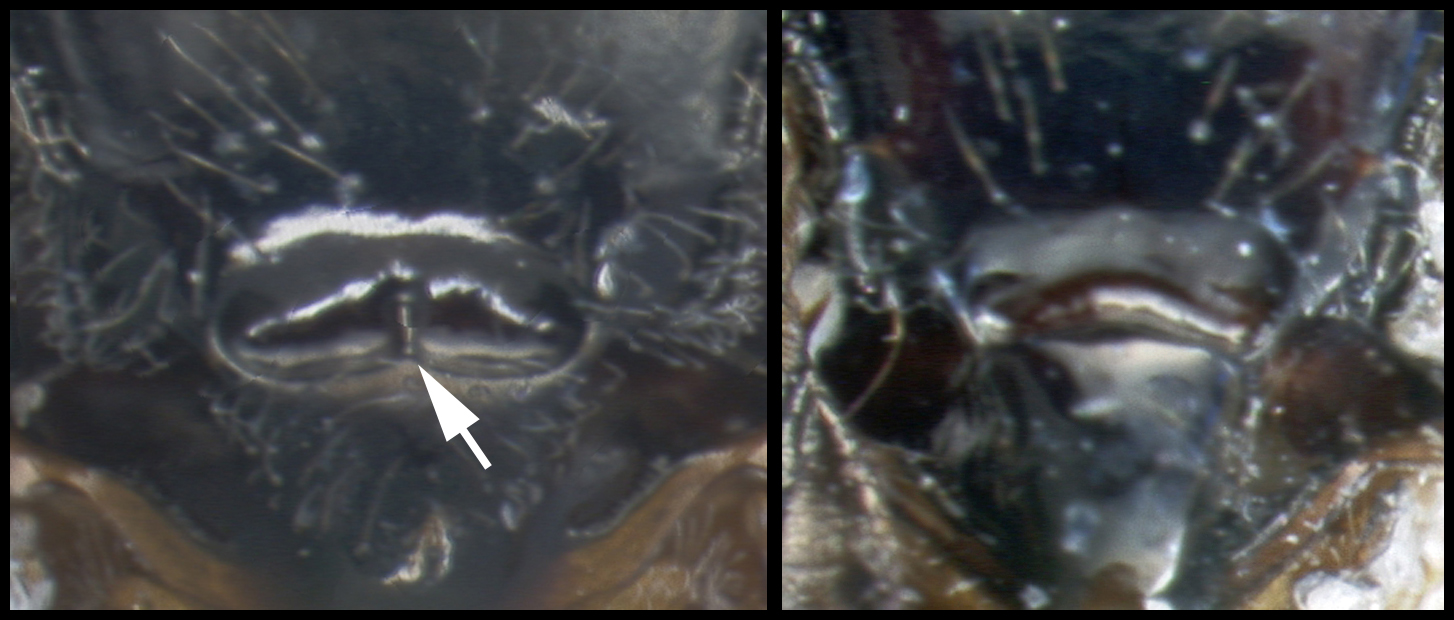

Supplement: Supplementary material 1 — Interactive key, DELTA data matrix, and images for the revision of the species of Lytopylus from Area de Conservación Guanacaste, northwestern Costa Rica (Hymenoptera, Braconidae, Agathidinae) [file zookeys-721-093-s001.zip › Revised ACG Lytopylus Interactive key/Revised Lytopylus Interactive key/images/5scutellarsulcus.jpg]

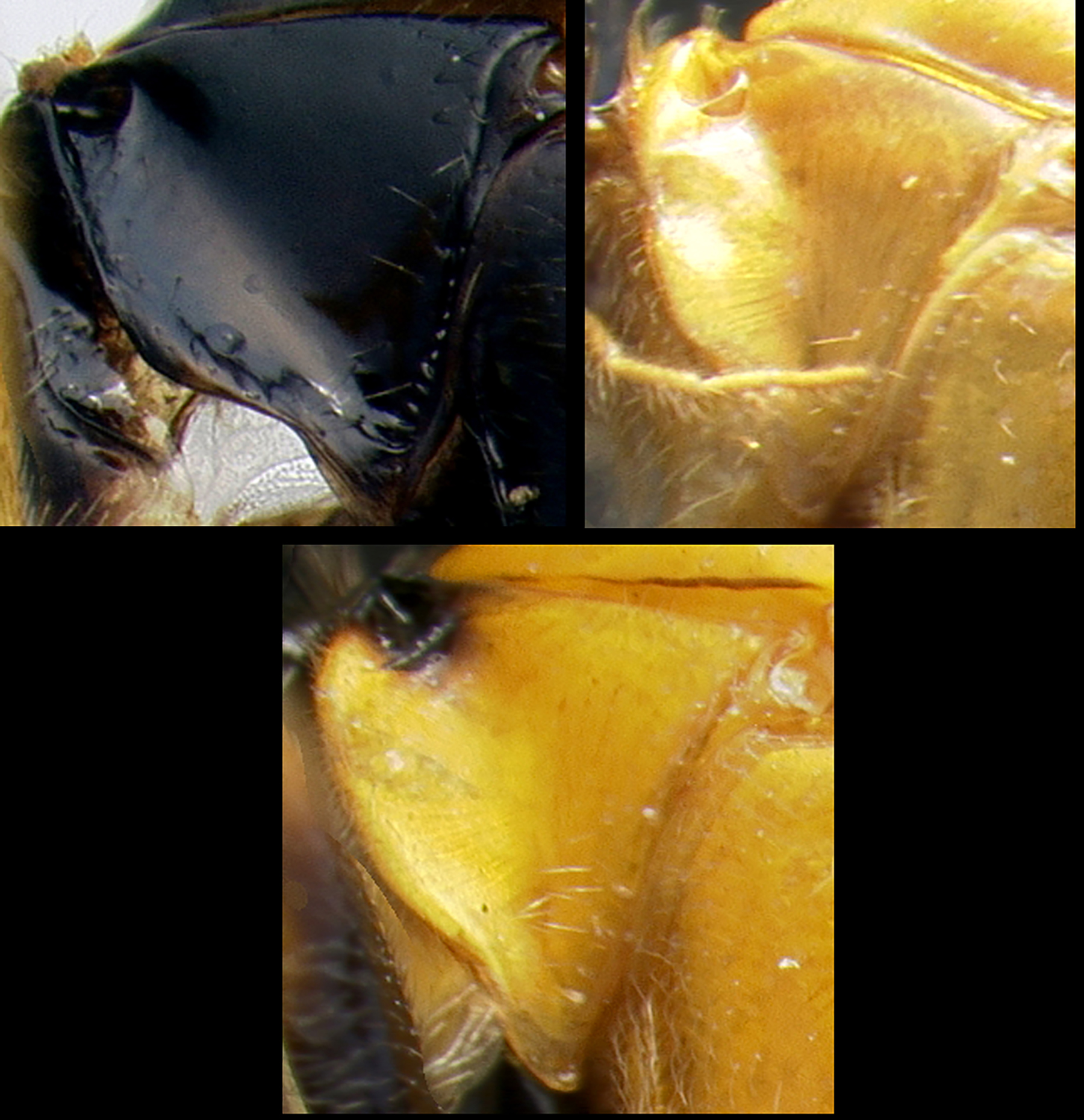

Supplement: Supplementary material 1 — Interactive key, DELTA data matrix, and images for the revision of the species of Lytopylus from Area de Conservación Guanacaste, northwestern Costa Rica (Hymenoptera, Braconidae, Agathidinae) [file zookeys-721-093-s001.zip › Revised ACG Lytopylus Interactive key/Revised Lytopylus Interactive key/images/6pronotumcolor.jpg]

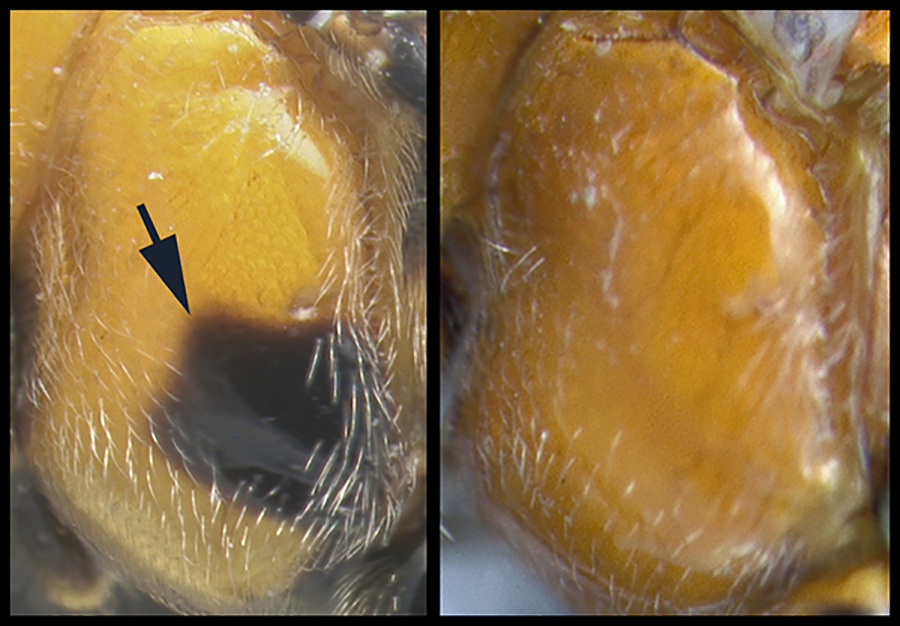

Supplement: Supplementary material 1 — Interactive key, DELTA data matrix, and images for the revision of the species of Lytopylus from Area de Conservación Guanacaste, northwestern Costa Rica (Hymenoptera, Braconidae, Agathidinae) [file zookeys-721-093-s001.zip › Revised ACG Lytopylus Interactive key/Revised Lytopylus Interactive key/images/7mesopleuroncolor.jpg]

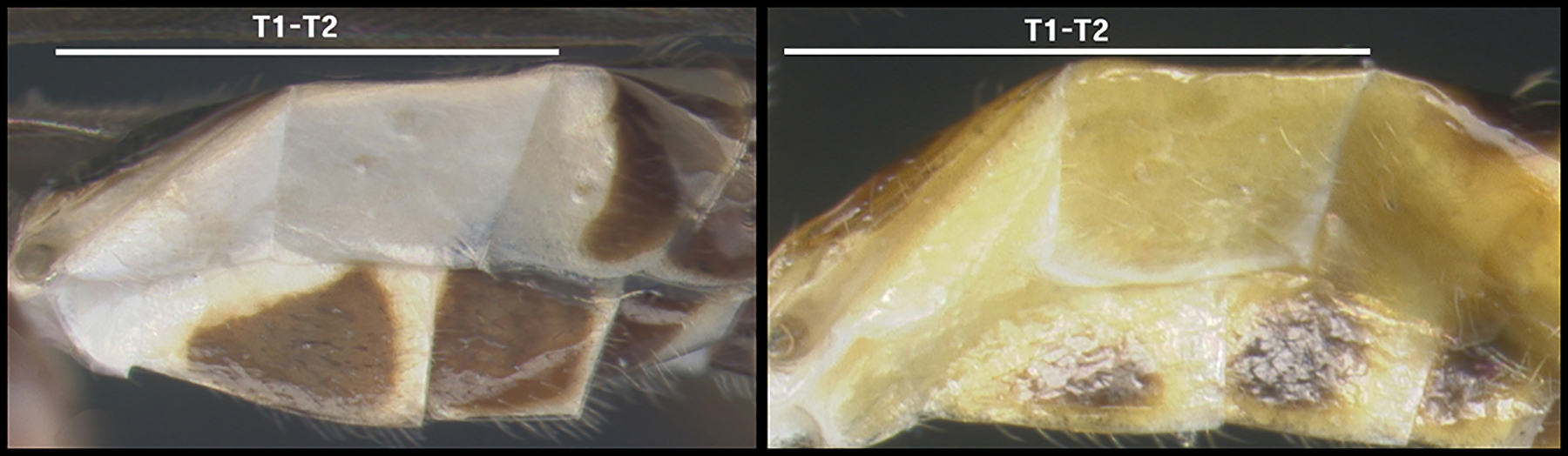

Supplement: Supplementary material 1 — Interactive key, DELTA data matrix, and images for the revision of the species of Lytopylus from Area de Conservación Guanacaste, northwestern Costa Rica (Hymenoptera, Braconidae, Agathidinae) [file zookeys-721-093-s001.zip › Revised ACG Lytopylus Interactive key/Revised Lytopylus Interactive key/images/8lateraltergites.jpg]

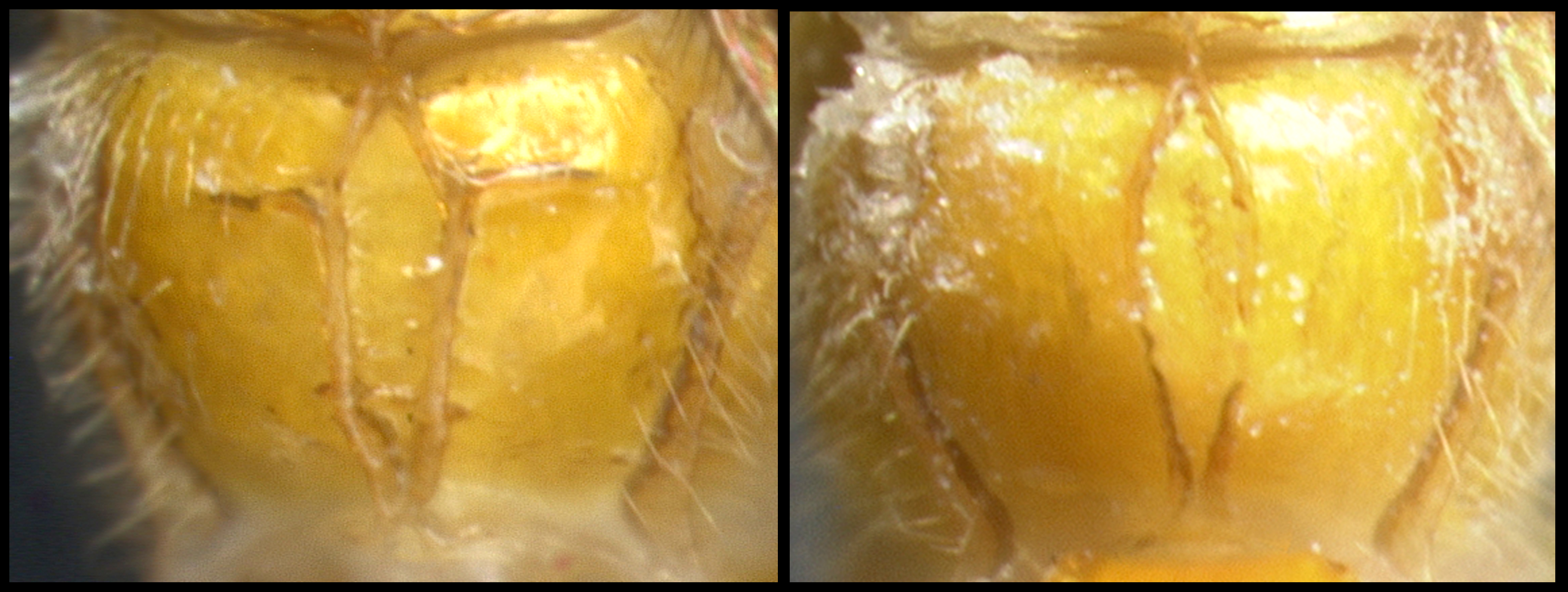

Supplement: Supplementary material 1 — Interactive key, DELTA data matrix, and images for the revision of the species of Lytopylus from Area de Conservación Guanacaste, northwestern Costa Rica (Hymenoptera, Braconidae, Agathidinae) [file zookeys-721-093-s001.zip › Revised ACG Lytopylus Interactive key/Revised Lytopylus Interactive key/images/8medianareola.jpg]

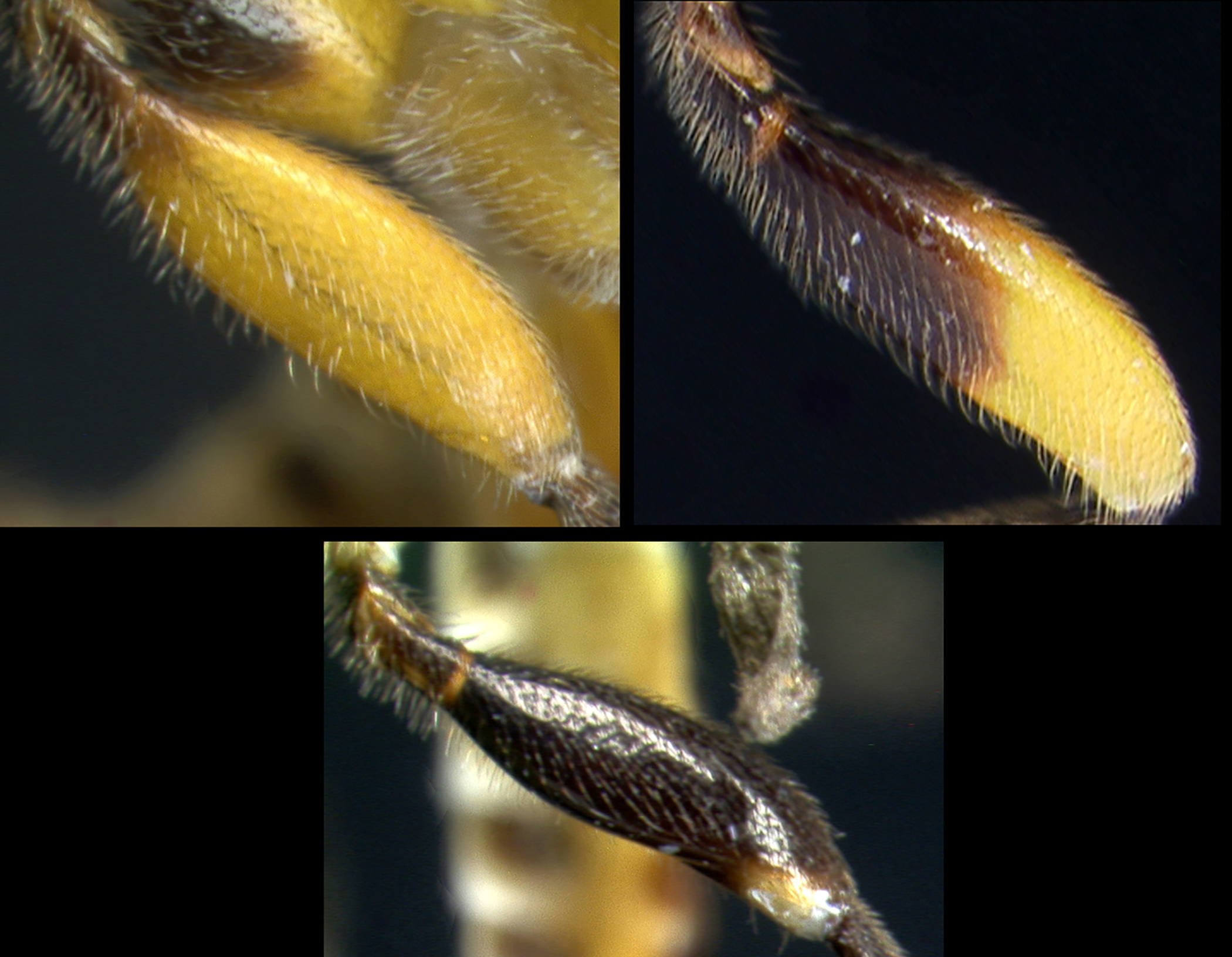

Supplement: Supplementary material 1 — Interactive key, DELTA data matrix, and images for the revision of the species of Lytopylus from Area de Conservación Guanacaste, northwestern Costa Rica (Hymenoptera, Braconidae, Agathidinae) [file zookeys-721-093-s001.zip › Revised ACG Lytopylus Interactive key/Revised Lytopylus Interactive key/images/9hindfemurcolor.jpg]

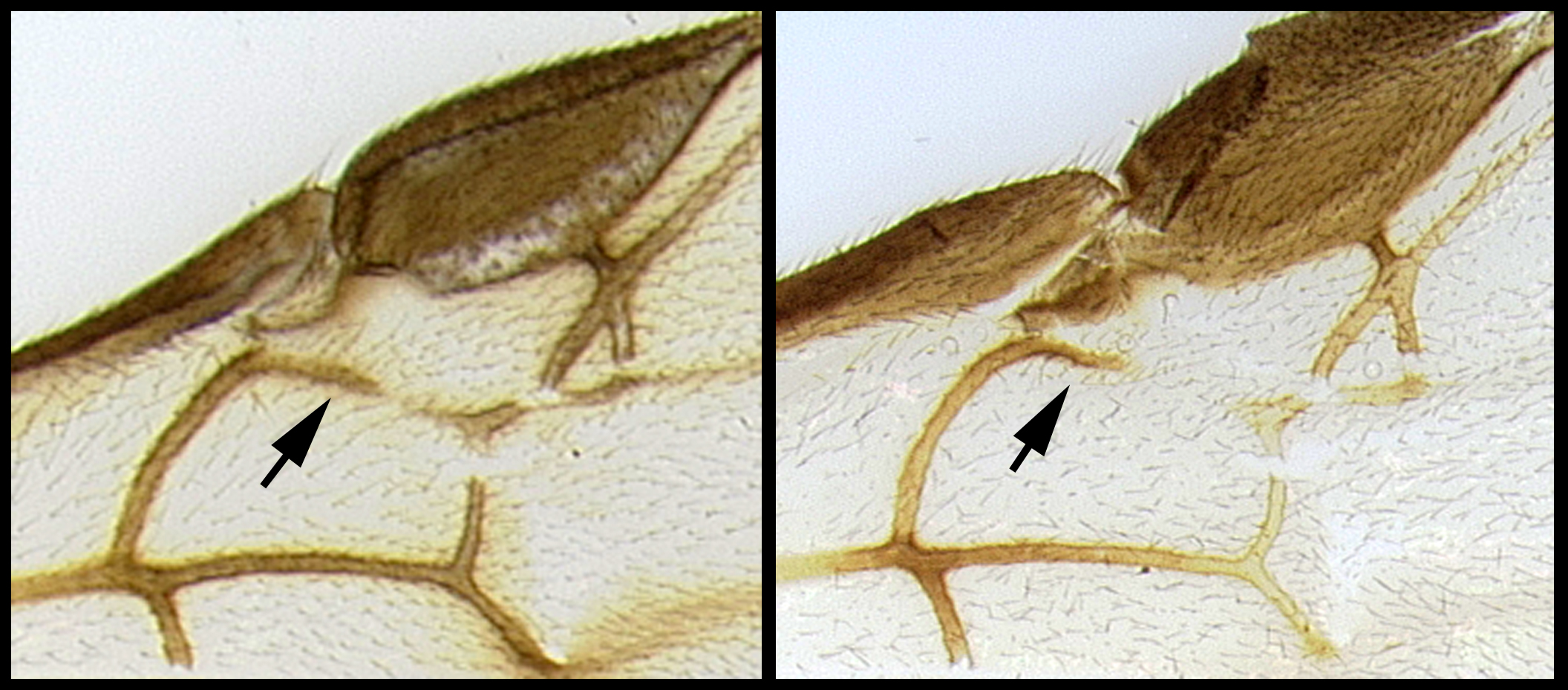

Supplement: Supplementary material 1 — Interactive key, DELTA data matrix, and images for the revision of the species of Lytopylus from Area de Conservación Guanacaste, northwestern Costa Rica (Hymenoptera, Braconidae, Agathidinae) [file zookeys-721-093-s001.zip › Revised ACG Lytopylus Interactive key/Revised Lytopylus Interactive key/images/FWRSMA.JPG]

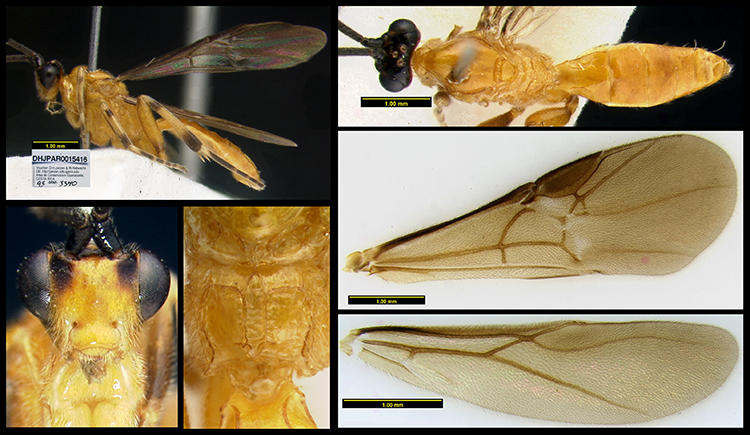

Supplement: Supplementary material 1 — Interactive key, DELTA data matrix, and images for the revision of the species of Lytopylus from Area de Conservación Guanacaste, northwestern Costa Rica (Hymenoptera, Braconidae, Agathidinae) [file zookeys-721-093-s001.zip › Revised ACG Lytopylus Interactive key/Revised Lytopylus Interactive key/images/L.alejandromasisi.jpg]

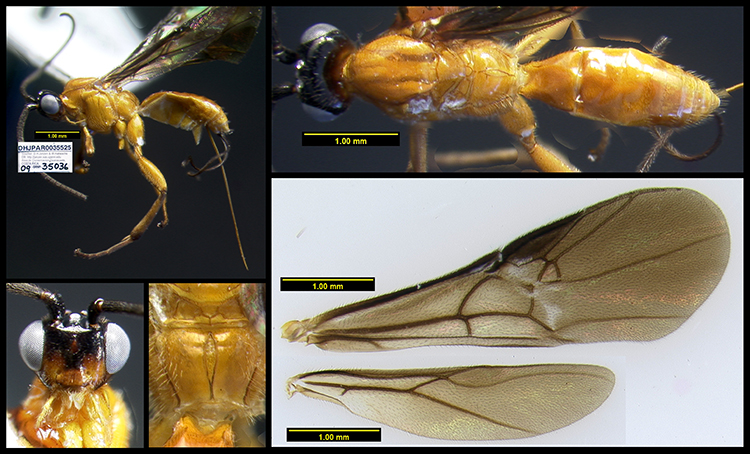

Supplement: Supplementary material 1 — Interactive key, DELTA data matrix, and images for the revision of the species of Lytopylus from Area de Conservación Guanacaste, northwestern Costa Rica (Hymenoptera, Braconidae, Agathidinae) [file zookeys-721-093-s001.zip › Revised ACG Lytopylus Interactive key/Revised Lytopylus Interactive key/images/L.alfredomainieri.jpg]

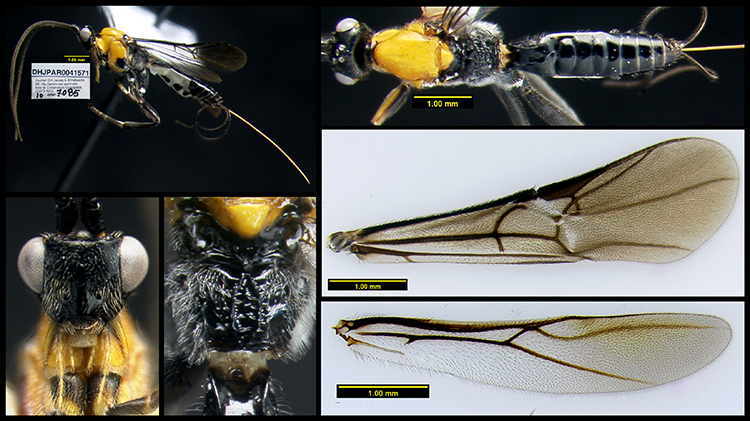

Supplement: Supplementary material 1 — Interactive key, DELTA data matrix, and images for the revision of the species of Lytopylus from Area de Conservación Guanacaste, northwestern Costa Rica (Hymenoptera, Braconidae, Agathidinae) [file zookeys-721-093-s001.zip › Revised ACG Lytopylus Interactive key/Revised Lytopylus Interactive key/images/L.anamariamongeae.jpg]

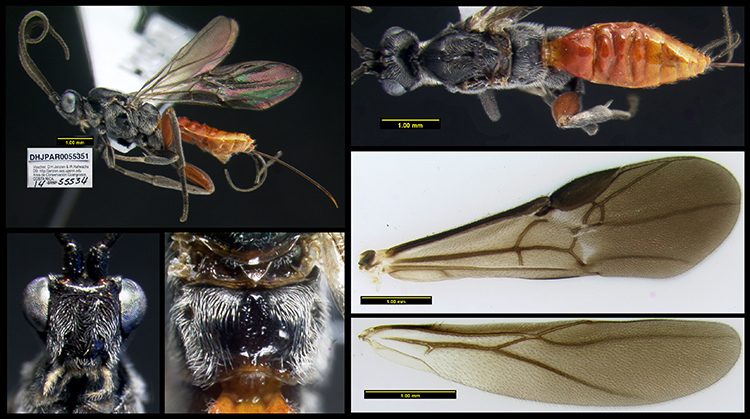

Supplement: Supplementary material 1 — Interactive key, DELTA data matrix, and images for the revision of the species of Lytopylus from Area de Conservación Guanacaste, northwestern Costa Rica (Hymenoptera, Braconidae, Agathidinae) [file zookeys-721-093-s001.zip › Revised ACG Lytopylus Interactive key/Revised Lytopylus Interactive key/images/L.angelagonzalezae.jpg]

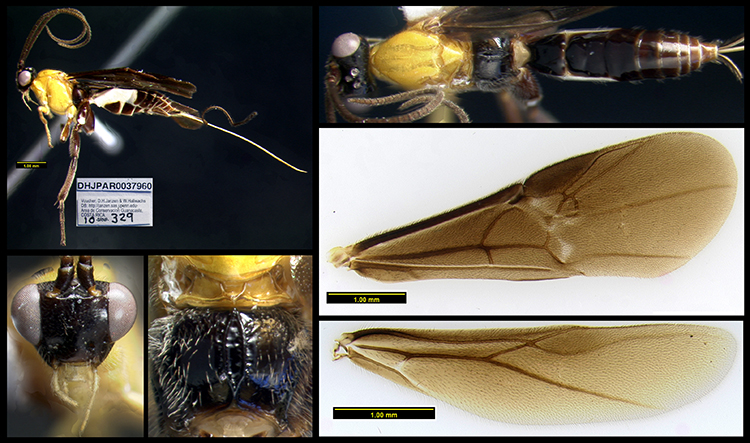

Supplement: Supplementary material 1 — Interactive key, DELTA data matrix, and images for the revision of the species of Lytopylus from Area de Conservación Guanacaste, northwestern Costa Rica (Hymenoptera, Braconidae, Agathidinae) [file zookeys-721-093-s001.zip › Revised ACG Lytopylus Interactive key/Revised Lytopylus Interactive key/images/L.cesarmorai.jpg]

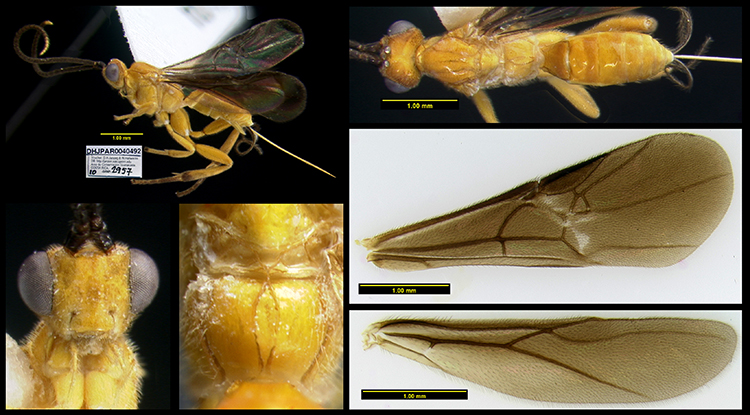

Supplement: Supplementary material 1 — Interactive key, DELTA data matrix, and images for the revision of the species of Lytopylus from Area de Conservación Guanacaste, northwestern Costa Rica (Hymenoptera, Braconidae, Agathidinae) [file zookeys-721-093-s001.zip › Revised ACG Lytopylus Interactive key/Revised Lytopylus Interactive key/images/L.chrysokeras.jpg]

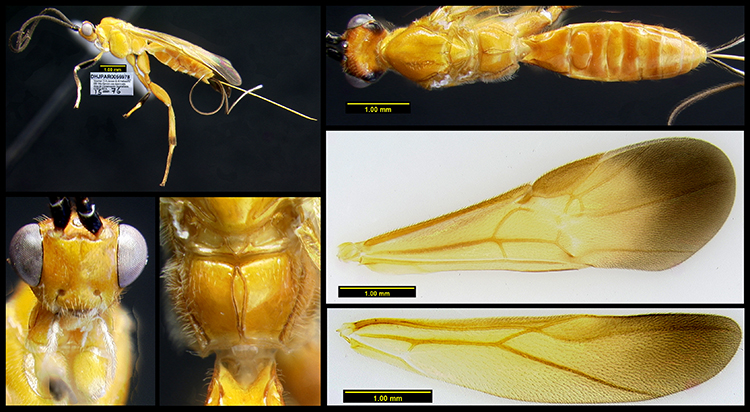

Supplement: Supplementary material 1 — Interactive key, DELTA data matrix, and images for the revision of the species of Lytopylus from Area de Conservación Guanacaste, northwestern Costa Rica (Hymenoptera, Braconidae, Agathidinae) [file zookeys-721-093-s001.zip › Revised ACG Lytopylus Interactive key/Revised Lytopylus Interactive key/images/L.eddysanchezi.jpg]

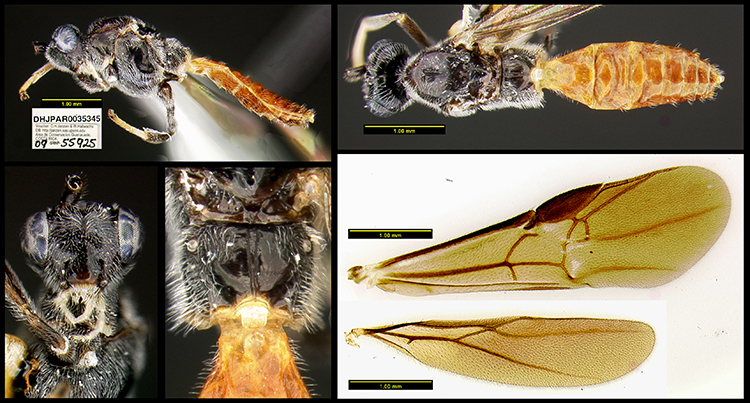

Supplement: Supplementary material 1 — Interactive key, DELTA data matrix, and images for the revision of the species of Lytopylus from Area de Conservación Guanacaste, northwestern Costa Rica (Hymenoptera, Braconidae, Agathidinae) [file zookeys-721-093-s001.zip › Revised ACG Lytopylus Interactive key/Revised Lytopylus Interactive key/images/L.eliethcantillanoae.jpg]

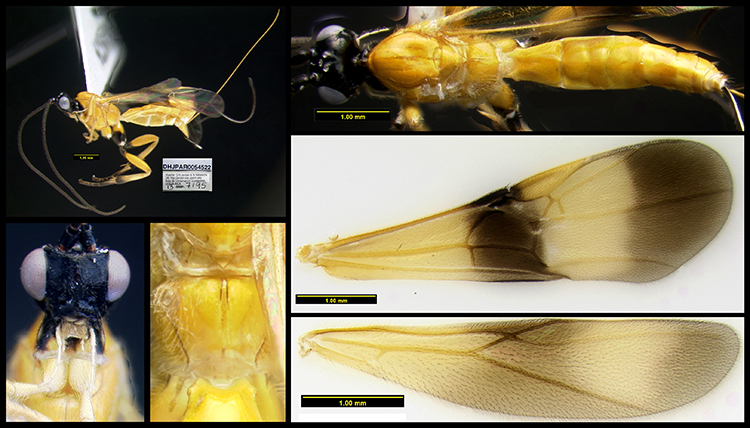

Supplement: Supplementary material 1 — Interactive key, DELTA data matrix, and images for the revision of the species of Lytopylus from Area de Conservación Guanacaste, northwestern Costa Rica (Hymenoptera, Braconidae, Agathidinae) [file zookeys-721-093-s001.zip › Revised ACG Lytopylus Interactive key/Revised Lytopylus Interactive key/images/L.ericchapmani.jpg]

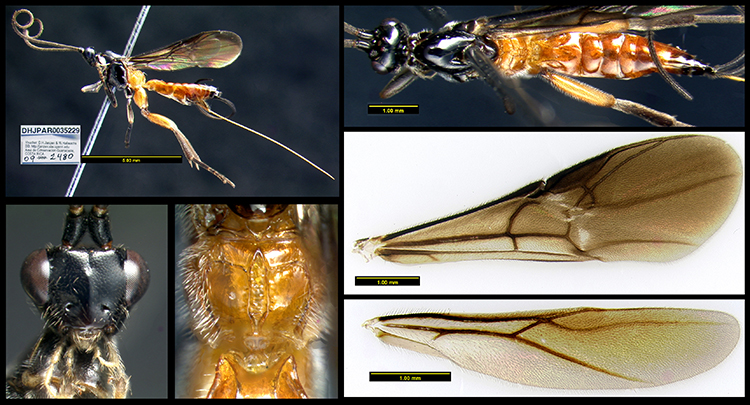

Supplement: Supplementary material 1 — Interactive key, DELTA data matrix, and images for the revision of the species of Lytopylus from Area de Conservación Guanacaste, northwestern Costa Rica (Hymenoptera, Braconidae, Agathidinae) [file zookeys-721-093-s001.zip › Revised ACG Lytopylus Interactive key/Revised Lytopylus Interactive key/images/L.gahyunae.jpg]

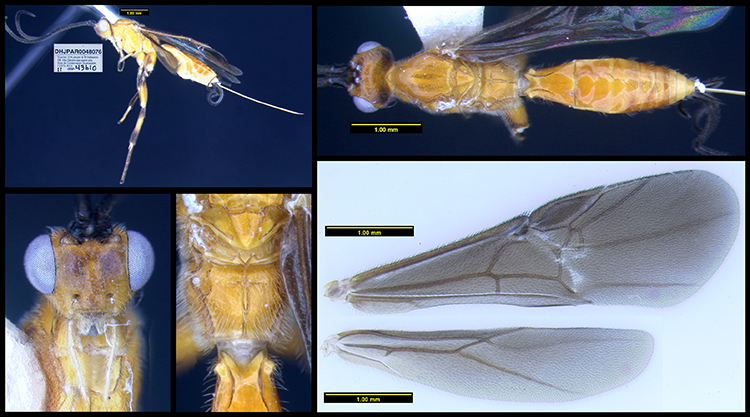

Supplement: Supplementary material 1 — Interactive key, DELTA data matrix, and images for the revision of the species of Lytopylus from Area de Conservación Guanacaste, northwestern Costa Rica (Hymenoptera, Braconidae, Agathidinae) [file zookeys-721-093-s001.zip › Revised ACG Lytopylus Interactive key/Revised Lytopylus Interactive key/images/L.gisukae.jpg]

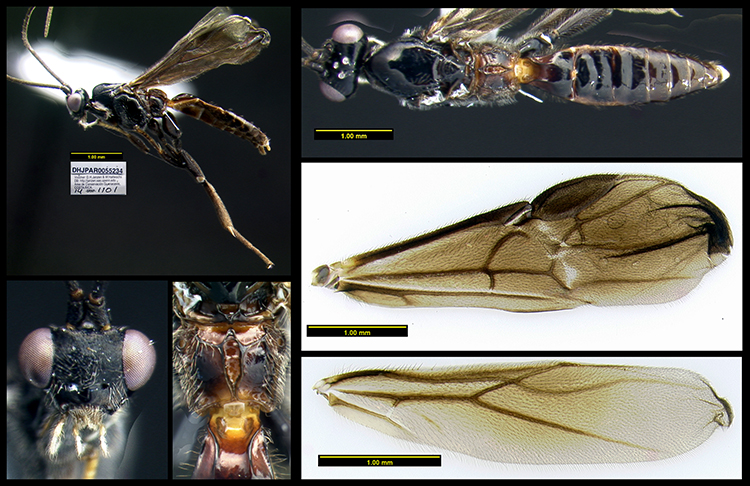

Supplement: Supplementary material 1 — Interactive key, DELTA data matrix, and images for the revision of the species of Lytopylus from Area de Conservación Guanacaste, northwestern Costa Rica (Hymenoptera, Braconidae, Agathidinae) [file zookeys-721-093-s001.zip › Revised ACG Lytopylus Interactive key/Revised Lytopylus Interactive key/images/L.guillermopereirai.jpg]

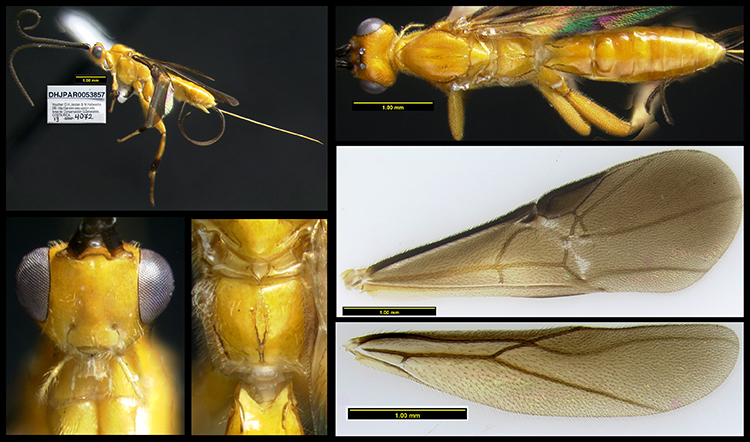

Supplement: Supplementary material 1 — Interactive key, DELTA data matrix, and images for the revision of the species of Lytopylus from Area de Conservación Guanacaste, northwestern Costa Rica (Hymenoptera, Braconidae, Agathidinae) [file zookeys-721-093-s001.zip › Revised ACG Lytopylus Interactive key/Revised Lytopylus Interactive key/images/L.gustavoindunii.jpg]

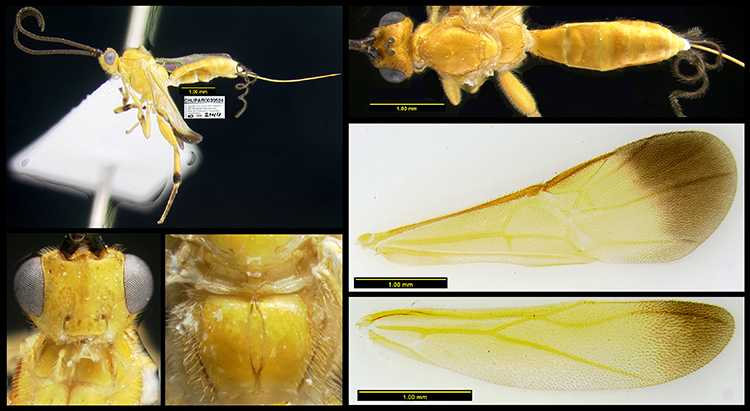

Supplement: Supplementary material 1 — Interactive key, DELTA data matrix, and images for the revision of the species of Lytopylus from Area de Conservación Guanacaste, northwestern Costa Rica (Hymenoptera, Braconidae, Agathidinae) [file zookeys-721-093-s001.zip › Revised ACG Lytopylus Interactive key/Revised Lytopylus Interactive key/images/L.hartmanguidoi.jpg]

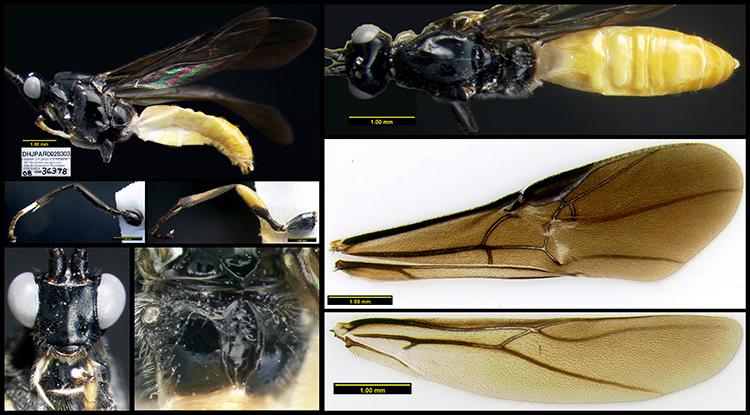

Supplement: Supplementary material 1 — Interactive key, DELTA data matrix, and images for the revision of the species of Lytopylus from Area de Conservación Guanacaste, northwestern Costa Rica (Hymenoptera, Braconidae, Agathidinae) [file zookeys-721-093-s001.zip › Revised ACG Lytopylus Interactive key/Revised Lytopylus Interactive key/images/L.hernanbravoi.jpg]

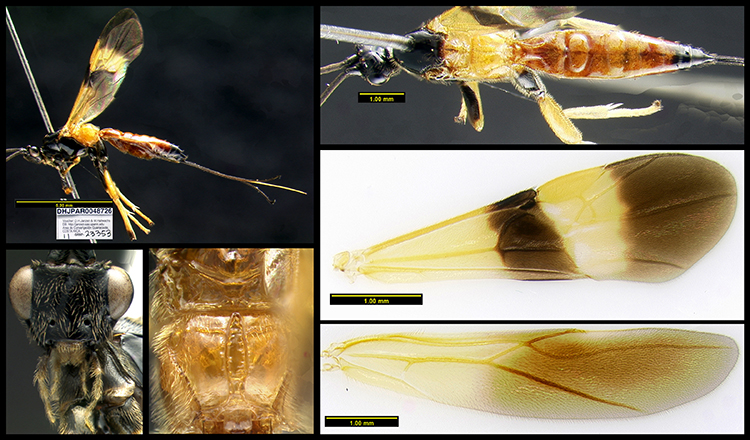

Supplement: Supplementary material 1 — Interactive key, DELTA data matrix, and images for the revision of the species of Lytopylus from Area de Conservación Guanacaste, northwestern Costa Rica (Hymenoptera, Braconidae, Agathidinae) [file zookeys-721-093-s001.zip › Revised ACG Lytopylus Interactive key/Revised Lytopylus Interactive key/images/L.hokwoni.jpg]

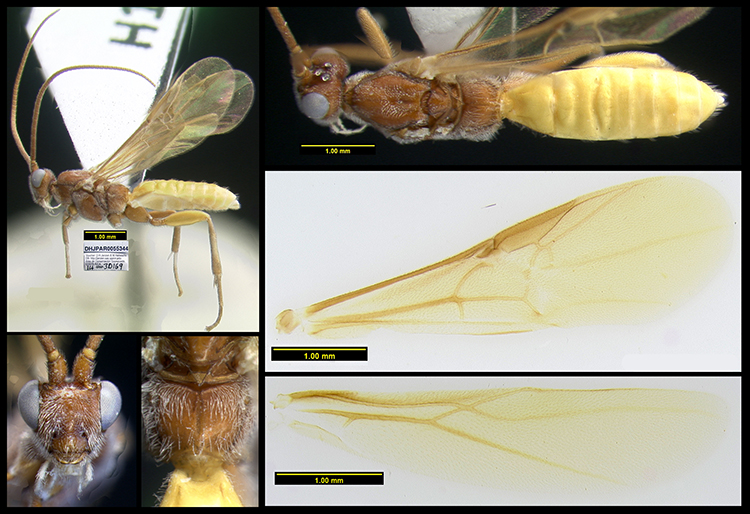

Supplement: Supplementary material 1 — Interactive key, DELTA data matrix, and images for the revision of the species of Lytopylus from Area de Conservación Guanacaste, northwestern Costa Rica (Hymenoptera, Braconidae, Agathidinae) [file zookeys-721-093-s001.zip › Revised ACG Lytopylus Interactive key/Revised Lytopylus Interactive key/images/L.ivanniasandovalae.jpg]

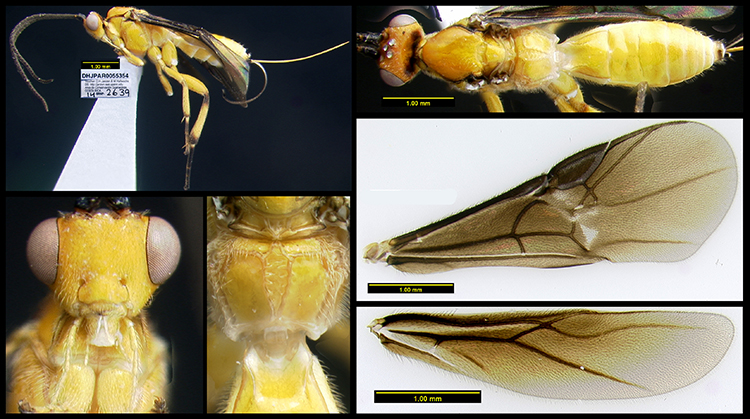

Supplement: Supplementary material 1 — Interactive key, DELTA data matrix, and images for the revision of the species of Lytopylus from Area de Conservación Guanacaste, northwestern Costa Rica (Hymenoptera, Braconidae, Agathidinae) [file zookeys-721-093-s001.zip › Revised ACG Lytopylus Interactive key/Revised Lytopylus Interactive key/images/L.johanvalerioi.jpg]

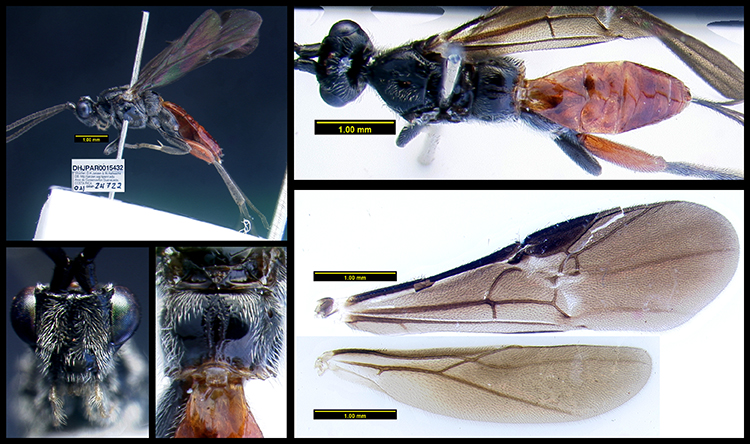

Supplement: Supplementary material 1 — Interactive key, DELTA data matrix, and images for the revision of the species of Lytopylus from Area de Conservación Guanacaste, northwestern Costa Rica (Hymenoptera, Braconidae, Agathidinae) [file zookeys-721-093-s001.zip › Revised ACG Lytopylus Interactive key/Revised Lytopylus Interactive key/images/L.josecortesi.jpg]

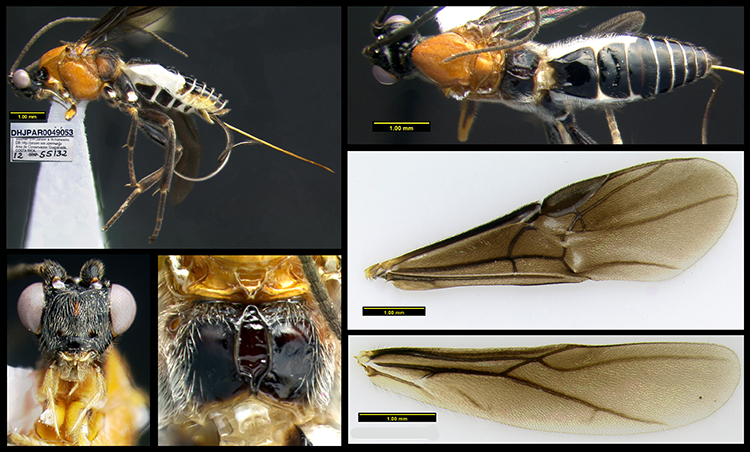

Supplement: Supplementary material 1 — Interactive key, DELTA data matrix, and images for the revision of the species of Lytopylus from Area de Conservación Guanacaste, northwestern Costa Rica (Hymenoptera, Braconidae, Agathidinae) [file zookeys-721-093-s001.zip › Revised ACG Lytopylus Interactive key/Revised Lytopylus Interactive key/images/L.luisgaritai.jpg]

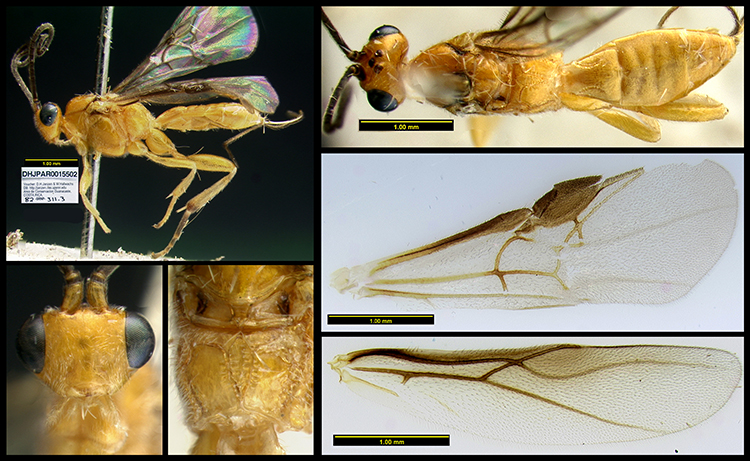

Supplement: Supplementary material 1 — Interactive key, DELTA data matrix, and images for the revision of the species of Lytopylus from Area de Conservación Guanacaste, northwestern Costa Rica (Hymenoptera, Braconidae, Agathidinae) [file zookeys-721-093-s001.zip › Revised ACG Lytopylus Interactive key/Revised Lytopylus Interactive key/images/L.mariamartachavarriae.jpg]

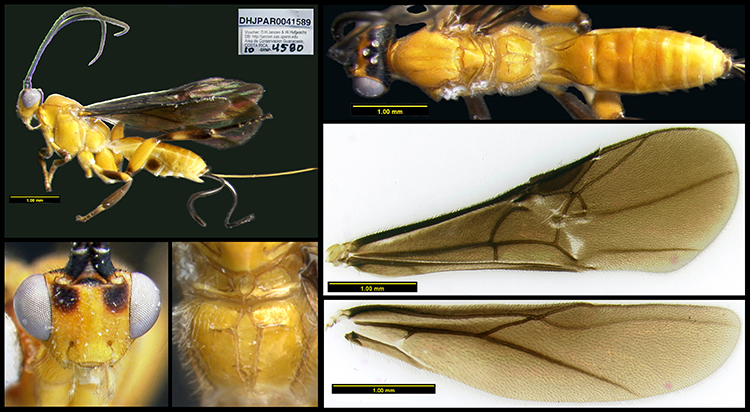

Supplement: Supplementary material 1 — Interactive key, DELTA data matrix, and images for the revision of the species of Lytopylus from Area de Conservación Guanacaste, northwestern Costa Rica (Hymenoptera, Braconidae, Agathidinae) [file zookeys-721-093-s001.zip › Revised ACG Lytopylus Interactive key/Revised Lytopylus Interactive key/images/L.miguelviquezifemale.jpg]

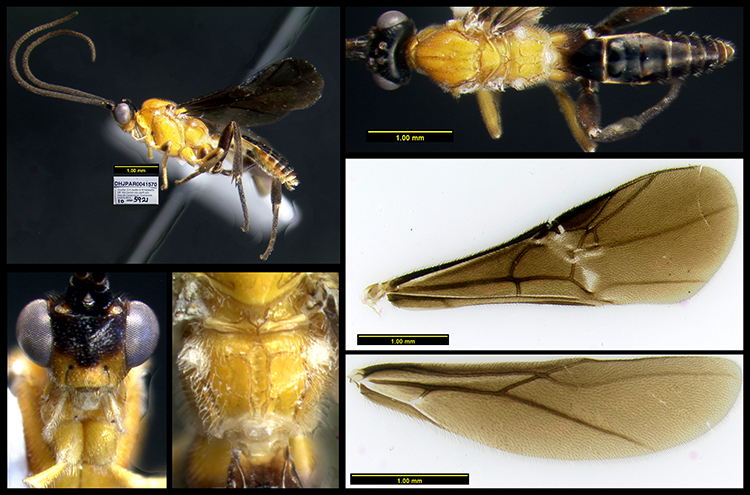

Supplement: Supplementary material 1 — Interactive key, DELTA data matrix, and images for the revision of the species of Lytopylus from Area de Conservación Guanacaste, northwestern Costa Rica (Hymenoptera, Braconidae, Agathidinae) [file zookeys-721-093-s001.zip › Revised ACG Lytopylus Interactive key/Revised Lytopylus Interactive key/images/L.miguelviquezimale.jpg]

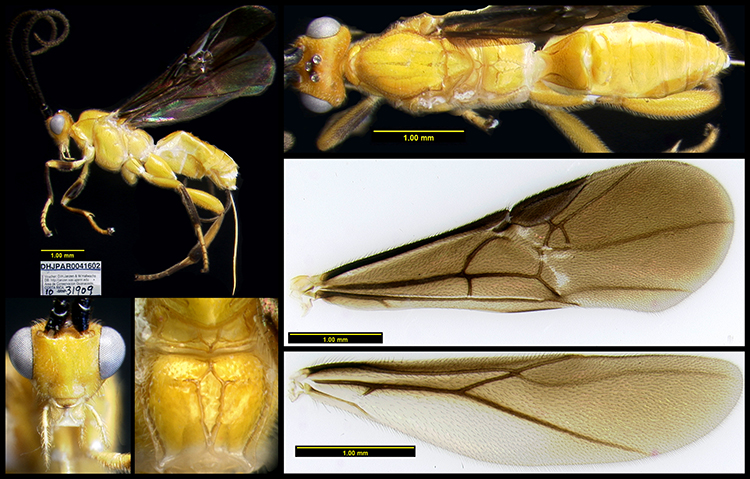

Supplement: Supplementary material 1 — Interactive key, DELTA data matrix, and images for the revision of the species of Lytopylus from Area de Conservación Guanacaste, northwestern Costa Rica (Hymenoptera, Braconidae, Agathidinae) [file zookeys-721-093-s001.zip › Revised ACG Lytopylus Interactive key/Revised Lytopylus Interactive key/images/L.motohasegawaifemale.jpg]

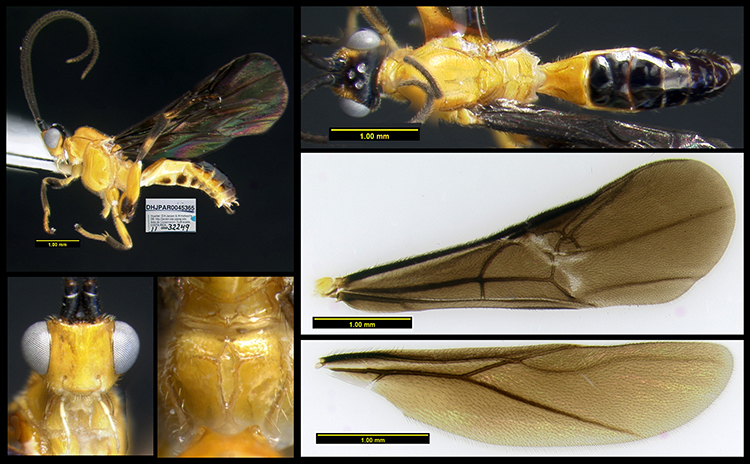

Supplement: Supplementary material 1 — Interactive key, DELTA data matrix, and images for the revision of the species of Lytopylus from Area de Conservación Guanacaste, northwestern Costa Rica (Hymenoptera, Braconidae, Agathidinae) [file zookeys-721-093-s001.zip › Revised ACG Lytopylus Interactive key/Revised Lytopylus Interactive key/images/L.motohasegawaimale.jpg]

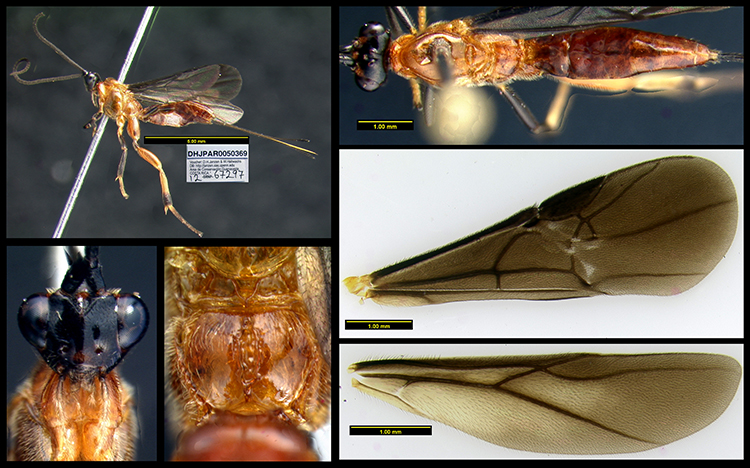

Supplement: Supplementary material 1 — Interactive key, DELTA data matrix, and images for the revision of the species of Lytopylus from Area de Conservación Guanacaste, northwestern Costa Rica (Hymenoptera, Braconidae, Agathidinae) [file zookeys-721-093-s001.zip › Revised ACG Lytopylus Interactive key/Revised Lytopylus Interactive key/images/L.okchunae.jpg]

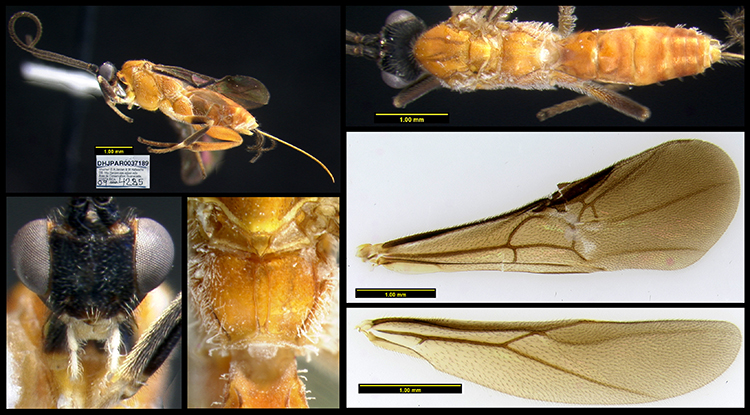

Supplement: Supplementary material 1 — Interactive key, DELTA data matrix, and images for the revision of the species of Lytopylus from Area de Conservación Guanacaste, northwestern Costa Rica (Hymenoptera, Braconidae, Agathidinae) [file zookeys-721-093-s001.zip › Revised ACG Lytopylus Interactive key/Revised Lytopylus Interactive key/images/L.pablocobbi.jpg]

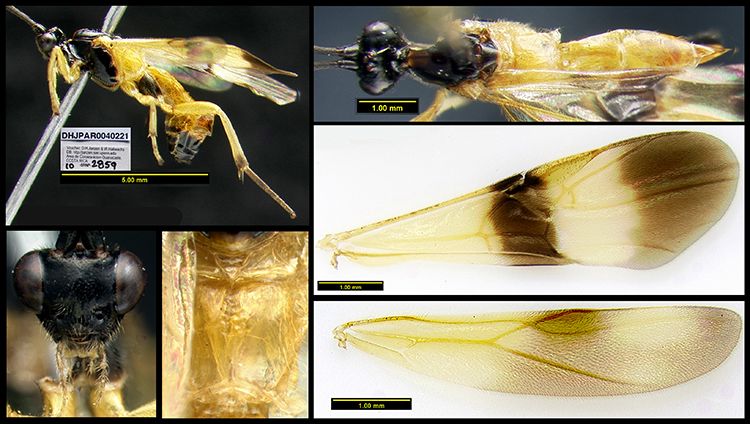

Supplement: Supplementary material 1 — Interactive key, DELTA data matrix, and images for the revision of the species of Lytopylus from Area de Conservación Guanacaste, northwestern Costa Rica (Hymenoptera, Braconidae, Agathidinae) [file zookeys-721-093-s001.zip › Revised ACG Lytopylus Interactive key/Revised Lytopylus Interactive key/images/L.robertofernandezi.jpg]

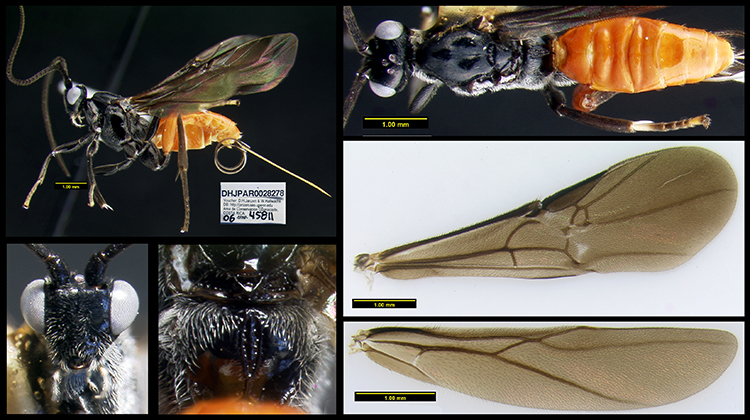

Supplement: Supplementary material 1 — Interactive key, DELTA data matrix, and images for the revision of the species of Lytopylus from Area de Conservación Guanacaste, northwestern Costa Rica (Hymenoptera, Braconidae, Agathidinae) [file zookeys-721-093-s001.zip › Revised ACG Lytopylus Interactive key/Revised Lytopylus Interactive key/images/L.rogerblancoi.jpg]

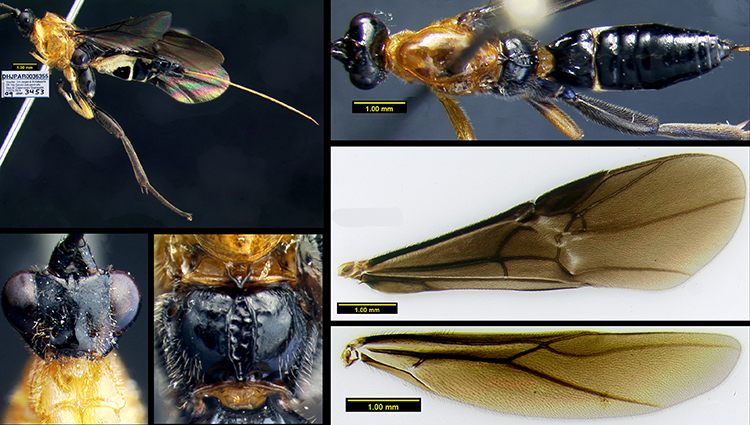

Supplement: Supplementary material 1 — Interactive key, DELTA data matrix, and images for the revision of the species of Lytopylus from Area de Conservación Guanacaste, northwestern Costa Rica (Hymenoptera, Braconidae, Agathidinae) [file zookeys-721-093-s001.zip › Revised ACG Lytopylus Interactive key/Revised Lytopylus Interactive key/images/L.salvadorlopezi.jpg]

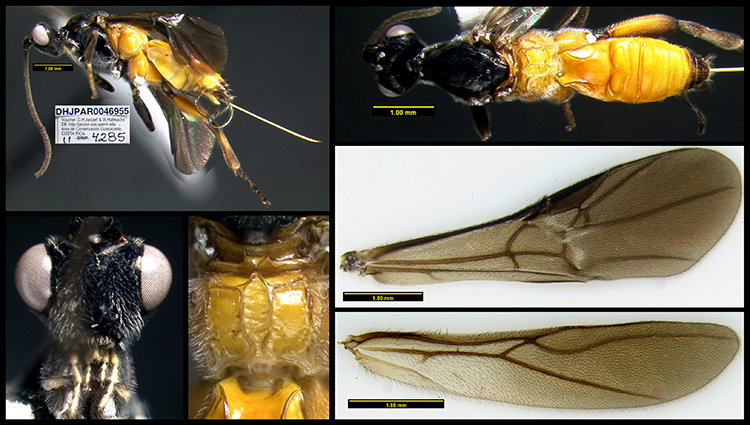

Supplement: Supplementary material 1 — Interactive key, DELTA data matrix, and images for the revision of the species of Lytopylus from Area de Conservación Guanacaste, northwestern Costa Rica (Hymenoptera, Braconidae, Agathidinae) [file zookeys-721-093-s001.zip › Revised ACG Lytopylus Interactive key/Revised Lytopylus Interactive key/images/L.sangyeoni.jpg]

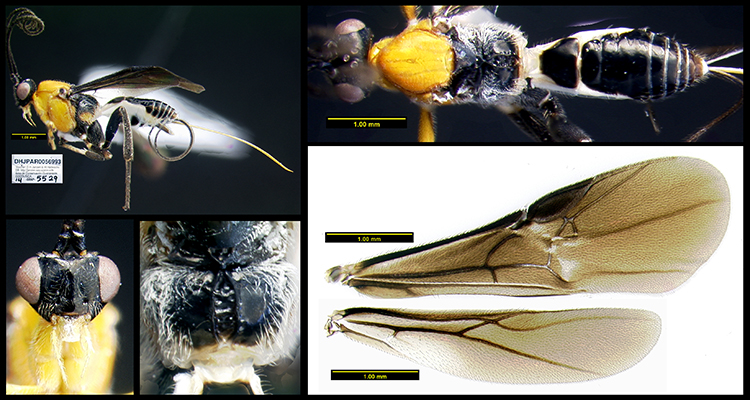

Supplement: Supplementary material 1 — Interactive key, DELTA data matrix, and images for the revision of the species of Lytopylus from Area de Conservación Guanacaste, northwestern Costa Rica (Hymenoptera, Braconidae, Agathidinae) [file zookeys-721-093-s001.zip › Revised ACG Lytopylus Interactive key/Revised Lytopylus Interactive key/images/L.sarahmeierottoae.jpg]

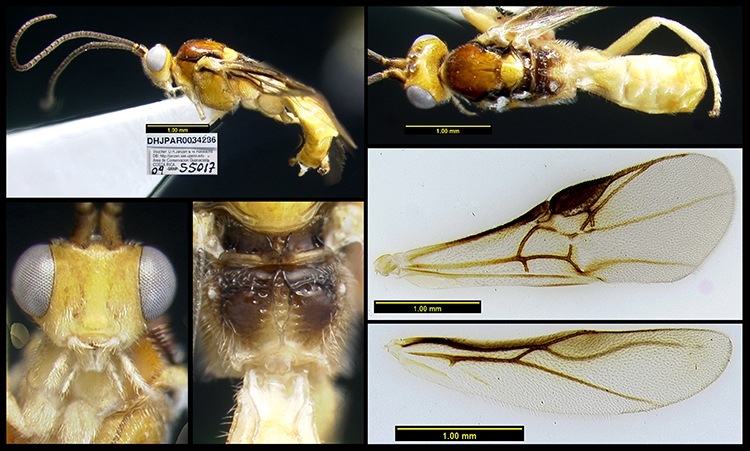

Supplement: Supplementary material 1 — Interactive key, DELTA data matrix, and images for the revision of the species of Lytopylus from Area de Conservación Guanacaste, northwestern Costa Rica (Hymenoptera, Braconidae, Agathidinae) [file zookeys-721-093-s001.zip › Revised ACG Lytopylus Interactive key/Revised Lytopylus Interactive key/images/L.sergiobermudezi.jpg]

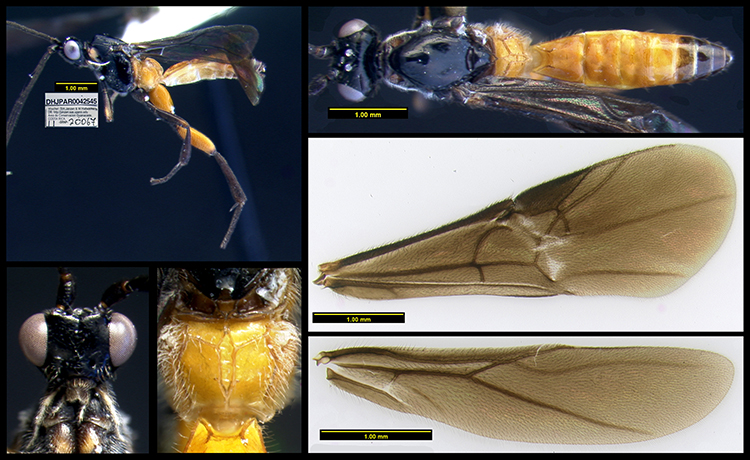

Supplement: Supplementary material 1 — Interactive key, DELTA data matrix, and images for the revision of the species of Lytopylus from Area de Conservación Guanacaste, northwestern Costa Rica (Hymenoptera, Braconidae, Agathidinae) [file zookeys-721-093-s001.zip › Revised ACG Lytopylus Interactive key/Revised Lytopylus Interactive key/images/L.sigifredomarini.jpg]

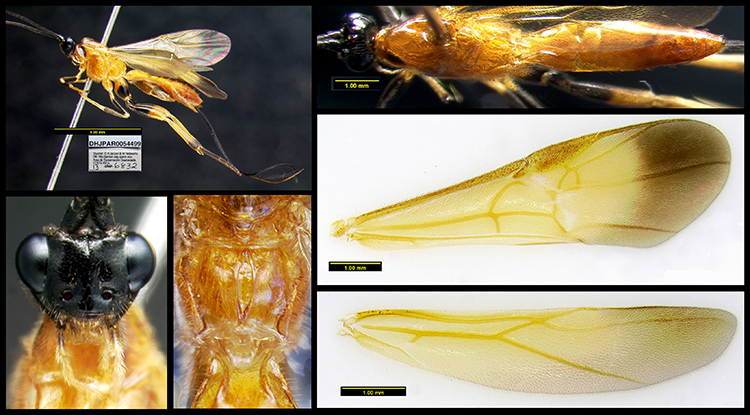

Supplement: Supplementary material 1 — Interactive key, DELTA data matrix, and images for the revision of the species of Lytopylus from Area de Conservación Guanacaste, northwestern Costa Rica (Hymenoptera, Braconidae, Agathidinae) [file zookeys-721-093-s001.zip › Revised ACG Lytopylus Interactive key/Revised Lytopylus Interactive key/images/L.youngcheae.jpg]

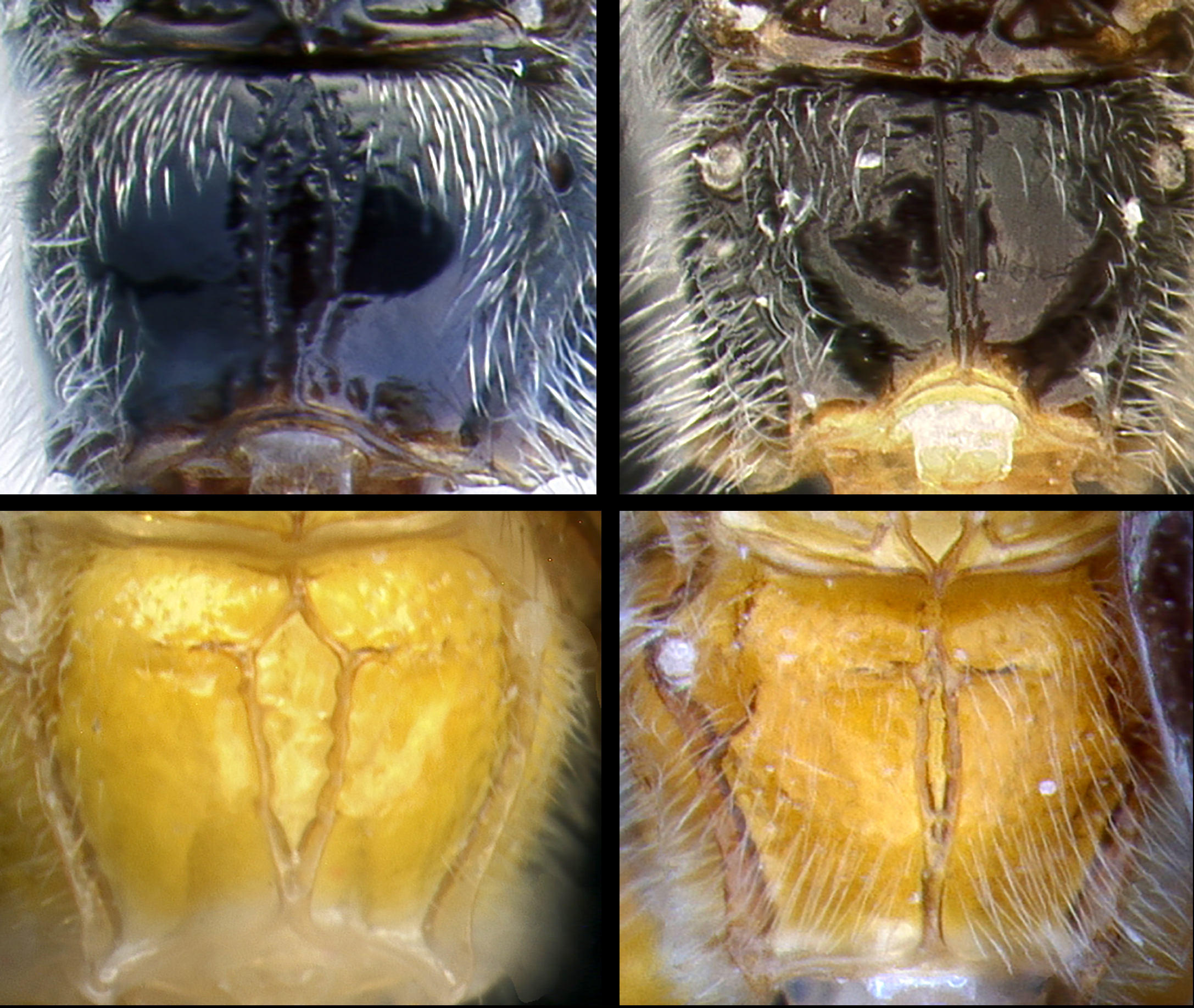

Supplement: Supplementary material 1 — Interactive key, DELTA data matrix, and images for the revision of the species of Lytopylus from Area de Conservación Guanacaste, northwestern Costa Rica (Hymenoptera, Braconidae, Agathidinae) [file zookeys-721-093-s001.zip › Revised ACG Lytopylus Interactive key/Revised Lytopylus Interactive key/images/medianareolalegthwidth.jpg]

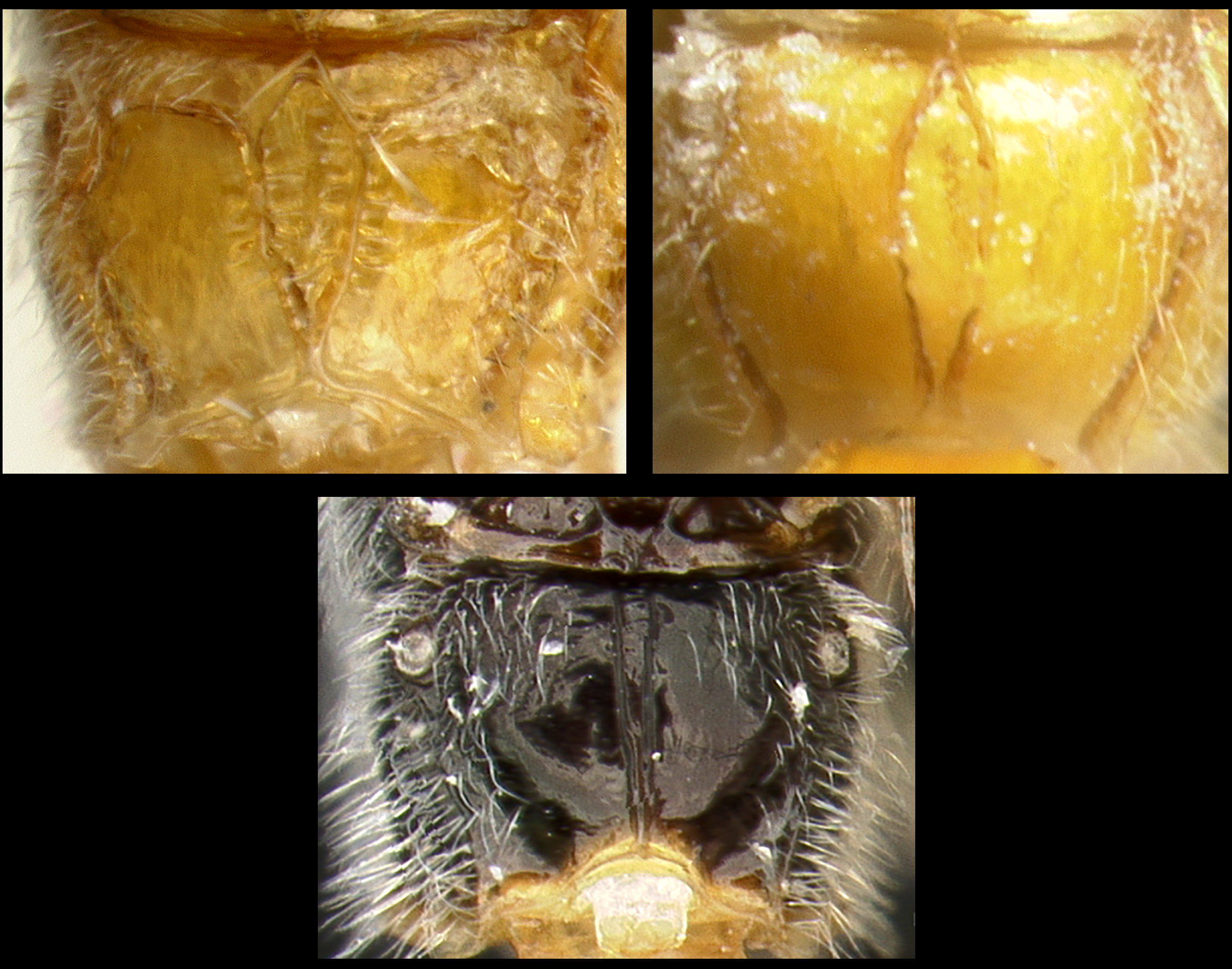

Supplement: Supplementary material 1 — Interactive key, DELTA data matrix, and images for the revision of the species of Lytopylus from Area de Conservación Guanacaste, northwestern Costa Rica (Hymenoptera, Braconidae, Agathidinae) [file zookeys-721-093-s001.zip › Revised ACG Lytopylus Interactive key/Revised Lytopylus Interactive key/images/medianareolashape.jpg]

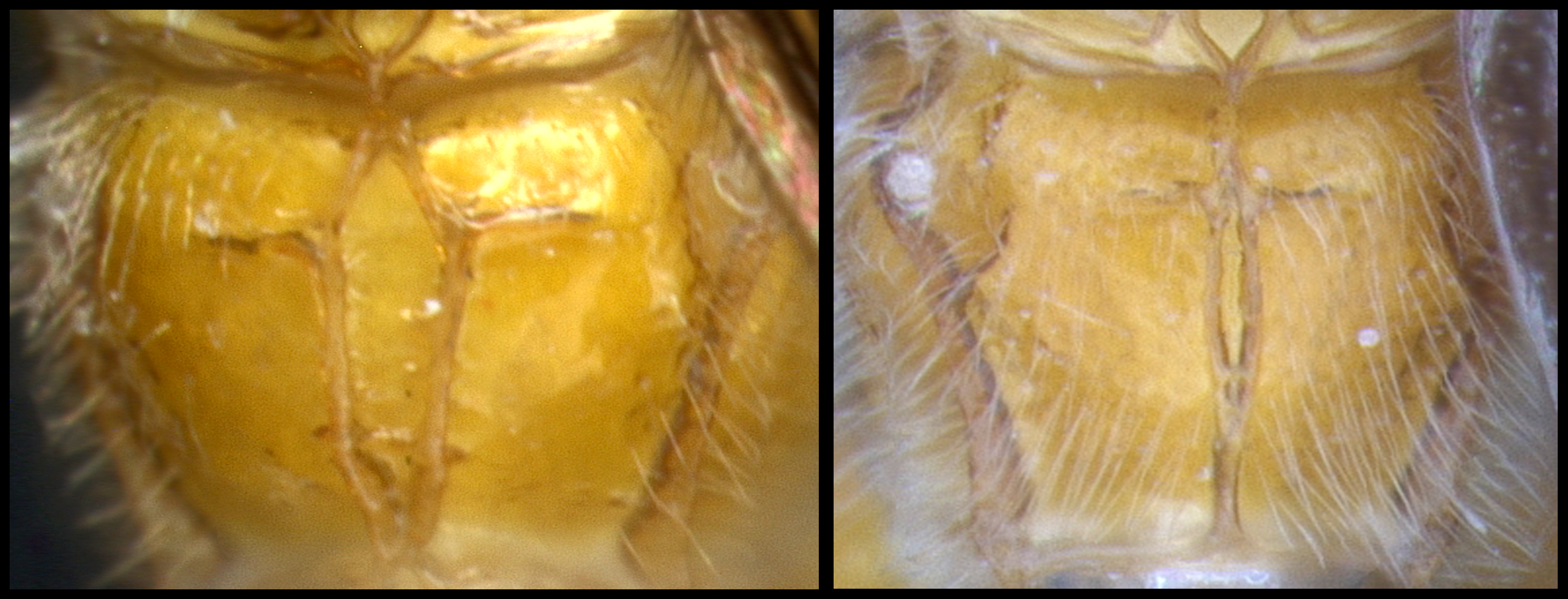

Supplement: Supplementary material 1 — Interactive key, DELTA data matrix, and images for the revision of the species of Lytopylus from Area de Conservación Guanacaste, northwestern Costa Rica (Hymenoptera, Braconidae, Agathidinae) [file zookeys-721-093-s001.zip › Revised ACG Lytopylus Interactive key/Revised Lytopylus Interactive key/images/medianareolasize.jpg]
